# Lithium-Ion Dynamic and Storage of Atomically Precise Halogenated Nanographene Assemblies via Bottom-Up Chemical Synthesis

**DOI:** 10.1021/acsami.4c02545

**Published:** 2024-05-24

**Authors:** Febri Baskoro, Hui Qi Wong, Svetozar Najman, Po-Yu Yang, Jazer Jose H. Togonon, Yi-Chi Ho, Mei-Chun Tseng, Der-Lii M. Tzou, Yu-Ruei Kung, Chun-Wei Pao, Hung-Ju Yen

**Affiliations:** † Institute of Chemistry, Academia Sinica, 128 Academia Road, Section 2, Nankang, Taipei 11529, Taiwan; ‡ Sustainable Chemical Science and Technology Program, Taiwan International Graduate Program (TIGP), Academia Sinica, Taipei 11529, Taiwan; § Department of Chemical Engineering, National Taiwan University, Taipei 10617, Taiwan; ∥ Research Center for Applied Sciences, Academia Sinica, Taipei 11529, Taiwan; ⊥ Department of Chemical Engineering and Biotechnology, Tatung University, Taipei 10452, Taiwan; # Department of Photonics, National Yang Ming Chiao Tung University, Hsinchu 30010, Taiwan

**Keywords:** nanographene, edge-functionalization, halogen, anode material, lithium-ion battery

## Abstract

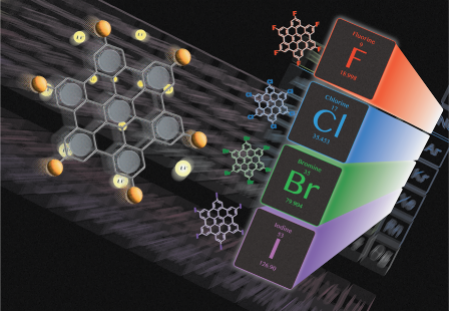

Graphene has received much scientific attention as an electrode material for lithium-ion batteries because of its extraordinary physical and electrical properties. However, the lack of structural control and restacking issues have hindered its application as carbon-based anode materials for next generation lithium-ion batteries. To improve its performance, several modification approaches such as edge-functionalization and electron-donating/withdrawing substitution have been considered as promising strategies. In addition, group 7A elements have been recognized as critical elements due to their electronegativity and electron-withdrawing character, which are able to further improve the electronic and structural properties of materials. Herein, we elucidated the chemistry of nanographenes with edge-substituted group 7A elements as lithium-ion battery anodes. The halogenated nanographenes were synthesized via bottom-up organic synthesis to ensure the structural control. Our study reveals that the presence of halogens on the edge of nanographenes not only tunes the structural and electronic properties but also impacts the material stability, reactivity, and Li^+^ storage capability. Further systematic spectroscopic studies indicate that the charge polarization caused by halogen atoms could regulate the Li^+^ transport, charge transfer energy, and charge storage behavior in nanographenes. Overall, this study provides a new molecular design for nanographene anodes aiming for next-generation lithium-ion batteries.

## Introduction

Lithium-ion batteries (LIBs) are found ubiquitous in today’s daily life ranging from portable electronics to electric vehicles (EVs), and the global market is growing and estimated to reach up to US$41.1 billion. Besides their technological advancement, the development of new electrode materials with faster electron transport, larger storage capacity, and more efficient Li-ion transport is essential to fulfill the demands of the next generation LIBs. As a negative electrode, graphite is known as the most popular anode material due to its excellent mechanical stability, electrical conductivity, cost efficiency, and abundant availability.^1^ However, graphite suffers from poor charge/discharge stability, delivering a much reduced specific capacity of ∼372 mA h g^–1^ (90% of retention) after several cycles which impedes its further application for next generation LIBs.^1−5^

Among carbon-based materials, graphene, representative of a monolayer of carbon atoms in 2D honeycomb lattice, has emerged as one of the most exciting materials while having extraordinary physical and electrical properties.^6,7^ Graphene has been found in various applications, such as nanoscale electronic devices, composite materials, sensors, solar cells, hydrogen storage, and battery electrodes.^4,8−12^ It does hold a great potential as electrode materials, with its particularly advantageous properties including high electronic conductivity, larger surface area, higher ion storage capability, and broad electrochemical window.^9,10,13−16^ It has demonstrated that the pristine graphene anode has a significantly improved specific capacity over commercial graphite given the fact that Li ions can be adsorbed on the both sides of a graphene single layer.^4^ Furthermore, graphene is enabled to facilitate rapid Li^+^ transport with a lower barrier than that of graphite as reported previously.^17^

In the graphene family, graphene oxide (GO) obtained through chemical exfoliation of graphite is the most famous precursor for preparing graphene sheets via reduction. Recently, various reduction methods such as hydrazine reduction, high-temperature pyrolysis, and electron beam irradiation have been employed to enhance the specific capacity of the resulting graphene as LIB electrodes.^18^ However, synthesis of a structurally defined graphene framework via a reduction reaction of GO still remains a challenge until present date, mainly due to the uncontrolled oxidation process and irreversible defect formation during the exfoliation of graphite.^19−22^ Moreover, significant capacity loss during charging/discharging resulting from restacking of graphene layers is considered as a major obstacle for development of graphene-based battery electrodes.^14,23−26^

To improve the electrochemical performance of graphene-based electrodes, chemical/physical doping has become one of the alternative strategies.^27^ Various doping methodologies involving heteroatoms such as nitrogen,^11,27−29^ boron,^28^ and phosphorus^30^ via heat treatment have been applied to improve Li^+^ storage capability. However, these approaches often lack structural control and thorough evaluation, and the relationship between the increased capacity and heteroatom doping has yet to be experimentally validated. Furthermore, the synthetic process for the above-mentioned materials usually requires extreme conditions (∼800 °C), significantly increasing energy consumption. Alternatively, edge-functionalization has been considered as a promising strategy to improve electrochemical properties of graphene electrodes. This strategy has been reported to increase the processability of graphene sheets, while retaining its physicochemical properties.^31−34^ Furthermore, it can also impart the chemical reactivity of the attached functional groups for specific applications.^31^ However, the research and development of edge-functionalized graphene for LIB applications is still in its infancy.

Halogenated graphenes attracted much attention since their electronic properties can be dramatically changed by the functionalization.^35−37^ Halogens, located in group VIIA of the periodic table, exhibit the characteristic of high electronegativity. For instance, fluorine (F) is the most electronegative (4.0) element, and the carbon–fluorine (C–F) bond strength (488 kJ mol^–1^) is the strongest single covalent bond.^15,38^ Also, attaching a fluorine atom into an sp^2^ carbon could lead the hybridization to sp^3^, thus significantly impacting the electronic properties and local structures while preserving the 2D hexagonal symmetry. Furthermore, the charge carrier mobility has also been reported to be 3 orders of magnitude smaller for fluoro-graphene than that of pristine graphene.^35^ Of particular interest, in similar to LIB electrode materials, the halogenated nanographenes (NGs) are able to facilitate faster Li^+^ transport during insertion/deinsertion and stabilizing the host structure during electrochemical cycling.^9,15,38−40^ Nevertheless, despite the advancements in the electrode materials developments, halogenated graphenes with well-defined structures as LIB anodes are rather limited. Earlier studies have reported successful halogenation at the edges of graphene through ball-milling of graphite with various halogen gases, demonstrating enhanced performance as LIB anodes.^9,15^ A high specific capacity of 650 mA h g^–1^ could be achieved by edge-fluorinated graphene at 0.25 A g^–1^, and it maintained a moderate stability up to 500 charge/discharge cycles with 76.6% capacity retention. The increased specific capacity is associated with the strong C–F bond maximized charge polarization, as well as enhanced chemical stability.^15^

Although this method is considered as a simple approach to generate a large variety of edge-functionalized graphene, the shear forces generated between the high-speed rotating balls could also cause random mechanochemical functionalization on the surface of the broken graphite particles. Consequently, the relationship between increased capacity and edge-functionalization is difficult to define due to the lack of structural control. According to these pioneer studies, the structurally defined and optimal electronic structure of conjugated graphene set a foundation for the development of precisely functionalized graphenes as next-generation LIB anode materials. Therefore, it is essential to develop structurally defined graphenes with edge-functionalization to elucidate their impact on the Li-ion storage capability.

**Figure 1:**
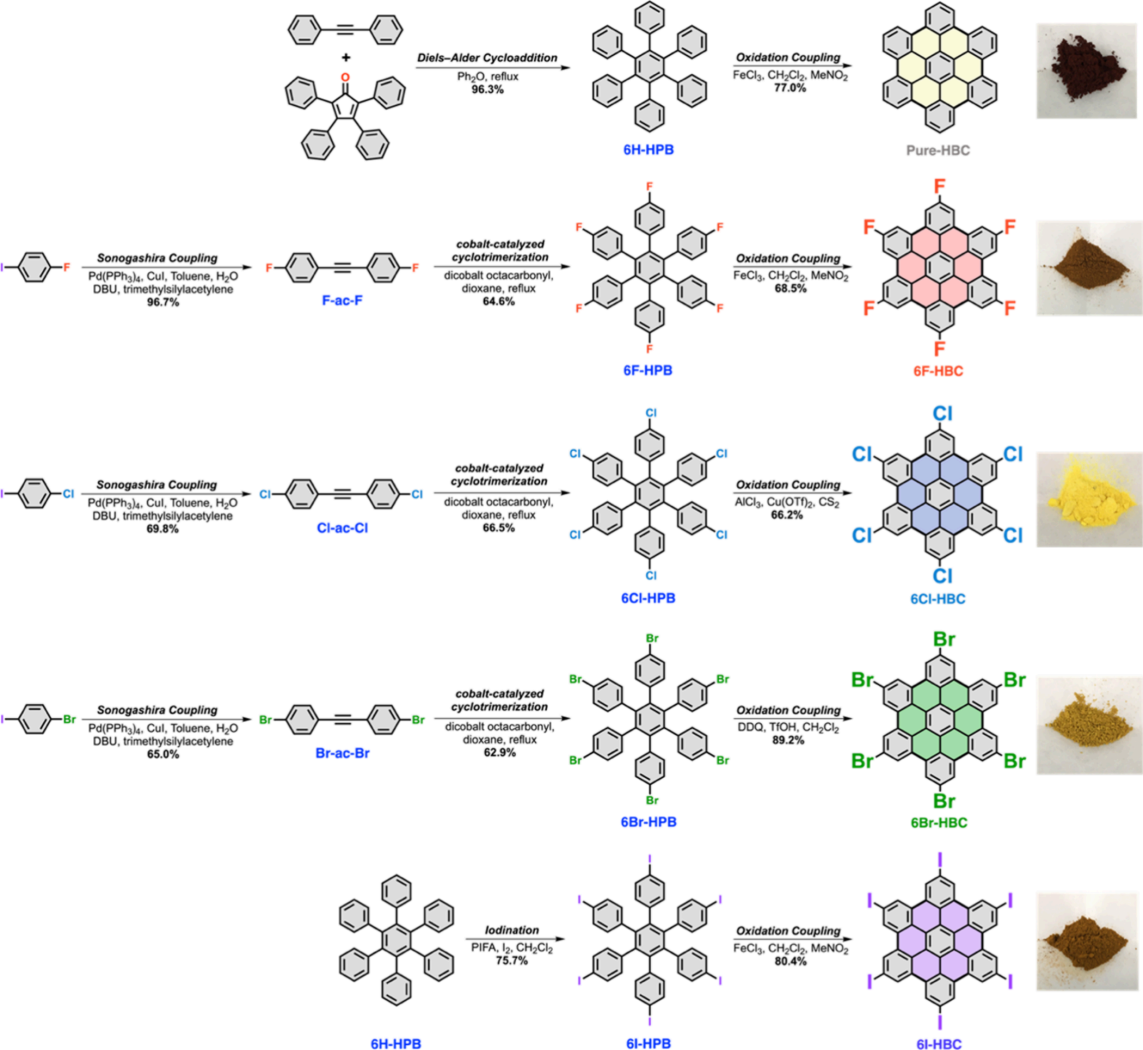
Synthetic routes and optical images of 2D NGs. **Pure-HBC**: Hexa-*peri*-hexabenzocoronene. **6F-HBC**: Hexafluoro-hexa-*peri*-hexabenzocoronene. **6Cl-HBC**: Hexachloro-hexa-*peri*-hexabenzocoronene. **6Br-HBC**: Hexabromo-hexa-*peri*-hexabenzocoronene. **6I-HBC**: Hexaiodo-hexa-*peri*-hexabenzocoronene.

In this work, we have developed a series of structurally defined NGs with edge-substituted group 7A elements via a bottom-up organic synthesis approach. A variety of group 7A elements, namely, fluorine (F), chlorine (Cl), bromine (Br), and iodine(I), have been successfully attached to the NG flakes (Figure 1), enabling tunable optimum geometric and electronic properties for achieving efficient Li-ion storage capability.

## Results and Discussion

### Material Synthesis and Characterization

**Figure 2:**
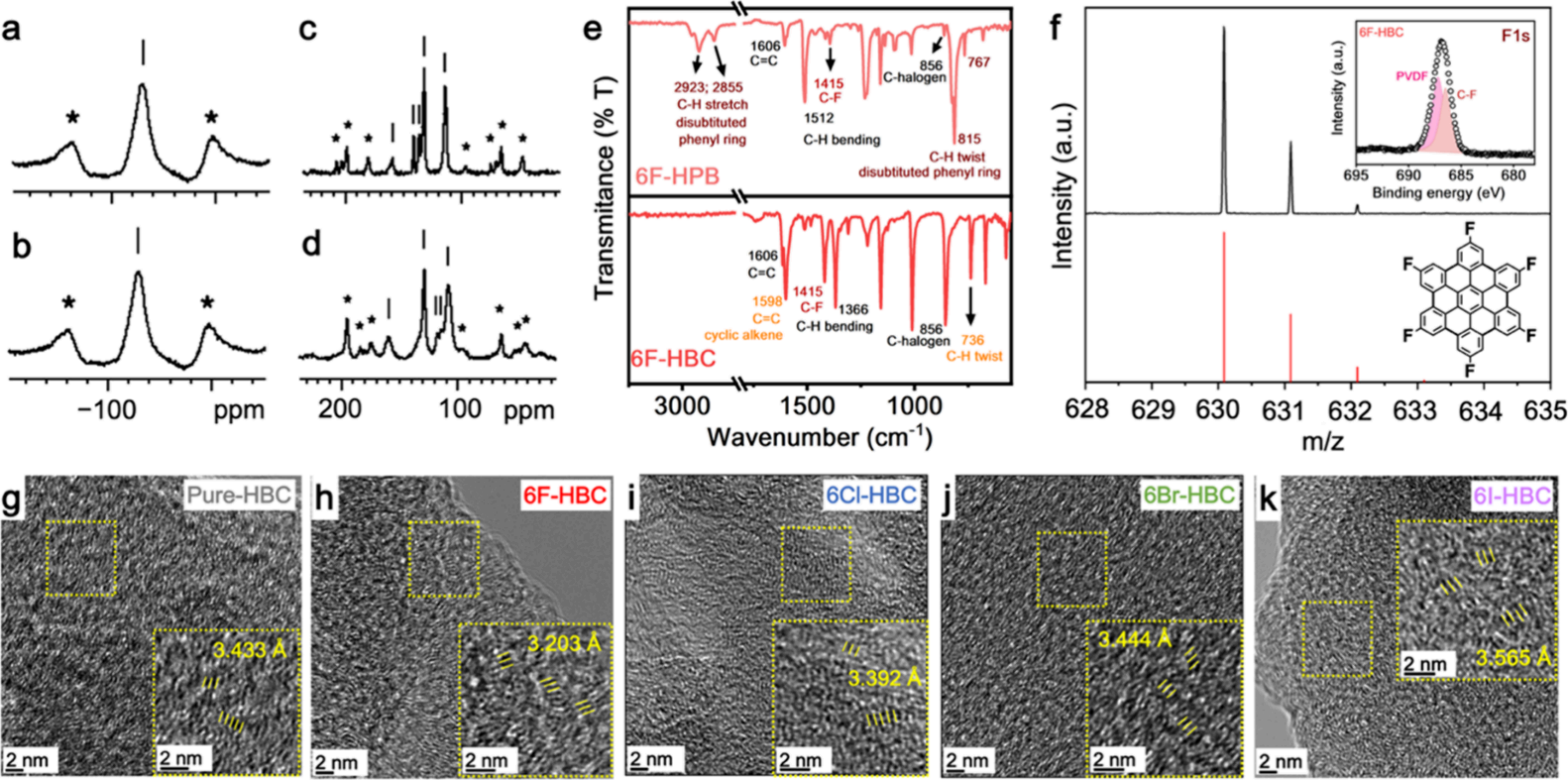
Materials characterization. Solid-state ^19^F MAS NMR of (a) **6F-HPB** and (b) **6F-HBC**; solid-state ^13^C^1^H CP/MAS NMR of (c) **6F-HPB** and (d) **6F-HBC**; the isotropic chemical shifts (labeled by vertical bars) were flanked with a set of MAS spinning sidebands (asterisks). (e) FT-IR of **6F-HPB** and **6F-HBC**. (f) MALDI-TOF MS isotopic distribution and comparison with theoretically predicted spectrum (red column) for **6F-HBC**, as well as its XPS F 1s spectrum (inset). (g–k) TEM images of **Pure-HBC**, **6F-HBC**, **6Cl-HBC**, **6Br-HBC**, and **6I-HBC**, respectively. The *d*-spacing is determined by estimating the distance between the two yellow lines (inset figure).

The synthesis of 2D edge-halogenated NGs begin with small-molecule precursors, such as 1-fluoro-4-iodobenzene, 1-chloro-4-iodobenzene, 1-bromo-4-iodobenzene, and other substituted benzene derivatives, to synthesize key intermediates. The later cobalt-catalyzed trimerization leads to polyphenylene dendritic precursors for NGs, which are then planarized via Scholl reaction, yielding the NGs. After quenching the reaction with methanol, repetitive dissolution and precipitation with dichloromethane/methanol yielded NGs as yellow to orange or brown solid powders. All of the reaction intermediates were purified and confirmed with NMR spectroscopy (see Figures S1–S7). The target NGs were characterized by solid-state NMR and MALDI-TOF mass spectrometry (see Figure 2, Figures S8–S12). As seen in Figure 2a–d, the ^19^F MAS NMR spectra of **6F-HPB** and **6F-HBC** indicate rather similar isotropic chemical shifts resonating at −114.85 and −114.35 ppm, respectively, with a mild deviation of 0.5 ppm. On the other hand, the ^13^C{^1^H} CP/MAS spectra of **6F-HPB** and **6F-HBC** (Figure 2c–d) resolve good distinct chemical shift patterns, in which that of **6F-HPB** revealed ^13^C signals at 159.49, 141.17, 137.07/136.15, 132.23, and 114.22 ppm, whereas that of **6F-HBC** exhibits intensities at 160.04/158.45, 128.86, 117.52, 114.15, and 108.40 ppm. In contrast, both the ^13^C spectra of **6Cl-HPB** and **6Cl-HBC** resolve good distinct chemical shift patterns (Figure S8), in which that of **6Cl-HPB** reveals signals at 140.44, 138.42, 133.76/132.96, 132.39, and 128.80 ppm, and that of **6Cl-HBC** exhibits rather similar signals at 140.44, 138.48, 133.64/132.96, 132.27, and 128.67 ppm.

In addition to the confirmation of fluorine-substitution on **6F-HBC** by solid-state ^19^F MAS NMR spectroscopy, MALDI-TOF was further employed to elucidate the isotopic distribution with theoretically predicted spectrum for a better understanding. The MALDI-TOF MS spectra of halogenated NGs summarized in Figures S9–S12 show not only a high accuracy with predicted structure, but also a perfect consistency with the isotopic distribution, generated from halogen atoms, with the theoretically predicted ones. For instance, **6Cl-HBC** shown in Figure S10 suggests possible stable isotopes, ^35^Cl and ^37^Cl, showing a more complex spectrum than **6Br-HBC** and **6I-HBC**, but still distinguishable and well matched with the theoretically predicted one. Another similar isotopic distribution of **6Br-HBC** with possible stable isotopes, ^79^Br and ^81^Br, was also found to be of relatively high accuracy and consistency (Figure S11). Additionally, Fourier-transform infrared spectroscopy (FT-IR) was also performed to evaluate the structural information (see Figure S13). As shown in Figure S13a, three vibrational spectra can be monitored at 3076, 3055, and 3025 cm^–1^ associated with C–H stretching of the monosubstituted phenyl ring, along with two peaks located at 728 and 695 cm^–1^ for C–H twisting of **6H-HPB**. These peaks disappear after oxidation coupling to form **Pure-HBC**, depicting new vibrational spectra of C=C (1586 cm^–1^) and C–H twisting of *o*-trisubstituted phenyl rings (759 and 736 cm^–1^). This further suggests successful formation of **Pure-HBC** and is consistent with a previous study.^41^ Furthermore, similar behavior can also be observed for **6F-HBC**, **6Cl-HBC**, **6Br-HBC**, and **6I-HBC** (Figure S13b–e), suggesting successful formation of halogenated NGs with C-halogen bond stretching at around 855 cm^–1^. The thermal stability of halogenated NGs was examined by TGA in a nitrogen atmosphere and summarized in Figure S14. All the NGs exhibited good thermal stability with insignificant weight loss up to 400 °C even with the introduction of halogen atoms.

X-ray photoelectron spectroscopy (XPS) was employed to characterize the chemical bonding in the 2D halogenated NG electrodes (Figure 2f and Figure S15). As shown Figure S15a, three features with binding energy (BE) at 284.15, 284.75, and 290.20 eV can be monitored in the C 1s spectra of **Pure-HBC**, associated with the specific BE of C=C, C–C, and π–π shakeup satellite of the benzene ring. Additionally, the specific BE of CH_2_–CF_2_ (for PVDF binder) can be observed at 286.05 and 687.45 eV on the C 1s and F 1s spectra, respectively. Furthermore, a new feature associated with the C–F can be monitored on **6F-HBC** with BE at 289.3 and 686.5 eV on the C 1s and F 1s spectra, respectively, suggesting successful attachment of F atom on the edge of NGs (Figure 2f inset and Figure S15b). A successful attachment of a Cl atom can also be confirmed by the additional new peak of C–Cl at 287.8 eV in the C 1s spectra and a doublet separation peak of C–Cl 2p_1/2_ (201.4 eV) and C–Cl 2p_3/2_ (199.8 eV) on the Cl 2p spectra (Figure S15c). Meanwhile, the C–Br formation at 286.7 eV in the C 1s spectra and doublet separation peak at 69.7 eV (C–Br 3d_5/2_) and 70.6 eV (C–Br 3d_3/2_) in the Br 3d spectra can also be observed, indicating the formation of **6Br-HBC** (Figure S15d). Additionally, the **6I-HBC** formation was confirmed by the appearance of C–I feature with BE at 285.4 and 620.1 eV on the C 1s and I 3d_5/2_ spectra, respectively (Figure S15e). Notably, the BE shift of C-halogen toward a lower value in the C 1s spectra is attributed greatly to the influence from the electronegativity of the halogen atoms.

To provide a better understanding of the halogenation effect on the structural properties, powder XRD measurement was performed to evaluate the molecular stacking of halogenated NGs (see Figure S16). As shown in Figure S16a, three diffraction peaks can be observed for **Pure-HBC** at 2θ of 7.4°, 13.3°, and 15.4°, which are associated with the (100), (110), and (200) reflections, respectively. These diffraction patterns further indicate a two-dimensional hexagonal symmetry with space group of *P6mm* and are consistent with previous reports.^41,42^ Additionally, a strong diffraction peak at 2θ of 25.9° (interlayer spacing (*d*-spacing) = 3.43 Å) can be observed, corresponding to the π–π stacking distance between two overlapping NGs molecules (inset Figure S16a). Furthermore, significant changes can be monitored when an F atom is attached to NG edges (Figure S16b). Multiple diffraction peaks can be monitored at 2θ of 7.3°, 12.4°, 13.4°, 18.0°, 19.1°, and 20.3°, associated with (110), (020), (310), (130), (420), and (510) reflections, respectively. This pattern further suggests a monoclinic symmetry with space group of *C*2*/m*, where the **6F-HBC** form a planar layered structure and is in good agreement with a previous investigation.^43^ Interestingly, the peak associated with the π–π stacking was shifted to a higher angle of 26.7° for **6F-HBC** (Figure S16b), resulting in a reduced π–π stacking distance between **6F-HBC** molecules from 3.43 to3.31 Å. This molecular rearrangement could be associated with the high electronegativity of F atom on the edges of NGs. Generally, the electron density in **Pure-HBC** is rather high in the molecular core and partially depleted in the molecular rim.^44^ In contrast, this situation is significantly inverted in the **6F-HBC**. In the **6F-HBC**, the electron density in the core molecule was significantly reduced while drastically increased at the molecular rim due to the strong electronegative F on the edge. This significant charge polarization possibly creates strong molecular dipole moments that act as mediators in the stacking formation process.^44^

Furthermore, the diffraction peak ascribed to the stacking NGs was split into two diffraction peaks when a Cl or Br is introduced on the edge of NGs (Figure S16c and d). Notably, the specific diffraction pattern of the monoclinic symmetry in **6F-HBC** disappears in **6Cl-HBC** and **6Br-HBC**, suggesting a significant change in the molecular arrangement. As shown in Figure S16c, the **6Cl-HBC** has two diffraction peaks associate to the stacking NGs at 26.05° (*d*-spacing = 3.41 Å) and 27.31° (*d*-spacing = 3.25 Å), as well as the new diffraction peak appearing at 17.50° (*d*-spacing = 5.06 Å). This splitting diffraction peak of stacking orientation could be associated with the increased atomic radius (99 pm) and reduced electronegativity (3.0) of a Cl atom than that of a F atom (77 pm/4.0), which could possess a torsional degree of freedom, affect the nearest π-conjugation, create negative distortions, and drive the molecule out of planarity.^45^ Similar to that of **6Cl-HBC**, **6Br-HBC** also has two diffraction peaks in association with the stacking orientation (Figure S16d). These peaks in **6Br-HBC** are slightly shifted to a lower angle of 25.74° (*d*-spacing = 3.45 Å) and 26.87° (*d*-spacing = 3.31 Å) than **6Cl-HBC**, due to the increasing atomic radius and reducing electronegativity of Br atom (114 pm/2.8). Notably, this peak shifting to a lower angle indicates the broadening distance between two NGs and possibly loosen the π–π stacking. Furthermore, **6I-HBC** possesses a significantly different diffraction pattern among all halogen-functionalized NGs. As shown in Figure S16e, a broad peak can be observed at 15–35° with the peak at 24.81° (*d*-spacing = 3.58 Å), indicating a highly enlarged interlayer spacing of NGs and formation of amorphous-like structures due to the increased atomic radius of I atom (133 pm). To verify this phenomena, a series of density functional theory (DFT) simulations have been performed for various structures to determine the effect of halogen on the edge of NGs (see Supplementary Note 6) and have been discussed later in the mechanistic studies.

TEM analysis was also been performed to evaluate the stacking orientation of halogen-functionalized NGs. As shown in Figure 2g–k, self-assembled domains can be monitored in all NGs, indicating the π–π interaction between neighboring NG flakes. Furthermore, the TEM images indicated a change in the *d*-spacing between self-assembled flakes due to different edge-functionalization (Figure 2g–k). The NGs with hydrogen (**Pure-HBC**), fluoro (**6F-HBC**), chloro (**6Cl-HBC**), bromo (**6Br-HBC**), and iodo (**6I-HBC**) functional groups attached on the edges of flakes have corresponding interlayer spacings of 3.433, 3.203, 3.392, 3.444, and 3.565 Å, respectively. Interestingly, **6F-HBC** has the smallest *d*-spacing than other NGs (Figure 2h), indicating the strong charge polarization on the NG basal plane which creates molecular dipole moments and acts as a mediator to reduce the *d*-spacing. Furthermore, the *d*-spacing of halogenated NGs gradually increased from **6F-HBC** to **6I-HBC** due to the increased atomic size and reduced electron negativity. The changes in the *d*-spacing on TEM analysis are in good agreement with XRD patterns (Figure S16), and the structural evaluations further confirmed that edge-substituted group 7A elements significantly tuned the structural properties of NGs. Furthermore, the TEM/EDS analysis was carried out for **6Br-HBC** at low resolution. As depicting in Figure S17, the **6Br-HBC** shows a random columnar arrangement with different size under low resolution. Moreover, the TEM/EDS mapping shows the distribution of C and Br atoms on the sample, confirming the formation of **6Br-HBC**.

**Figure 3:**
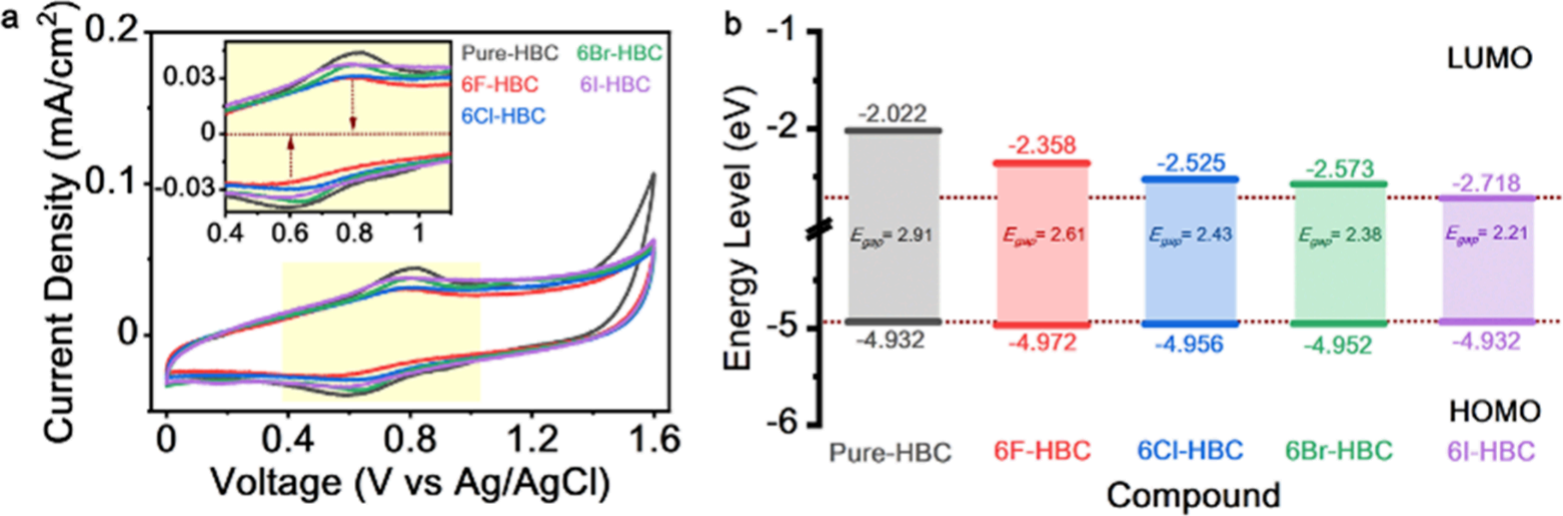
Electronic properties of all NGs. (a, b) CV spectra and energy level distribution of as-synthesized NGs, respectively.

To probe the electronic properties of NGs with edge-substituted group 7A elements, a cyclic voltammetry (CV) measurement was performed to monitor the electrochemical response of the different functional groups on the edge of NGs. As shown in Figure 3a, one reversible redox peak can be observed for all NGs at *E*_1/2_ values of 0.7 V (vs Ag/AgCl), indicating the formation of monoradical ionic species. Interestingly, the intensity of peak current density of NGs is significantly reduced in F-functionalized **6F-HBC** with a maximum charge polarization than that of **Pure-HBC**. The charge polarization in NGs was then evaluated and found to gradually increase with increasing the electronegativity of halogen atoms (F > Cl > Br > I). The HOMO–LUMO (highest occupied molecular orbital-lowest unoccupied molecular orbital) energy levels of NGs are further elucidated using CV and UV–visible spectroscopic techniques (see Supplementary Note 1 and Figures S18–S19). Figure 3b shows the distribution of HOMO–LUMO energy levels of halogenated NGs. As shown in Figure 3b, the HOMO values of NGs are slightly reduced from −4.932 eV (**Pure-HBC**) to −4.972 (**6F-HBC**) by increasing the electronegativity of halogen (I < Br < Cl < F), suggesting that the strong electron withdrawing element (F) changed the electron density and created a charge polarization on the NG basal plane (Figure 3b). Surprisingly, the energy gap (*E*_gap_) of NGs was dramatically changed by the halogen functionalization, and the trend was found to be **Pure-HBC** (2.91 eV) > **6F-HBC** (2.61 eV) > **6Cl-HBC** (2.43 eV) > **6Br-HBC** (2.38 eV) > **6I-HBC** (2.21 eV) (Figure 3b). This indicates that the charge polarization, due to the electron-withdrawing character of group 7A elements, tuned the *E*_gap_ and possibly impacted the NGs reactivity (C-halogen bonding activity). Additionally, **6I-HBC** is found to have the smallest *E*_gap_ and lowest LUMO energy level among halogenated NGs, suggesting a higher material reactivity. Importantly, the reduced *E*_gap_ and LUMO energy levels of halogenated NGs are in good agreement with the reduced electronegativity of group 7A elements (F > Cl > Br > I). This again confirms that the different charge distribution on the basal plane of halogenated NGs has a substantial influence on the materials reactivity and stability.

### Electrochemical Performance of Halogenated NGs as LIB Anode

**Figure 4:**
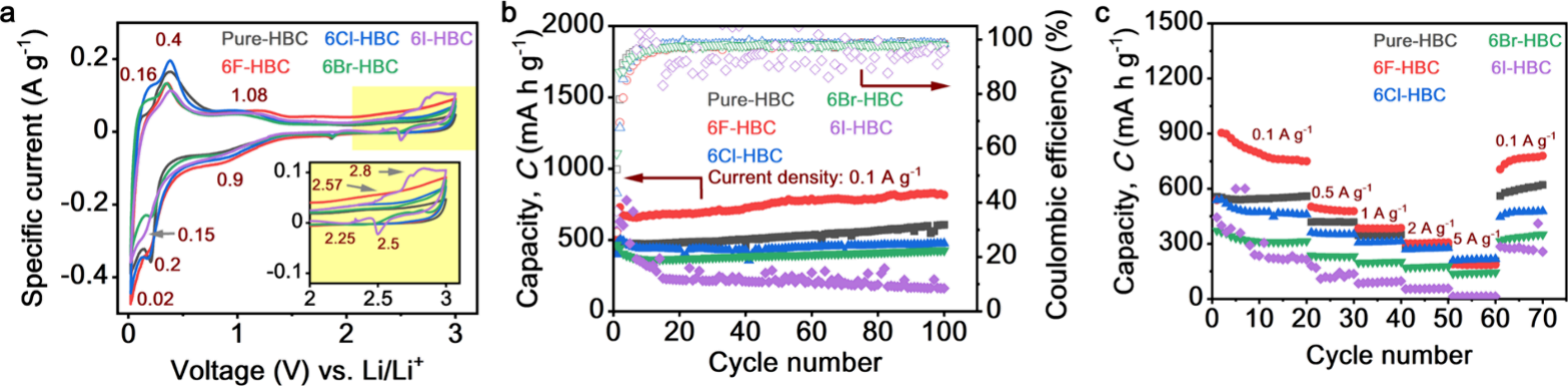
Electrochemical performances of halogenated NG anodes. (a) Cyclic voltammogram, (b) capacity profile at 0.1 A g^–1^ current density, and (c) rate performance.

The electrochemical performance of different NG assemblies was examined by fabricating NGs as anode materials in LIB half-cells. The CV was first employed in the potential window ranging between 0.02 and 3.0 V (vs Li/Li^+^) at a scan rate of 0.1 mV s^–1^ to probe the electrochemical performance and properties of edge-substituted group 7A NGs as LIB anodes. As presented in Figure 4a, three cathodic peaks at 0.9, 0.15–0.21, and 0.02 V can be observed for all NGs, which indicate the typical Li^+^ adsorption and intercalation in the carbonaceous materials.^9,15,46^ Furthermore, these peaks are along with three peaks on the anodic scan at around 0.16, 0.4, and 1.08 V, reflecting as Li^+^ extraction and deintercalation process. Importantly, **6I-HBC** showed an additional specific feature at potential of 2–3 V, where two cathodic peaks can be monitored at 2.25 and 2.5 V, along with two anodic peaks at 2.57 and 2.8 V (inset Figure 4a). These phenomena can be ascribed due to the reactivity of **6I-HBC** toward the oxidation reaction at higher voltage, thus possibly the release of I^–^ from the edge of NGs during the electrochemical process then reacted with Li^+^ subsequently forming Li-halogen compounds.

The galvanostatic profile of NG anodes (Figure S20) is in good agreement with the CV (Figure 4a) during the electrochemical charge/discharge process. As shown in Figure S20, the significant capacity loss can be observed for all NGs at the first charge/discharge cycle at 0.1 A g^–1^. This significant capacity loss could have originated from the reduction of the electrolyte, thus resulting in the formation of a solid electrolyte interphase (SEI).^47−49^ After 100 cyclic charge–discharge processes, all of the NGs exhibited a reversible capacity, indicating their excellent Li^+^ storage capability. Importantly, **6I-HBC** exhibits a severe capacity reduction from first cycle to 100th than other NGs (Figure S20), which could be due to its higher reactivity thus resulting in the detachment of I^–^ and formation of Li–I compounds during charge/discharge process. This phenomenon is consistent with the redox couples between 2–3 V during CV test (Figure 4a) and energy level distribution (Figure 3b).

Figure 4b shows the cycling performance of halogenated NG anodes at 0.1 A g^–1^ for up to 100 cycles. As shown in Figure 4b, **6F-HBC** exhibits superior capacity of 816 mA h g^–1^ among other NGs with excellent Coulombic efficiency (CE) of ∼98%. This specific discharge capacity is followed by **Pure-HBC**, **6Cl-HBC**, **6Br-HBC**, and **6I-HBC** with 606, 477, 427, and 162 mA h g^–1^, respectively. The cycling performance of **6F-HBC** is among the highest specific capacity of polycyclic aromatic hydrocarbons ever reported (Figure S21). Additionally, a slurry consisting of 80% conductive carbon (Super P) and 20% binder (PVDF), **SP80**, has been prepared and measured to probe the capacity contribution from conductive carbon. Interestingly, this capacity contributed from the conductive carbon was significantly low as compared with halogenated NG anodes (Figure S22). Moreover, this capacity trend is significantly different compared with the theoretical capacity of halogenated NGs (see Supplementary Note 2). The remarkable capacity of **6F-HBC** could be associated with the F functionalization on the edge of NG which tuned its structural and electronic properties. A previous study has been reported that the maximum charge polarization on the basal plane of halogen-terminated NGs could improve and stabilize Li^+^ adsorption in functionalized graphene materials.^50^ Furthermore, a calculation study also revealed that the maximum charge polarization due to halogen functionalization on the graphene edge could provide a confinement effect in which metal ions are trapped in the bulk region of F graphene.^51^ This confinement effect can also be seen on the XRD pattern (Figure S16a) and TEM analysis (Figure 2h) for **6F-HBC** with a lower interlayer and *d*-spacing than that of **Pure-HBC** (3.43 to 3.31 Å). This reduced interlayer spacing and high electron density on the NG edge due to F functionalization could create a confinement for Li^+^ to be trapped in the interlayer of **6F-HBC**. Meanwhile, **Pure-HBC**, without halogen functionalization, exhibited the second highest specific capacity than that of **6Cl-HBC**, **6Br-HBC**, and **6I-HBC** (Figure 4b). This phenomenon could be associated with the stacking behavior of NGs. As shown in Figure S16, **6Cl-HBC**, **6Br-HBC**, and **6I-HBC** showed an out-of-planar stacking orientation when compared to **Pure-HBC** and **6F-HBC**, which is attributed to the increasing atomic radius as reducing the electronegativity of Cl, Br, and I atoms on the edge of NGs. This out-of-planarity structure possibly drives **6Cl-HBC**, **6Br-HBC**, and **6I-HBC** with minimum Li^+^ storage capability. Therefore, it implies that a planar stacking orientation is favorable for Li^+^ insertion. In addition, slow capacity fading can be observed for **6I-HBC** along with scattered CE at 0.1 A g^–1^ (Figure 4b), which is consistent with the galvanostatic profile (Figure S20) and further indicates the material instability during charge/discharge process. In addition, **6F-HBC** shows a remarkable rate capability by delivering specific capacity of 780, 490, 380, 301, and 186 mA h g^–1^ at current density of 0.1, 0.5, 1, 2, and 5 A g^–1^, respectively (Figure 4c). The reduced specific capacity under high current density is ascribed to the rapid Li^+^ insertion/extraction process under extreme current densities. Moreover, the excellent rate capability of **6F-HBC** with the highest capacity of 780 mA h g^–1^ can be recovered when the current density is turned back to 0.1 A g^–1^, indicating a great material stability under extreme charge/discharge rates. Moreover, the long-term working cycle indicated that **6F-HBC** has superior capacity of 640 mA h g^–1^ than other halogenated NGs for up to 500 cycles at 1 A g^–1^ current density (see Figure S23; red line). Furthermore, the specific capacities of **Pure-HBC**, **6Cl-HBC**, and **6Br-HBC** were found to be 520, 350, and 250 mA h g^–1^, respectively, after 500 cycles at 1 A g^–1^.

Notably, **6I-HBC** again showed a gradually capacity decay during long-term operation for up 48 mA h g^–1^ after 500 cycles at 1 A g^–1^ (Figure S23; purple line), indicating poor cycling performance. This poor cycling performance of **6I-HBC** could be ascribed due to its reactivity with Li-ion, which is in good agreement with the energy level distribution (Figure 3b), CV (Figure 4a), and galvanostatic profile (Figure S20). In brief summary, the edge-substituted group 7A elements have significant impact on the electrochemical performance of NGs as LIB anodes. The higher electronegative element (F) can create a strong charge polarization on basal planes of NGs for stabilizing Li^+^ absorption with a confinement effect on the 2D NG structure, thus significantly improving the Li^+^ storage capability.^50,51^ Vice versa, the lowest electronegative I creates a low charge polarization and results in the material instability and capacity decay during charge/discharge process because of the detachment of iodine functionalization and formation of Li–I compounds.

### Mechanistic Study of Halogenated NGs as LIB Anodes

*Ex situ* XPS was employed at various charge/discharge stages to evaluate the Li^+^ storage mechanism in the halogenated NGs (see Supplementary Note 3 and Figures S24–S28). It is confirmed that a successful Li^+^ storage mechanism in the halogenated NGs is contributed from the formation of LiC_6_ and additional adsorption from the halogen atom during lithiation process. The increasing Li^+^ adsorption and material stability is confirmed for **6F-HBC** leading to an outstanding specific capacity. Notably, a distinct chemical behavior is observed for **6I-HBC**. The detachment of I^–^ from the HBC structure, and later consumed during the electrochemical process, is confirmed from the *ex situ* XPS study. This phenomenon is believed to be resulted from a lower LUMO energy and *E*_gap_, thereby increasing its reactivity.

To probe the transport properties of halogenated NGs, electrochemical impedance spectroscopy (EIS) was employed in the frequency range from 10 mHz to 1 MHz with an AC amplitude of 10 mV at various cycling intervals. The Nyquist and equivalent circuits models used to fit the impedance data are presented in Figure S29. Furthermore, in terms of the basic kinetic reaction during the electrochemical process, the Li^+^ diffusion coefficient (*D*_Li_) can be estimated based on the typical Warburg impedance element in the low-frequency region (see Supplementary Note 4). This is indicated by a typical spike line with a slope of ∼45° in the Nyquist plot.^52^ All fitted parameters are summarized in Table S1. As shown in Figure S29 and Table S1, two charge transfer resistances can be observed at two different interfaces, namely, solid electrolyte interfaces (SEI; *R*ct_a_) and electrode interface (*R*ct_b_). As presented in Table S1, the *R*ct_b_ of **Pure-HBC**, **6F-HBC**, and **6Cl-HBC** is significantly reduced after 100 cycles scans, along with an increase in Li^+^ diffusion coefficient, indicating an excellent Li^+^ transport property. Although **6Cl-HBC** was found to have lower *R*ct_a_ and *R*ct_b_, as well as faster diffusion coefficient, it also exhibited lower Li^+^ storage capability than **Pure-HBC** and **6F-HBC** (Figure 4b). This phenomenon possibly results from the materials stacking properties. As shown in Figure S16, **6Cl-HBC** exhibits an out-of-planar stacking orientation when compared to **Pure-HBC** and **6F-HBC**, which is attributed to the increasing atomic radius and reducing the electronegativity of Cl atom on the edge of NGs. This out-of-planarity structure possibly drives the **6Cl-HBC** to have faster Li^+^ transport from adsorption behavior; however, it minimizes Li^+^ storage capability. Interestingly, **6I-HBC** has a significant formation of *R*ct_a_ (1140.92 Ω) after 100 cycles scan (Table S1), which by far is the highest value of *R*ct_a_ among the NGs. This highest *R*ct_a_ of **6I-HBC** further maintains the *D*_Li_ at ∼10^–14^ after 100 cycles, suggesting hindrance to Li^+^ due to highly resistive SEI. This phenomenon could be associated with the increased materials reactivity toward the oxidation reaction, leading to the release of iodo-functionalization and the formation of more resistive species in the SEI layer, consequently impeding Li^+^ transport. This is consistent with the observed capacity decay in the cycling performance (Figure 4b).

**Figure 5:**
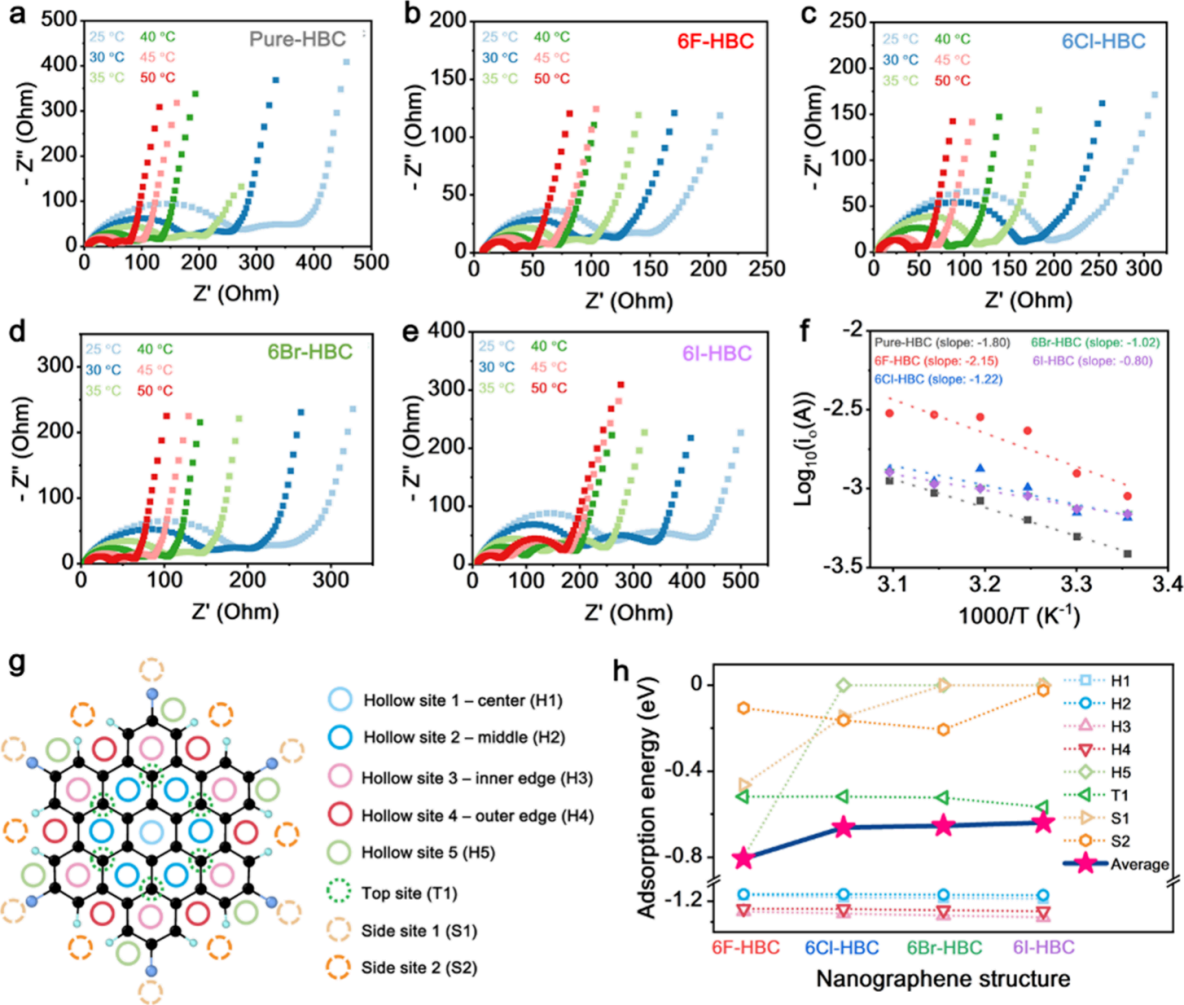
Mechanistic studies of NGs. (a–e) are the Nyquist plots of **Pure-HBC**, **6F-HBC**, **6Cl-HBC**, **6Br-HBC**, and **6I-HBC**, respectively, at different temperatures for Ea analysis. (f) Arrhenius plots of log *i*_0_ versus 1/T for NGs. The dot lines are the linear fitting results. (g) Identified potential Li^+^ adsorption sites. (h) Li^+^ adsorption energy at various sites.

The apparent activation energies of halogenated NGs as anode LIBs were further investigated to understand the charge transfer energy of the NGs. The exchange current (*i*_0_) and activation energy (*E*_a_) can be estimated from the Arrhenius Equation:^9,53^*i*_0_ = *RT*/*nFR*ct and *i*_0_ = *A* exp(−*E*_a_/*RT*), where *A* is a temperature-independent coefficient, *R* is the gas constant, *T* (K) is the absolute temperature, *n* is the number of transferred electrons, *F* is Faraday constant and *E*_a_ is apparent activation energy. A study of the activation energies is useful to clearly manifest the charge transfer reaction at NGs, which can be further estimated the material’s reactivity and stability during electrochemical reaction.^54^ EIS analysis was performed to obtained the charge transfer resistant at the NG’s interfaces at different temperature. Prior to the EIS analysis, the NGs were charged at 0.1 A g^–1^ up to a cutoff voltage of 1 V after its first cycle to minimize the interference from electrolyte decomposition at low voltages. As shown in Figure 5a–e, two semicircles can be observed for all NGs at different temperatures. The first semicircle can be attributed to the charge transfer from electrolyte to the electrode, while the second can be ascribed to the charge transfer at the material’s interface in the electrode. To evaluate the material’s kinetics, the charge transfer ascribed from the second semicircle was used. The apparent activation energies can be estimated from *E*_a_ = −*Rk*ln10, where *k* = the slope of the fitting line of the Arrhenius plots (Log10i_0_ as a function of 1000/T) (Figure 5f). The *E*_a_ of NGs were estimated to be 34.49, 40.68, 23.52, 19.46, and 15.84 kJ mol^–1^ for **Pure-HBC**, **6F-HBC**, **6Cl-HBC**, **6Br-HBC**, and **6I-HBC**, respectively. These results show that **6F-HBC** requires higher energy to promote a charge transfer reaction than other NGs, indicating a higher material stability, which possibly contributes to the improvement of cycling stability and Li^+^ storage capability. On the other hand, **6I-HBC** exhibits the lowest *E*_a_, suggesting higher material reactivity toward Li^+^ during electrochemical process, which is consistent with the *E*_gap_ analysis (Figure 3b). The higher reactivity in **6I-HBC** toward Li^+^ could potentially initiate side reactions through the detachment of I functionalization and then reacting with Li^+^ to form Li–I species, resulting in lower cycling stability. Such parasitic reactions can also be observed on CV at higher potential (Figure 4a). Furthermore, this reaction could also be the reason for the lower Li^+^ storage capability in **6I-HBC** compared to other NGs (Figure 4b). The Li–I resistive species could create an additional barrier for Li^+^ mobility, significantly dropping the battery performance, which is consistent with the EIS data. Importantly, the *E*_a_ analysis is in good agreement with the *E*_gap_ calculation (Figure 3b), where the *E*_gap_ reduces along with the decreasing electronegativity of halogens (F > Cl > Br > I). This again confirms that group 7A elements not only tune intrinsic electronic properties of NGs, but also impact the charge transfer energy during electrochemical reactions.

To probe the charge storage behavior of halogenated NGs, the sweep rate CV analysis at different scan rates (Supplementary Note 5 and Figure S31) was employed to monitor the consecutive electrochemical reactions occurring in the NG anodes. As shown in Figure S32, the *b* value of NGs, which was obtained from the slope of the log *i* against the log *v*, are estimated to be 0.589, 0.7471, 0.9409, 0.8596, and 0.6826 for **Pure-HBC**, **6F-HBC**, **6Cl-HBC**, **6Br-HBC**, and **6I-HBC**, respectively. Interestingly, **6Cl-HBC** and **6Br-HBC** have higher *b* values than other NGs, indicating that the electrochemical process was subjected to a surface-controlled process (capacitive effects). This suggests that the out-of-planar structure of halogenated NGs drives the charge storage electrochemical process to have more capacitive effects. Furthermore, the improved Li-ion absorptivity in **6F-HBC** can be observed as a slightly increase in the *b* value (0.7471; Figure S32b) compared to **Pure-HBC** (0.589; Figure S32a) on the planar structure. This indicates that the synergetic contribution of capacitive and diffusion behavior plays an important role in charge storage behavior of **6F-HBC**, resulting in superior Li^+^ storage among halogenated NGs.

Additionally, a quantitative analysis based on the stored charge can provide a more in-depth information associated with the capacitive and diffusion contributions (see Supplementary Note 5). The total charge stored for the reactions of capacitive (*k*_1_*v*) and diffusion (*k*_2_*v*^1/2^) can be estimated as the slope and the intercept from the *i*/*v*^1/2^ against *v*^1/2^ plot (Figure S33). Interestingly, the charge storage behavior in **6Cl-HBC** and **6Br-HBC** is found to be dominated by a surface-controlled process (capacitive effect) at various scan rates (Figure S34c and d), which is in good agreement with the *b* value (Figure S32c and d). Meanwhile, **6F-HBC** is found to have capacitive contributions approximately 50% at a high scan rate (1–0.5 mV s^–1^), and this number then gradually decreased up to 13% at 0.1 mV s^–1^, resulting in a substantial diffusion contribution enhancement of up to 87% (Figure S34b). This again confirms that the Li-ion uptake in **6F-HBC** is governed by synergic contributions from capacitive and diffusion-controlled processes at higher scan rates (1–0.5 mV s^–1^). Meanwhile, when the scan rate was reduced to 0.1 mV s^–1^, the diffusion-controlled process starts to play the key role on total charge stored in the **6F-HBC** structure (confinement effect). In summary, the chemistry of NGs as anode LIBs is strongly affected by the halogen functionalization. The material reactivity and stability as well as structural alteration of NGs, resulting from edge-substituted halogens, have an impact not only on the transport properties, but also on the charge transfer energy and the charge storage behavior. The strong charge polarization due to high electronegativity functionalization (F; 4.0) could increase the stability of NGs and reduce their reactivity as LIB anode materials by increasing *E*_a_. Conversely, the low electronegativity functionalization (I; 2.5) significantly reduces *E*_a_ for NGs anodes, thus dramatically increasing their reactivity, resulting in poor material stability. Furthermore, the structural deformation from planar to out-of-planar due to halogen functionalization, such as Cl and Br, also significantly impacts the transport properties and charge storage behavior.

To verify the effect of halogenation to the NGs structure, DFT calculations have been performed with various structures (see Supplementary Note 6 and Figures S35–S40). The DFT calculations confirmed that the planar conformation of **6F-HBC** is preferential over its nonplanar counterpart because the planar conformation yields lower potential energy relative to the nonplanar one (Figure S40). This phenomenon could be ascribed to the strongest hydrogen bond and shortest C-halogen bond of **6F-HBC**, as well as the lowest ground state energy (Figure S41). Meanwhile, **6Cl-HBC**, **6Br-HBC**, and **6I-HBC** yield much lower energy differences between their planar and nonplanar conformations.

Furthermore, a series of Li adsorption DFT calculations have been performed to probe the active adsorption sites for Li^+^ adsorption in the halogenated NGs (see Supplementary Note 6 and Figure 5g). As shown in Figure 5h, Table S2, and Figure S43, it is clear that the hollow sites have higher stability for Li^+^ adsorption in all 2D halogenated NGs with the adsorption energies ranging around −1.1 to −1.3 eV. Interestingly, additional stable Li^+^ adsorption sites at H5 and S1 can be identified for **6F-HBC** with the adsorption energies of −0.7990 and −0.4636 eV, respectively, indicating additional active adsorption sites for Li^+^ storage in **6F-HBC** (Figure 5h and Figure S43). As a result, **6F-HBC** has shown to give a lower average adsorption energy of −0.8063 eV than those of **6Cl-HBC** (−0.6617 eV), **6Br-HBC** (−0.6534 eV), and **6I-HBC** (−0.6386 eV) (Figure 5h and Figure S44), implying the reason why **6F-HBC** yields the highest capacity compared with other halogenated NGs.

The additional adsorption sites at the edge (H5 and S1 sites in Figure 5h) of **6F-HBC** can be attributed to the strong interactions between fluorine and lithium. In contrast, for **6Cl-HBC** and **6Br-HBC**, adsorption energies at these sites are close to zero due to intermediate interaction with halogen atoms as well as weaker H-bond interactions. As a result, during DFT relaxation, Li will quickly relax to the hollow sites close to the interior of the NGs (e.g., H3 sites in Figure 5h). Importantly, we found that **6I-HBC** decomposes at the presence of Li^+^ due to hydrogen bond breaking even during the initial insertion (Figure S45d and Supplementary Movie 4). Subsequently charge density analysis of the halogenated NGs suggested the charge density is truncated between halogen and carbon atoms in **6I-HBC** for both the 2D plot and 3D 0.3 e/bohr^3^ isosurfaces (Figure S46d), indicating weaker C–I bonds. Hence, **6I-HBC** NG is unstable even without the presence of Li^+^, resulting in the lowest capacity as LIB anode. Hence, our theoretical calculations revealed that more available adsorption sites at the outskirt of the NGs is the primary reason for the superior capacity of **6F-HBC** over other halogenized NGs.

## Conclusion

In summary, this work demonstrates a systematic study on the edge-substituted group 7A elements in NGs for LIB anodes. We found that group 7A elements could regulate the structural and electronic properties of NGs, thus significantly tuning its chemistry as LIB anode materials. Kinetic studies reveal that the group 7A elements have also regulated the charge transport properties of NG anodes. Interestingly, the out-of-planar structures of **6Cl-HBC** and **6Br-HBC** accelerate Li^+^ transport properties and increase capacitive behavior, but this event does not improve Li^+^ storage capability due to lack of structural orientation. Meanwhile, the planar structure with strong polarization from the high electronegativity group 7A element, fluorine, provides the highest Li^+^ storage capability due to the synergic contribution from the high material stability and special structural property (confinement effect). Furthermore, the charge transfer energy of halogenated NGs is in good agreement with the reduction of electronegativity group 7A elements (F > Cl > Br > I). This study provides a deep understanding of NG-based anodes starting from material design to electrochemical performance, which will be beneficial for the development of next-generation graphene-based energy storage devices.

## Experimental Section

### Materials

All chemicals, unless otherwise specified, were purchased from commercial resources and used as received without further purification. The detail materials synthesis is presented below:

#### Hexaphenylbenzene (**6H-HPB**)

A mixture of diphenylacetylene (4.34 g, 24.11 mmol) and tetraphenylcyclopentadienone (7.77 g, 20.02 mmol) in Ph_2_O (6 mL) was purged with N_2_ and heated at reflux for 24 h. The mixture was cooled, acetone (200 mL) was then added, and the resulting solid was separated by filtration to afford **6H-HPB** as a white powder (10.31 g, 96.31%).

#### 1,2-Bis(4-fluorophenyl)acetylene (F-ac-F)

1-Fluoro-4-iodobenzene (20.90 g, 94.59 mmol), Pd(PPh_3_)_4_ (1.64 g, 1.42 mmol), CuI (1.52 g, 8.0 mmol), 1,8-diazabicyclo[5.4.0]undec-7-ene (65 mL), water (0.5 mL), and toluene (200 mL) were mixed together under argon atmosphere. Then, trimethylsilylacetylene (6.3 mL, 50.67 mmol) was added slowly. The reaction mixture was stirred overnight at 60 °C. After cooling, the product was filtered to afford a white solid (9.75 g, 96.71%). ^1^H NMR (CDCl_3_, δ, ppm): 7.48–7.52 (m, 4H), 7.02–7.07 (m, 4H).

#### 1,2-Bis(4-chlorophenyl)acetylene (Cl-ac-Cl)

1-Chloro-4-iodobenzene (25.0 g, 104.84 mmol), Pd(PPh_3_)_4_ (2.04 g, 1.77 mmol), CuI (1.68 g, 8.85 mmol), 1,8-diazabicyclo[5.4.0]undec-7-ene (95 mL), water (0.75 mL), and toluene (150 mL) were mixed together under an argon atmosphere. Then, trimethylsilylacetylene (7.0 mL, 50.53 mmol) was added slowly. The reaction mixture was stirred overnight at 60 °C. After cooling, the product was filtered to afford a pale brown solid (8.95 g, 69.75%). ^1^H NMR (CDCl_3_, δ, ppm): 7.45 (d, 4H), 7.33 (d, 4H).

#### 1,2-Bis(4-bromophenyl)acetylene (Br-ac-Br)

1-Bromo-4-iodobenzene (20.07 g, 70.69 mmol), Pd(PPh_3_)_4_ (1.62 g, 1.41 mmol), CuI (1.35 g, 7.08 mmol), 1,8-diazabicyclo[5.4.0]undec-7-ene (64 mL), water (0.48 mL), and toluene (200 mL) were mixed together under argon atmosphere. Then, trimethylsilylacetylene (4.5 mL, 32.48 mmol) was added slowly. The reaction mixture was stirred overnight at room temperature. After cooling, the product was filtered to afford a white solid (7.72 g, 64.95%). ^1^H NMR (CDCl_3_, δ, ppm): 7.49 (d, 4H), 7.38 (d, 4H).

#### Hexakis(4-fluorophenyl)benzene (**6F-HPB**)

F-ac-F (0.51 g, 2.37 mmol) and dicobalt octacarbonyl (0.81 g, 0.24 mmol) were heated at reflux temperature in dioxane (10 mL) for 24 h. The solvent was reduced in vacuo, dissolved in dichloromethane, and filtered through Celite to remove cobalt particles. The products were washed by methanol and filtered to afford a white solid (0.33 g, 64.55%). ^1^H NMR (CDCl_3_, δ, ppm): 6.71–6.74 (m, 12H), 6.59–6.63 (m, 12H). ^13^C NMR (CDCl_3_, δ, ppm): 161.89, 159.93, 140.09, 136.10, 136.07, 132.72, 132.65, 114.33, 114.16.

#### Hexakis(4-chlorophenyl)benzene (**6Cl-HPB**)

Cl-ac-Cl (1.0 g, 4.05 mmol) and dicobalt octacarbonyl (0.14 g, 0.43 mmol) were heated at reflux temperature in dioxane (10 mL) for 1 day. After cooling, the product was filtered and washed by THF to afford a pale brown solid (0.67 g, 66.49%). ^1^H NMR (CDCl_3_, δ, ppm): 6.90 (d, 12H), 6.68 (d, 12H). ^13^C NMR (CDCl_3_, δ, ppm): 139.82, 138.18, 132.39, 132.18, 127.64.

#### Hexakis(4-bromophenyl)benzene (**6Br-HPB**)

Br-ac-Br (1.02 g, 3.02 mmol) and dicobalt octacarbonyl (0.10 g, 0.30 mmol) were heated at reflux temperature in dioxane (10 mL) for 24 h. After cooling, the product was filtered and washed by dichloromethane to afford a white solid (0.63 g, 62.94%). ^1^H NMR (CDCl_3_, δ, ppm): 7.06 (d, 12H), 6.61 (d, 12H).

#### Hexakis(4-iodophenyl)benzene (**6I-HPB**)

The mixture of **6H-HPB** (2.0 g, 3.73 mmol), bis(trifluoroacetoxy)iodobenzene (5.92 g, 13.34 mmol), and iodine (3.37 g, 13.24 mmol) in dry dichloromethane (130 mL) under nitrogen was stirred at room temperature for 24 h in the dark, and then, hexane was added to the reaction mixture. The resulting precipitate was filtered and washed with hexane. This solid was dissolved in chloroform and washed with Na_2_S_2_O_4_ aqueous solution and saturated NaCl aqueous solution. The organic layer was dried over anhydrous Na_2_SO_4_ and concentrated under reduce pressure. The residue was recrystallized from chloroform-hexane to give a white solid (3.65 g, 75.66%).

#### Hexa-*peri*-hexabenzocoronene (**Pure-HBC**)

Compound **6H-HPB** (0.5 g, 0.94 μmol) was dispersed in dry dichloromethane (30 mL). The solution was degassed via bubbling nitrogen for 30 min. Then, FeCl_3_ (6.37 g, 38.53 mmol) in dry nitromethane (30 mL) was added slowly via a syringe. After 3 h, the reaction was quenched with a large volume of methanol. The dark precipitate was collected and washed with methanol and water. The final brown precipitate was then collected and dried in a vacuum to afford nanographene **6H-HBC** (0.38 g, 77.0%). MALDI-TOF MS: calcd for (C_42_H_18_)^+^: *m*/*z* 522.1409; found: *m*/*z* 522.1411.

#### Hexakis(4-fluoro)-*peri*-hexabenzocoronene (**6F-HBC**)

Compound **6F-HPB** (1.0 g, 1.56 μmol) was dispersed in dry dichloromethane (50 mL). The solution was degassed via bubbling nitrogen for 30 min. Then, FeCl_3_ (9.80 g, 56.06 mmol) in dry nitromethane (20 mL) was added slowly via a syringe. After 12 h, the reaction was quenched with a large volume of methanol. The dark precipitate was collected and washed with methanol and water. The final brown precipitate was then collected and dried in a vacuum to afford nanographene **6F-HBC** (0.67 g, 68.52%). ^13^C{^1^H} CP/MAS NMR (δ, ppm): 160.04/158.45, 128.86, 117.52, 114.15, 108.40. ^19^F MAS NMR (δ, ppm): −114.35. MALDI-TOF MS: calcd for (C_42_H_12_F_6_)^+^: *m*/*z* 630.0843; found: *m*/*z* 630.0855.

#### Hexakis(4-chloro)-*peri*-hexabenzocoronene (**6Cl-HBC**)

To a mixture of copper(II)-trifluoromethanesulfonate (8.46 g, 23.38 mmol) and aluminum(III) chloride (3.06 g, 22.93 mmol) in carbon disulfide (100 mL), **6Cl-HPB** (0.50 g, 0.67 mmol) was added. After stirring at room temperature for 24 h under an nitrogen atmosphere, the resulting mixture was poured into methanol. The yellowish precipitate was collected and washed with dichloromethane and water. The final yellow precipitate was then collected and dried in a vacuum to afford nanographene **6Cl-HBC** (0.44 g, 66.22%). ^13^C{^1^H} CP/MAS NMR (δ, ppm): 140.44, 138.48, 133.64/132.96, 132.27, 128.67. MALDI-TOF MS: calcd for (C_42_H_12_Cl_6_)^+^: *m*/*z* 725.9070; found: *m*/*z* 725.9077.

#### Hexakis(4-bromo)-*peri*-hexabenzocoronene (**6Br-HBC**)

To a solution of **6Br-HPB** (1.0 g, 1.0 mmol) in dry dichloromethane (100 mL) was added 2,3-dichloro-5,6-dicyanobenzoquinone (DDQ, 1.63 g, 7.18 mmol) at 0 °C. After the solution was degassed via bubbling nitrogen for 30 min, trifluoromethanesulfonic acid (1.2 mL) was added to the mixture. The mixture was further stirred at 0 °C overnight in the dark, and then, methanol was added to the reaction mixture. A brown precipitate was collected and washed with dichloromethane and water. The final yellowish brown precipitate was then collected and dried in a vacuum to afford nanographene **6Br-HBC** (0.89 g, 89.23%). MALDI-TOF MS: calcd for (C_42_H_12_Br_6_)^+^: *m*/*z* 995.9804; found: *m*/*z* 995.9810.

#### Hexakis(4-iodo)-*peri*-hexabenzocoronene (6I-HBC)

Compound **6I-HPB** (1.0 g, 0.77 μmol) was dispersed in dry dichloromethane (200 mL). The solution was degassed via bubbling nitrogen for 10 min. Then, FeCl_3_ (3.02 g, 18.27 mmol) in dry nitromethane (5 mL) was added slowly via a syringe. After 12 h, the reaction was quenched with a large volume of methanol. The dark precipitate was collected and washed with methanol. The final yellowish brown precipitate was then collected and dried in a vacuum to afford nanographene **6I-HBC** (0.79 g, 80.44%). MALDI-TOF MS: calcd for (C_42_H_12_I_6_)^+^: *m*/*z* 1277.5201; found: *m*/*z* 1277.5094.

#### Material Characterization

^1^H and ^13^C nuclear magnetic resonance (NMR) spectra were measured on a Bruker AVANCE-500 FT-NMR spectrometer (Billerica, MA, US), operating at frequencies of 500 MHz for ^1^H and 125 MHz for ^13^C measurements with CDCl_3_ as solvent. All measurements were carried out at standard conditions at room temperature. Chemical shifts are reported in parts per million (ppm, δ) relative to the solvent residual proton (CDCl_3_, δ7.26) and carbon (CDCl_3_, δ77.2) signals. Peak multiplicity was reported as follows: s, singlet; d, doublet; t, triplet; and m, multiplet. Molecular weights were obtained using a matrix-assisted laser desorption/ionization time-of-flight (MALDI-TOF; Bruker, New ultrafleXtremeTM, Bremen, D.E.) mass spectrometry. The ^1^H/^13^C cross-polarization/magic angle spinning (CP/MAS) NMR and ^19^F MAS NMR spectra were acquired on a Bruker Avance 300 MHz spectrometer (Bruker Spectrospin, Rheinstetten, Germany) equipped with a 4 mm double resonance probe operating at ^1^H, ^19^F, and ^13^C Larmor frequencies of 300.13, 282.40, and 75.47 MHz, respectively. For effective ^1^H/^13^C cross-polarization, contact-time set to 1 ms and radio frequency (rf) of 37.0 kHz were selected for both the ^1^H and ^13^C channels to fulfill Hartmann–Hahn matching condition.^55^ During data acquisition, ^1^H decoupling by two-pulse phase modulation^56^ was applied, with an rf field strength of 72 kHz. The ^19^F MAS NMR spectra were recorded with a single pulse excitation of rf field strength of 53 kHz. All the powdered sample was packed into a 4 mm zirconia MAS rotor, and the ^13^C (^19^F) measurements were conducted at ambient temperature with a recycle delay of 4 s and a MAS rate of 5 kHz (9 kHz) regulated by a spinning controller to within ∼1 Hz. The ^13^C and ^19^F chemical shifts were referenced to the glycine carboxyl carbon signal at 176.4 ppm, and the 4-fluorobenzoaldehye signal at −102.38 ppm, respectively. Fourier transform infrared spectroscopy (FT-IR) were measured using attenuated total reflectance (ATR) mode on an FT/IR 6600, JASCO International Co., Ltd. Thermogravimetric analysis (TGA) was conducted with a PerkinElmer Pyris 1 TGA. Experiments were carried out on approximately 5 mg film samples heated in flowing nitrogen (flow rate = 20 cm^3^/min) at a heating rate of 20 °C/min. The X-ray diffraction patterns were measured on a Bruker D8 Advance X-ray diffractometer at 40 kV and 40 mA using Cu Kα radiation (λ = 1.5406 Å). X-ray photoelectron spectroscopy (XPS) was measured with ULVAC-PHI, Quantes, using a monochromatic Al K(alpha) source (∼1.5 keV) and equipped with a microfocused electron gun. A transmission electron microscope (TEM, FEI Tecnai G2 F20 S-TWIN) was used to perform the TEM analysis. The TEM/EDS was measured using JEOL JEM-ARM300F2 with the accelerating voltage of 80 kV. The electronics properties of NGs were evaluate using CV measurement in a bipotentiostat (CHI 760e) using a three-electrode setup with the glassy carbon electrode (GCE), Ag/AgCl, and a platinum (Pt) wire as the working electrode, reference electrode, and counter electrode, respectively. All UV–vis spectroscopy measurements were carried out in a spectrometer (Agilent Technologies Cary 8454 UV–vis).

#### Coin Cell Preparation and Electrochemical Measurement

The anode was prepared by mixing 40 wt % NGs, 40 wt % conductive carbon (Super P), 20 wt % poly(vinylidene fluoride) (PVDF) binder, and *N*-methyl-2-pyrrolidone solvent to form a homogeneous slurry mixture. The mixture was casted onto Cu foil and dried on a hot plate at 60 °C for overnight (12h) then continued in the vacuum oven at 80 °C for another 8 h. The electrodes were cut 12 mm in diameter with average loading of 0.35 mg cm^–2^ then transferred inside of the glovebox for coin cell fabrication. Additionally, the slurry consists of 80 wt % conductive carbon (Super P) and 20 wt % PVDF binder, **SP80**, and has also been prepared using a similar method. Finally, CR2032 type coin cells were assembled in a high-purity argon-filled glovebox (H_2_O < 0.5 ppm, O_2_ < 0.5 ppm, Vigor, Vigor tech USA) using as prepared anode as the working electrode, Li metal foil as a counter/reference electrode, Celgard 2325 as the separator, and 40 μL of 1 M of LiPF_6_ in a 1:1 (v/v) ethylene carbonate/diethyl carbonate (EC/DEC) as the electrolyte. The cyclic voltammetry was performed using a MultiPalmSens4 electrochemical analyzer, PalmSens BV, at a scan rate of 0.1 mV s^–1^ between 0.02 and 3.0 V. The EIS analysis were conducted before and after battery cycle using CHI electrochemical workstation model 760e, CH Instruments, Inc., with an alternating current (AC) voltage signal of 10 mV and frequency range between 10 mHz and 1 MHz. The cells were charged and discharged galvanostatically using AcuTech battery station systems (AcuTech Systems Co. Ltd.). Charge–discharge studies of the coin cells were performed by using a programmable battery tester in constant current mode in the potential range of 0.02–3.0 V.

#### Density Functional Theory (DFT) Simulations

All calculations were performed using density functional theory (DFT)^57,58^ as implemented in Vienna *ab initio* Simulation Package (VASP)^59^ 6.3.2 utilizing projector-augmented waves (PAW)^60^ potentials. The generalized gradient approximation (GGA) proposed by Perdew–Burke–Ernzerhof (PBE)^61^ was incorporated to account for the exchange-correlation energy. The vdW interactions were included by employing the Becke–Johnson (BJ)^62^ damping implemented in the DFT-D3 correction method of Grimme et al.^63^ All energy calculations including different halogen-decorated nanographene flakes were carried out in a large simulation cell with constant volume and 15 Å of vacuum added in all directions (a, b, and c) from the Li-flake system to minimize the influence of atoms across the cell boundaries. Therefore, all obtained values are from single gamma point calculations. The plane-wave energy cut off value was set to 400 eV, while all the structures were relaxed with the convergence criteria set to 10^–5^ eV and 1 meV/Å^–1^ for energy and atomic forces, respectively.

## Supporting Information Available

Supplementary notes, NMR spectra (Figures S1–S8), MALDI-TOF MS spectra (Figures S9–S12), FTIR spectra (Figure S13), TGA thermograms (Figure S14), XPS spectra (Figures S15, S24–S28), XRD diffractograms (Figure S16), TEM/EDS images (Figure S17), CV curve (Figure S18), UV–visible spectra (Figure S19), galvanostatic profile (Figure S20), performance comparison (Figures S21–S22), long cycling performance (Figure S23), Nyquist plots (Figure S29), Warburg impedance (Figure S30), sweep rate analysis (Figures S31–S34), DFT simulations (Figures S35–S46), Supplementary Tables (Tables S1–S2). (PDF)
Li^+^ adsorption pathway (Supplementary Movie 1) (MP4)
Li^+^ adsorption pathway (Supplementary Movie 2) (MP4)
Li^+^ adsorption pathway (Supplementary Movie 3) (MP4)
Li^+^ adsorption pathway (Supplementary Movie 4) (MP4)
