# Supplementary material for: Lithium-Ion Dynamic and Storage of Atomically Precise Halogenated Nanographene Assemblies via Bottom-Up Chemical Synthesis
Source: ACS Appl Mater Interfaces. 2024 May 24;16(22):29016–28. doi: 10.1021/acsami.4c02545 (PMC11163403; doi:10.1021/acsami.4c02545)
Supplement: Supplementary file 1 — am4c02545_si_001.pdf [file am4c02545_si_001.pdf]

Supporting Information

Lithium-ion Dynamic and Storage of Atomically Precise Halogenated Nanographene Assemblies via Bottom-up Chemical Synthesis

Febri Baskoro[1||], Hui Qi Wong[1,2,3||], Svetozar Najman[4], Po-Yu Yang[4], Jazer Jose H. Togonon[1], Yi-Chi Ho[1], Mei-Chun Tseng[1], Der-Lii M. Tzou[1], Yu-Ruei Kung[5], Chun-Wei Pao[4,6] and Hung-Ju Yen[1,*]*

[1] Institute of Chemistry, Academia Sinica, 128 Academia Road, Section 2, Nankang, Taipei 11529, Taiwan

[2] Sustainable Chemical Science and Technology Program, Taiwan International Graduate Program (TIGP), Academia Sinica, Taipei 11529, Taiwan

[3] Department of Chemical Engineering, National Taiwan University, Taipei 10617, Taiwan

[4] Research Center for Applied Sciences, Academia Sinica, Taipei 11529, Taiwan

[5] Department of Chemical Engineering and Biotechnology, Tatung University, Taipei 10452, Taiwan

[6] Department of Photonics, National Yang Ming Chiao Tung University, Hsinchu 30010, Taiwan

Corresponding authors: cwpao@gate.sinica.edu.tw (C.-W. Pao); hjyen@gate.sinica.edu.tw (H.-J Yen)

Supplementary Notes:

Supplementary Note 1 - HOMO-LUMO energy levels of NGs

The HOMO-LUMO energy levels of halogenated NGs were estimated using cyclic voltammetry (CV) and UV-visible spectroscopy methods. In brief, different mass of NGs samples and Ferrocene were dissolved in 7mL *o*-dichlorobenzene (DCB) with 0.10M tetrabutylammonium perchlorate (TBAP) to obtain a 1mM saturated solution. The resulting solution was constantly mixed and heated up to 100 °C overnight to maximize the dissolution of the materials in the solvent. CV measurement was then employed in a bipotentiostat (CHI 7061E) using a three-electrode setup with the glassy carbon electrode (GCE), Ag/AgCl, and a platinum (Pt) wire as the working electrode, reference electrode, and counter electrode, respectively. The CV measurements were performed using a scan rate of 50 mV s^{-1} and was measured from 0 V to 1.60 V. Onset potentials from the CV curve of the different NGs and Ferrocene (Figure S18) were then used to estimate to HOMO levels.

The UV-Visible spectroscopy method has been used to further elucidate the energy bandgap of edge-functionalization. All UV-Vis spectroscopy measurements were carried out in spectrometer (Agilent Technologies Cary 8454 UV-Vis). In brief, aliquots of the saturated solution further diluted with DCB with 0.10M TBAP to control the absorbance of up to 1 au. The spectrum of the solution was recorded from 290 nm to 1200 nm. The bandgap of the material was then calculated using the onset wavelength (λ_{onset}) of NGs spectrum. The HOMO-LUMO level and band gap energy of NGs were estimated using following formula:

$$E_{HOMO} = -(E_{OX}^{onset} - E_{Ferrocene}^{onset} + 4.8)\ (eV) \tag{1}$$

$$E_{gap} = 1240/\lambda_{onset}\quad (eV) \tag{2}$$

$$E_{gap} = 1240/\lambda_{onset} \quad (eV) \tag{3}$$

As shown in Figure S18, the onset oxidation potential of NGs were tuned due to halogen functionalization. The onset oxidation potential of **Pure-HBC** has been increased from 0.582 to 0.622 V by attaching fluorine on the edge of NGs (**6F-HBC**), resulting the reducing HOMO energy level of -4.932 eV (**Pure-HBC**) to -4.972 (**6F-HBC**) (Figure 3b). Furthermore, the onset oxidation potential of NGs gradually decreased along with the decreasing electronegativity of halogen from F > Cl > Br > I (Figure S18), resulting the increasing HOMO energy level (Figure 3b). This indicate that the electron density of NGs could be regulated by halogen functionalization.

Additionally, the λ_{onset} of 2D NGs is shifted along with the halogen functionalization on the edge termination (Figure S19). As shown in Figure S19, the **6F-HBC** has the lowest λ_{onset} of 474.35 nm followed by **6Cl-HBC**, **6Br-HBC** and **6I-HBC** with value of 510.0, 521.26, and 560.12 nm, respectively. This shifting on the λ_{onset} suggesting the change in the E_{gap} of NGs. Among the halogenated NGs, **6F-HBC** is found to have highest E_{gap} (2.61 eV), indicating a maximum charge polarization resulted higher E_{gap} of halogenated NGs and possibly enhanced the materials stability. Furthermore, the E_{gap} of halogenated NGs are in good agreement with the reducing electronegativity of halogen functionalization as follow: **6Cl-HBC** (2.43 eV), **6Br-HBC** (2.38 eV), and **6I-HBC** (2.21 eV) (Figure 3b). This suggesting that the reactivity of halogenated NGs strongly depends on the electronegativity of halogen atoms.

Supplementary Note 2 - Theoretical capacity of NGs

It has been reported that Li-ions can be absorbed on both side of graphene sheets[1-2] as well as the defect/edge sites, leading significant increase on Li^+ adsorption sites thus increase its theoretical capacity. However, due to the possible repulsion forces between Li^+, the exact number of adsorptions sites could be varied and dependent on *d*-spacing, edge structure, and layers of carbon planes in graphene.[3] In addition, previous study has been reported that the zig-zag edge sites of graphene has 1 Li^+ / 2 edge C, while the armchair has 1 Li^+ / 4 edge C.[4] According to this model and LiC_6 structure is assumed, the total Li^+ in halogenated NGs could be estimated. Acording to this model, the total Li^+ adsorption on **Pure-HBC**, **6F-HBC**, **6Cl-HBC**, **6Br-HBC**, and **6I-HBC** is 38, 32, 32, 32, and 32, respectively. Furthermore, the theoretical capacity of anode material could be estimated according to Faraday's law, which is described as:

$$C(\text{mA h } g^{-1}) = \frac{n \times F}{3.6M} \tag{4}$$

where C is the specific theoretical capacity, n is the accepted number of electrons (number of Li^+), F is the Faraday constant (96485 C mol^{-1}), and M is the molecular weight of the anode material.[5] Therefore, the theoretical capacity of **Pure-HBC**, **6F-HBC**, **6Cl-HBC**, **6Br-HBC**, and **6I-HBC** is estimated 1948, 1360, 1176, 861, and 671 mA h g^{-1}, respectively. However, the cycling performance of halogenated NGs (Figure 4b) was inconsistent with this theoretical capacity trend. Therefore, the Li^+ storage capacity of halogenated NGs could mainly depends on their structural and electronic properties.

Supplementary Note 3 - Li^+ storage mechanism in 2D halogenated-NGs

To confirm the Li storage mechanism in the 2D halogenated-NGs, *ex situ* XPS were carried out at the various charge/discharge stages. As shown in Figure S24a, It is clear that once the **Pure-HBC** is fully charged (0.02 V), an additional new peak appeared at a binding energy (BE) of 283.5 eV on the C1s spectra, along with the formation of LiC_6 at BE of 53.5 eV on the Li1s spectra (Figure S24b). These BEs further confirms a successfully lithiation process of **Pure-HBC**. Furthermore, no BE associated with the C-Li and LiC_6 formation can be observed after the **Pure-HBC** anode is fully discharge (3.0 V), suggesting a successful delithiation process. The successful lithiation/delithiation process can also be monitored for **6F-HBC** via formation/deformation of C-Li and LiC_6 on the C1s and Li1s spectra, respectively (Figure S25a and S25b). Interestingly, the **6F-HBC** depicting a significant higher intensity of LiF BE at 56.06 eV on the Li1s spectra during full charge process, and later drop dramatically at full discharge process, while maintaining specific BE of C-F (Figure S25c). This phenomenon suggests that the additional F atom on the edge of HBC significantly increases the Li adsorption capability due to the higher electronegativity. A good material capability is confirmed by the BE of C-F was well-preserved on the C1s (Figure S25a) and F1s (Figure S25c) spectra during electrochemical process.

In addition, similar lithiation/delithiation behavior of **6F-HBC** was also observed from **6Cl-HBC** and **6Br-HBC**. As shown in Figure S26a and S26b, **6Cl-HBC** exhibited formation/deformation of C-Li and LiC_6 on the C1s and Li1s spectra during full charge process, suggesting successful Li^+ insertion process. Moreover, a new doublet separation BE can be monitored at 200.7 (Li-Cl $2p_{1/2}$) and 199.1 (Li-Cl $2p_{3/2}$) eV on the Cl2p spectra during full charge process, and later dismissed on the full discharge process (Figure S26c). Meanwhile, **6Br-HBC** (Figure S27) also depicting a C-Li and LiC_6 on the C1s and Li1s

spectra, as well as a doublet separation BE of Li-Br at 69.1 (Li-Br $3d_{5/2}$) and 70.2 (Li-Br $3d_{3/2}$) eV on the Br3d spectra during full charge process, suggesting successful Li^+ insertion process. This indicates that the Cl and Br atom also has an ability to attract Li^+ during charge process. Interestingly, **6I-HBC** exhibits a distinct chemical behavior during charge/discharge process. As shown in Figure S28a and S28b, although the successful Li^+ insertion can be confirmed by the appearance of C-Li (C1s spectra) and LiC_6 (Li1s spectra) at full charge stage, the specific BE of C-I was found disappeared on the C1s spectra along with the formation of LiI (Li1s spectra) and Li-I (I $3d_{5/2}$ spectra; Figure S28c). Furthermore, the specific BE of C-I was not recovered on the C1s spectra after the **6I-HBC** at fully discharge state (Figure S28a). Meanwhile, the BE of LiI and Li-I remained on the **6I-HBC** Li1s and I $3d_{5/2}$ spectra at full discharge state, along with the appearance new BE of I^0 on the I $3d_{5/2}$ spectra (Figure S28c). This further suggests that the iodine atom was detached from the HBC structure and consumed during charge process, thus it cannot be recovered when the **6I-HBC** anode was returned to a fully discharged. The distinct behavior of **6I-HBC** during electrochemical process can be associated with the intrinsic electronic property of **6I-HBC**. As shown in Figure 3b, **6I-HBC** exhibited the lowest LUMO energy and E_{gap}, thus further increasing its reactivity. This behavior also in good agreement with the CV on the Figure 4a, depicting additional redox activity at higher potential.

Supplementary Note 4 - Diffusion coefficient calculation based on the Warburg impedance

The electrochemical impedance spectroscopy (EIS) is a powerful technique to probe electrical properties of materials which are associate their physicochemical process, such as charge transfer, ionic charge carries, mass transport through diffusion and convection.[6] According to the Nyquist plot data, the conduction through electrolyte, separator and wires is reflected in the high frequency region.[7] Meanwhile, the mid frequency region depicted the charge transfer and kinetic reactions.[8-9] Furthermore, the low frequency region, which is typically with slope of 45°, represents the diffusion limited region in the solid phase and is typically characterized by the Warburg impedance.[10] The Warburg impedance can be defined by :

$$Z' = \sigma/\omega^{\frac{1}{2}} - j\,\sigma/\omega^{\frac{1}{2}} \tag{5}$$

$$|-Z"| = \sqrt{2}\,\sigma/\omega^{\frac{1}{2}} \tag{6}$$

where Z′ and Z″are real and imaginary impedance, respectively. While, ω is the angular frequency and σ is the Warburg coefficient. The Warburg coefficient for NGs can be determined by the slope of the Warburg plot (Z′ against $1/\omega^{\frac{1}{2}}$), as shown in Figure S30. In Li ion battery, the relationship of Warburg coefficient (σ) and the diffusion coefficient (D_{Li}) is given by :

$$\sigma = \frac{RT}{n^2F^2A\sqrt{2}}\left(\frac{1}{D_{Li}^{1/2}C_{Li}}\right) \tag{7}$$

where R is ideal gas constant, T is absolute temperature, n is the number of electron transferred, F is Faraday's constant, A is the area of the electrode, D_{Li} and C_{Li} are the diffusion coefficient and concentration of Li^+, respectively.[11]

Supplementary Note 5 - Charge storage behavior analysis based on sweep rate CV

In general, the total stored charge in a CV curve can be define into three components: (a) the faradaic contribution from the Li^+ ion insertion process; (b) the faradaic contribution from the charge-transfer process with surface atoms, referred to as pseudocapacitance; (c) the non-faradaic contribution from the double layer effect.[12-13] These faradaic contribution from insertion process (diffusion control) and capacitive effects can be expressed by:

$$i = av^b \tag{8}$$

where i is current response to the scan rate v, while a and b are constants. The b value can be obtained from the slope of log i vs log v. If the b value close to 0.5, it indicates a half-infinite linear diffusion-controlled process, whereas, a b value close to 1 indicates that the current is mainly generated from surface-controlled process (capacitive effects).[12, 14-16] Furthermore, the total charge stored for the reactions of capacitive (k_1v) and diffusion ($k_2v^{1/2}$) contributions at any fixed potential can be further quantified using the following formula:[12-13, 15, 17]

$$i = k_1v + k_2v^{1/2} \tag{9}$$

The k_1 and k_2 values can be determined by plotting $i/v^{1/2}$ vs $v^{1/2}$.

Supplementary Note 6 - Density functional theory (DFT) studies in 2D halogenated-NGs

The effect of halogen atom on the structural properties

To verify the structural properties of 2D halogenated-NGs, a series of density functional theory (DFT) simulations have been performed for a variety of structures to determine the impacts of halogen on the edge of NGs (Figure S35). Although the planar structure is more energetically favorable (Figure S36 – S39), **6F-HBC** exhibited a more profound higher energy difference between planar and non-planar structures than that of **6Cl-HBC**, **6Br-HBC**, and **6I-HBC** (Figure S40). Hence, it can be inferred that **6F-HBC** will preferably keep a planar structure rather than its non-planar structures at room temperature, while, the **6Cl-HBC**, **6Br-HBC**, and **6I-HBC** will alternate between their planar and non-planar structures. This observation is consistent with the XRD spectra (Figure S16). This phenomena could be ascribed to intramolecular hydrogen bonding in the 2D halogenated-NGs. As depicted in Figure S41a, it clear that the **6F-HBC** formed the strongest hydrogen bond (H-bond) and the shortest C-halogen bond than that of **6Cl-HBC**, **6Br-HBC**, and **6I-HBC**. This phenomena can also be observed at the ground state on the planar structure, in which **6F-HBC** has the lowest energy of -7.55 eV/atom (Figure S41b).

Single-ion (Li^+) adsorption study

To evaluate the active sites for Li^+ adsorption, we carried out series of single-ion adsorption calculations on all possible adsorption sites in the 2D halogenated-NGs (Figure S42). The adsorption energy in each particular sites in the NG flakes have been compiled in Table S2 and Figure S43. As shown in Table S2 and Figure S43, It is clear that adsorption at hollow sites is more stable for all 2D halogenated-NGs with the adsorption energy ranging around -1.1 to -1.3 eV. Notably, the Li atom placed on the inner most top sites during minimization assume the central hollow site position, and the Li atom on the outer top sites relax into the

inner or outer edge hollow sites positions, indicating a shallow potential energy surface in these areas of the flakes. The central top sites (T1) are stable in all NG flakes, although the adsorption energy is noticeably lower compared to the hollow sites due to a larger distance between the Li atom and the T1 site (~2.7 Å). Moreover, the distance between the Li atom and hollow sites has been found to be ~1.7 Å in all 2D halogenated-NGs and is consistent with the previous study.[18] Therefore, potential energy surface within the NG flake is predominantly shallow with the exception of the hollow sites, in which being steep potential wells and the most likely adsorption sites in all studied systems. Interestingly, two more stable Li^+ adsorption sites at H5 and S1 can be identified for **6F-HBC** with the energy of -0.7990 and -0.4636 eV, respectively, suggesting an additional active sites for Li^+ storage (Figure S43). As the result, the **6F-HBC** yields more preferential Li^+ adsorption with the average adsorption energy of -0.8063 eV than that of **6Cl-HBC** (-0.6617 eV), **6Br-HBC** (-0.6534 eV), and **6I-HBC** (-0.6386 eV) (Figure S44). These phenomena could be the most probable reason behind noticeable higher capacity compared to the other 2D halogenated NG flakes.

Li^+ adsorption pathway and stability of 2D halogenated NGs

Lastly, we elucidated the adsorption pathway and the stability of 2D halogenated-NGs with the presence of Li^+. Figure S45 shows the steps of Li^+ adsorption in the 2D halogenated-NGs. Additionally, Supplementary Movies 1 – 4 have been added to further visualized the Li^+ adsorption process. As depicted Figure S45a and Supplementary Movie 1, stronger hydrogen bond between fluorine and hydrogen on the **6F-HBC** forms potential adsorption for Li^+, due to strong interaction between fluorine and lithium, and its predominantly planar shape. This facilitates Li^+ to adsorb between the halogen and hydrogen atoms. For **6Cl-HBC** and **6Br-HBC**, Li^+ prefers adsorbing at the inner edge hollow sites for both **6Cl-HBC** (Figure S45b and Supplementary Movie 2) and **6Br-HBC** (Figure S45c and Supplementary Movie 3) via

intermediate interaction with halogen atom, due to a weaker H-bond and their more energetically favorable non-planar shapes. Notably, no preferential Li^+ adsorption at the edge sites (e.g. H5) can be observed for **6Cl-HBC** and **6Br-HBC**. Similar behavior has already been observed in an earlier study demonstrating Li diffusion through glucose layer can be facilitated due to low diffusion barriers and shallow potential wells originated from H-bonds.[19] Unlike **6F-HBC**, **6Cl-HBC**, and **6Br-HBC** that are stable during Li^+ insertion, the **6I-HBC** decomposes at the presence of Li^+ due to hydrogen bond breaking (Figure S45d and Supplementary Movie 4). As shown in Figure S45d and Supplementary Movie 4, the Li^+ adsorption at the edge of the **6I-HBC** flake potentially cleaved the C-I bond and formed a Li-I bond, indicating material instability. This phenomenon is further confirmed by the charge density plots of 2D halogenated NGs (Figure S46). As depicted Figure S46a – S46c, both the 2D charge density plots and the 3D 0.3 e/bohr^3 isosurfaces indicate a strong C-halogen bonding in the NG flakes for **6F-HBC**, **6Cl-HBC**, and **6Br-HBC** as evidenced by the continuous charge density distributions. In contrast, the charge density distribution is discontinuous between I and C atoms for the **6I-HBC** (Figure S46d), suggesting weaker C-I bonding. Hence, this explains the reason why the **6I-HBC** flake is unstable.

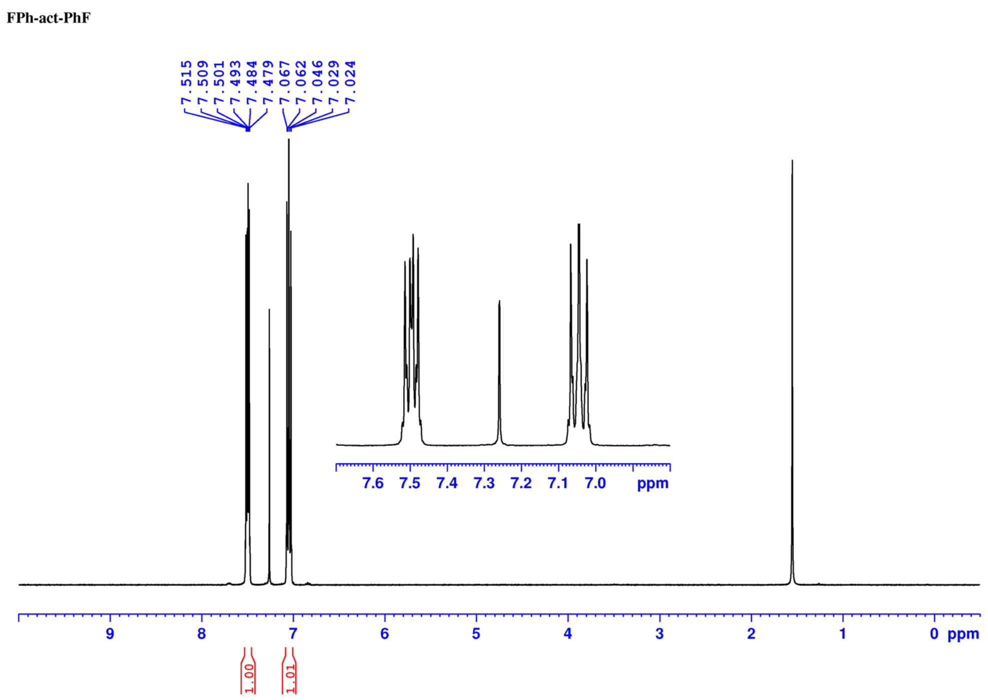

Figure S1. ^{1}H NMR spectra of **F-ac-F** in $CDCl_3$.

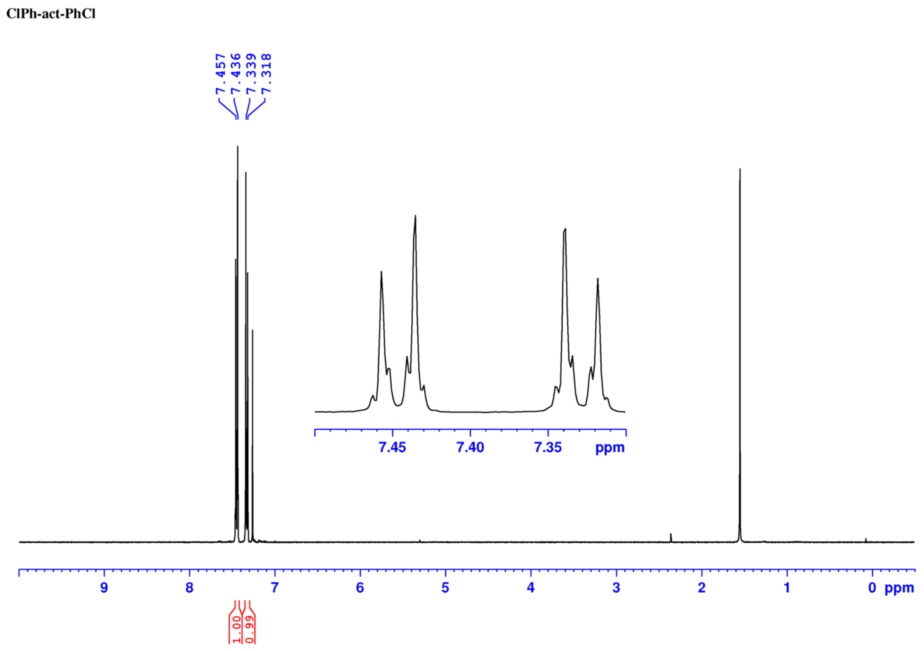

Figure S2. ^{1}H NMR spectra of **Cl-ac-Cl** in $CDCl_3$.

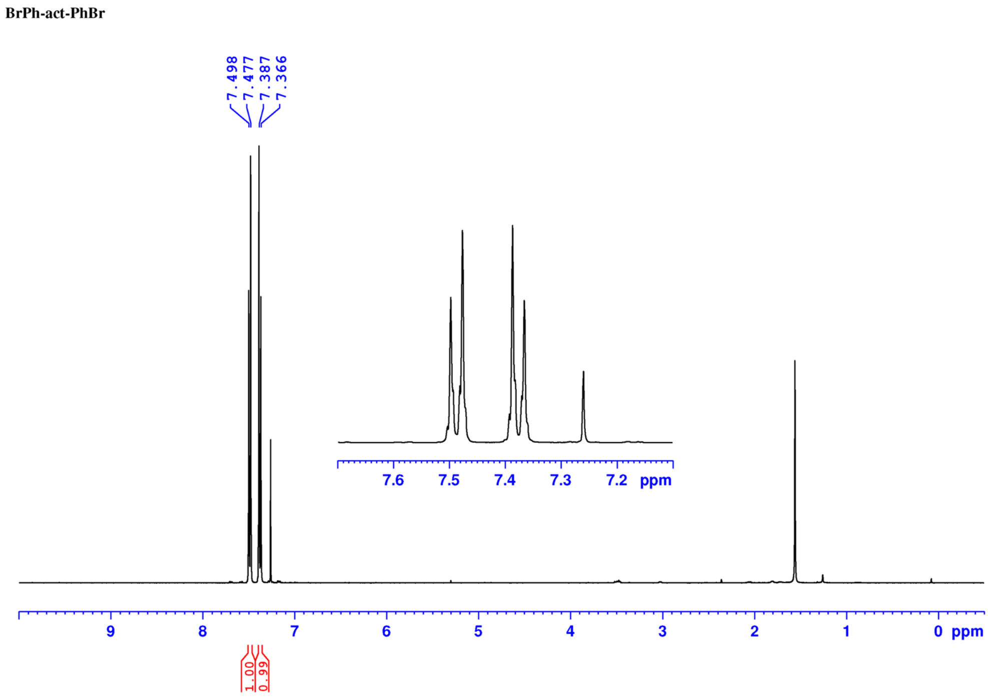

Figure S3. ^{1}H NMR spectra of **Br-ac-Br** in $CDCl_3$.

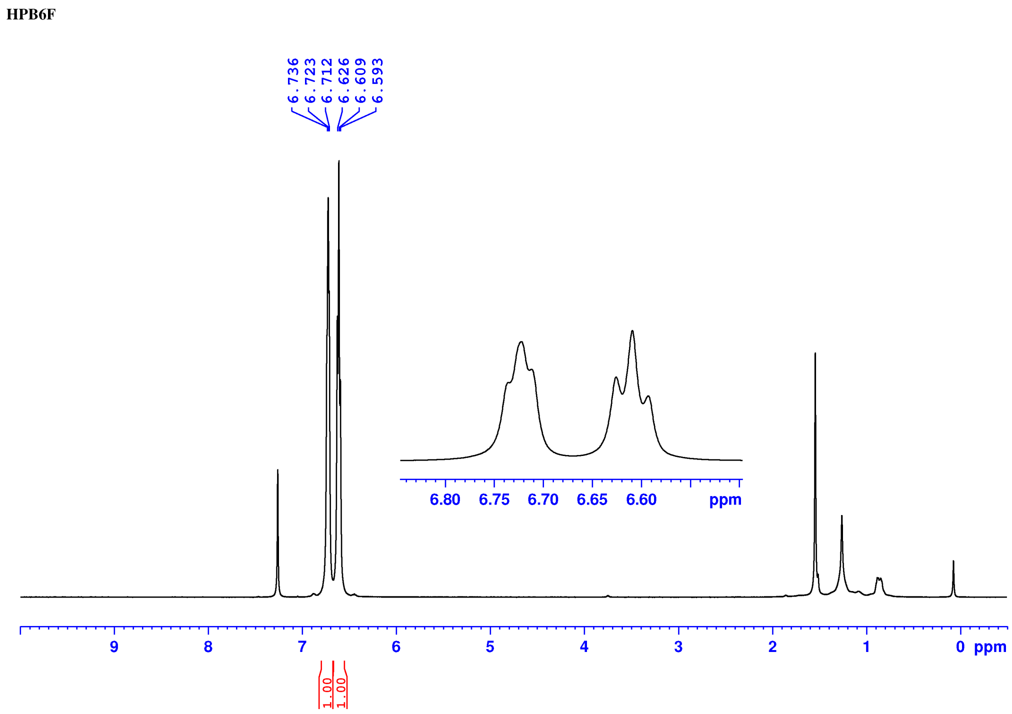

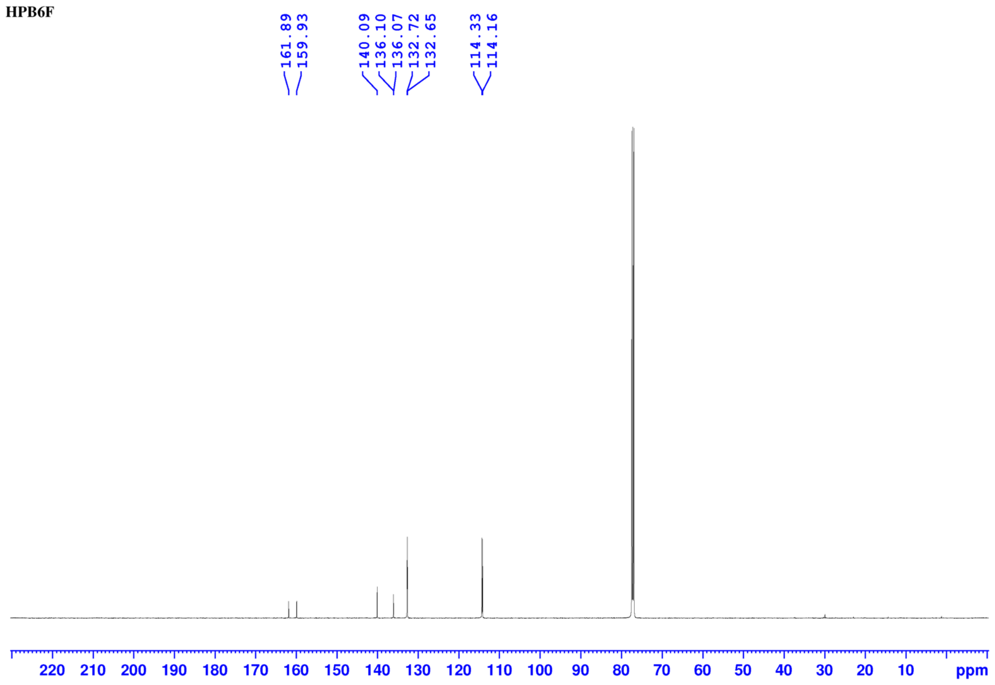

Figure S4. ^{1}H and ^{13}C NMR spectra of **6F-HPB** in $CDCl_3$.

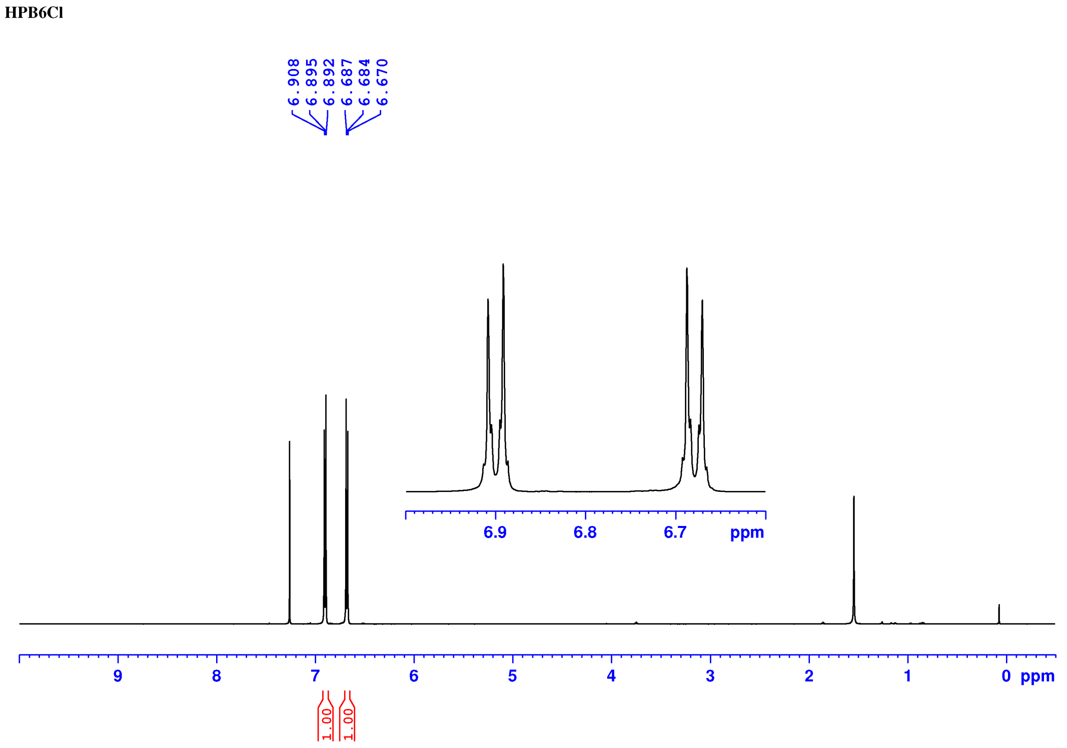

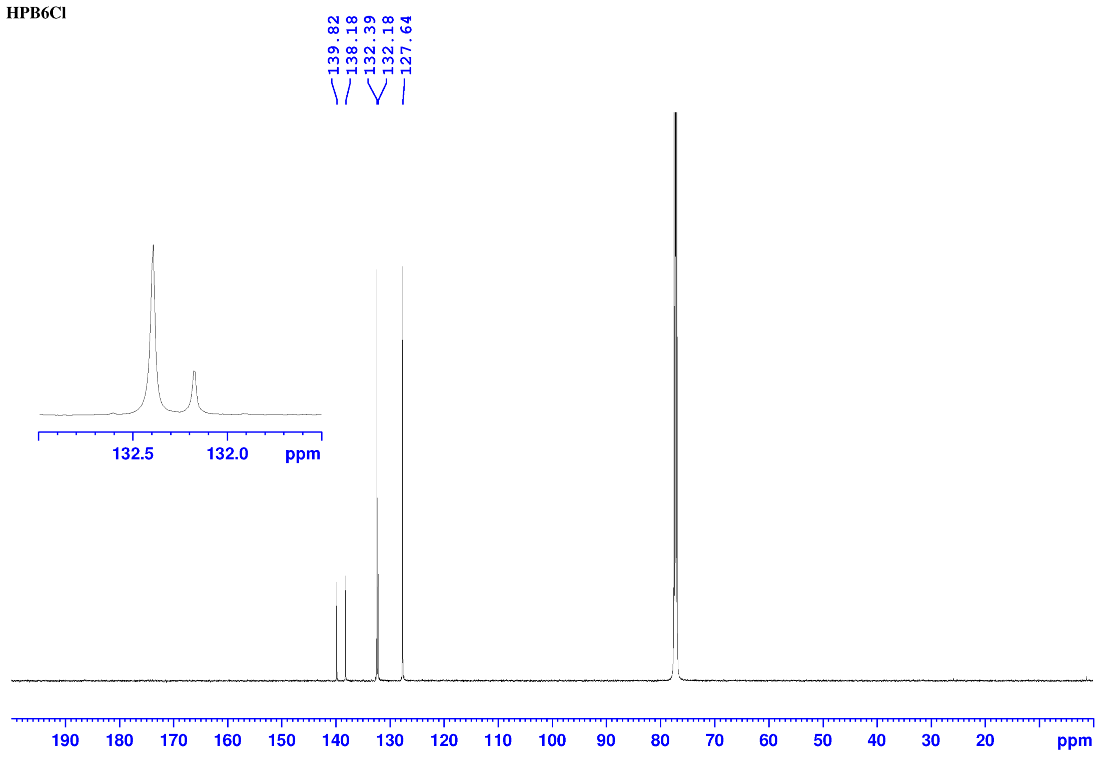

Figure S5. ^{1}H and ^{13}C NMR spectra of **6Cl-HPB** in $CDCl_3$.

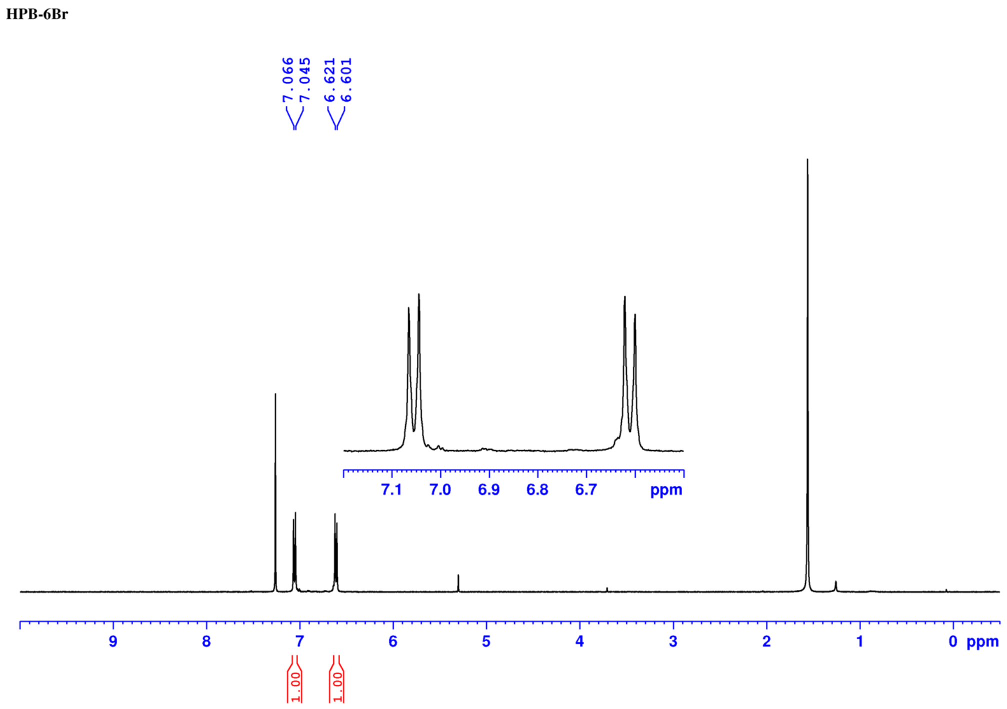

Figure S6. ^{1}H NMR spectra of **6Br-HPB** in $CDCl_3$.

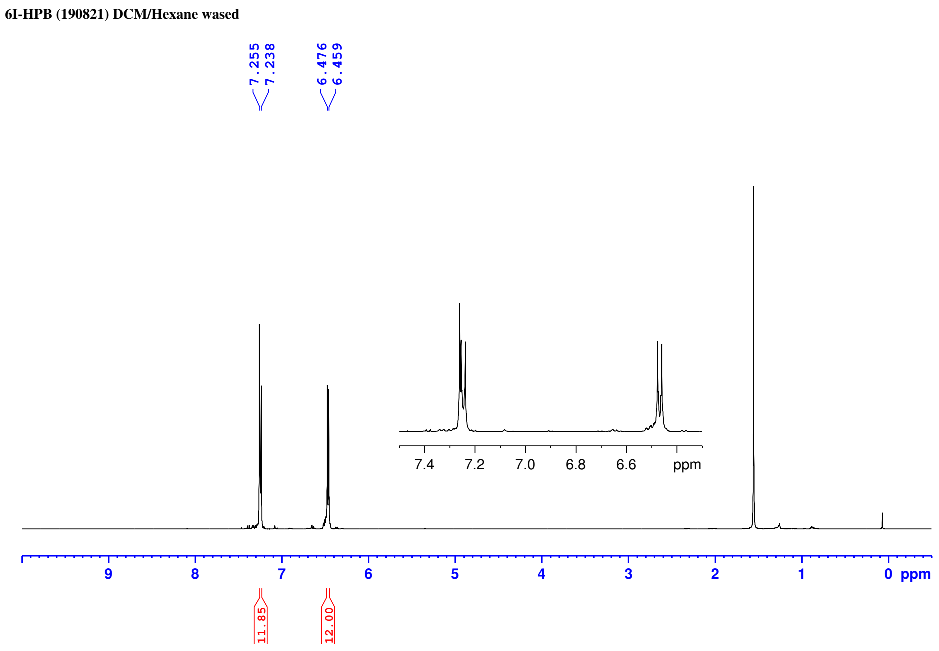

Figure S7. ^{1}H NMR spectra of **6I-HPB** in $CDCl_3$.

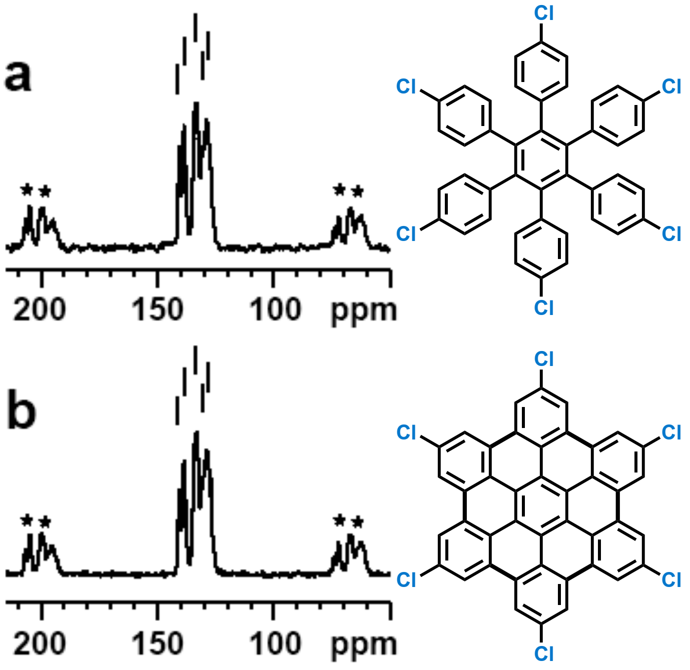

Figure S8. Solid-state $^{13}C\{^{1}H\}$ CP/MAS NMR analysis of (a) **6Cl-HPB** and (b) **6Cl-HBC**.

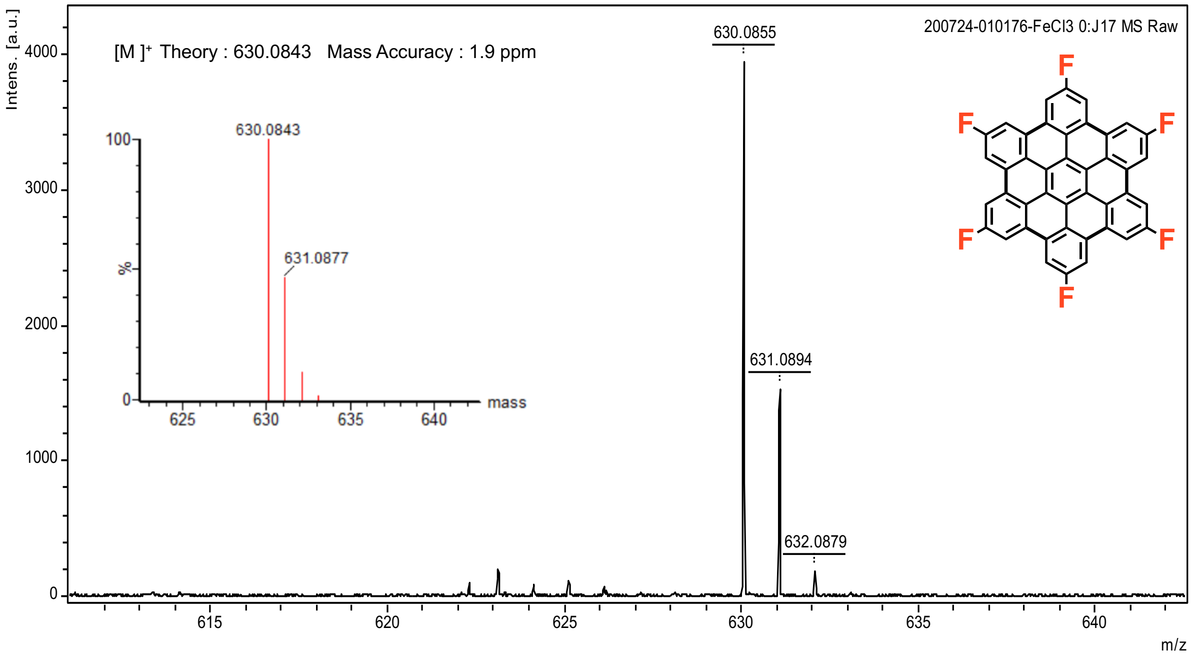

Figure S9. The MALDI-TOF MS spectrum of **6F-HBC**, inset: isotopic distribution and comparison with theoretically predicted spectrum (red column) for $C_{42}H_{12}F_6$.

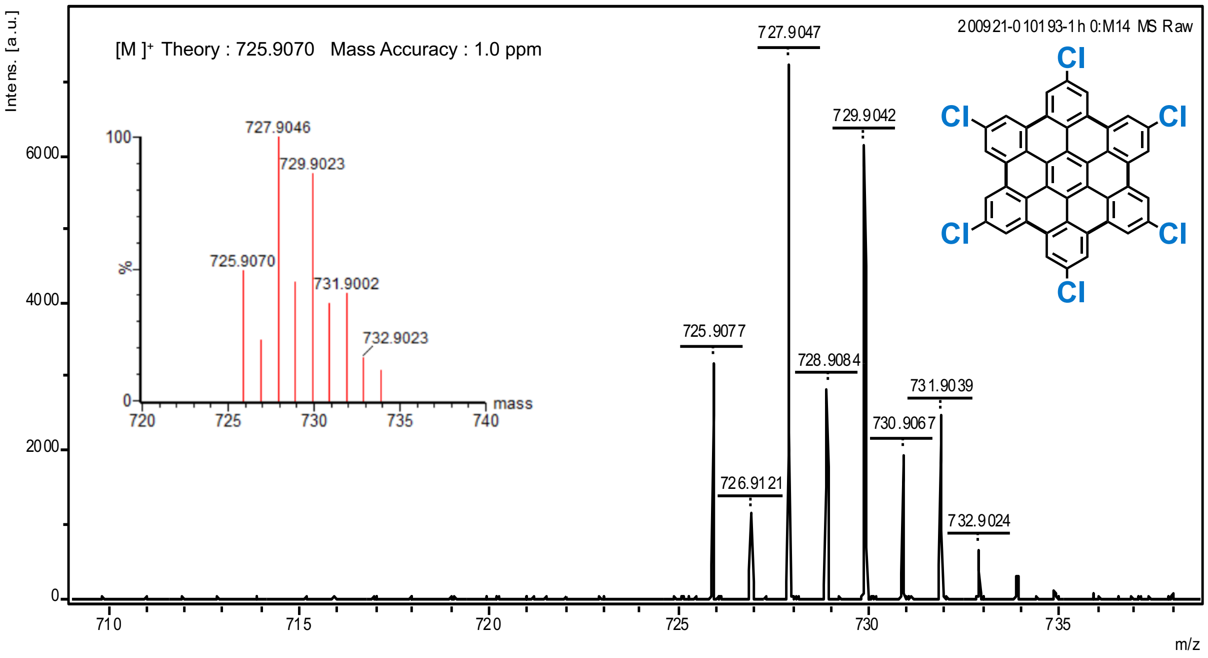

Figure S10. The MALDI-TOF MS spectrum of **6Cl-HBC**, inset: isotopic distribution and comparison with theoretically predicted spectrum (red column) for $C_{42}H_{12}Cl_6$.

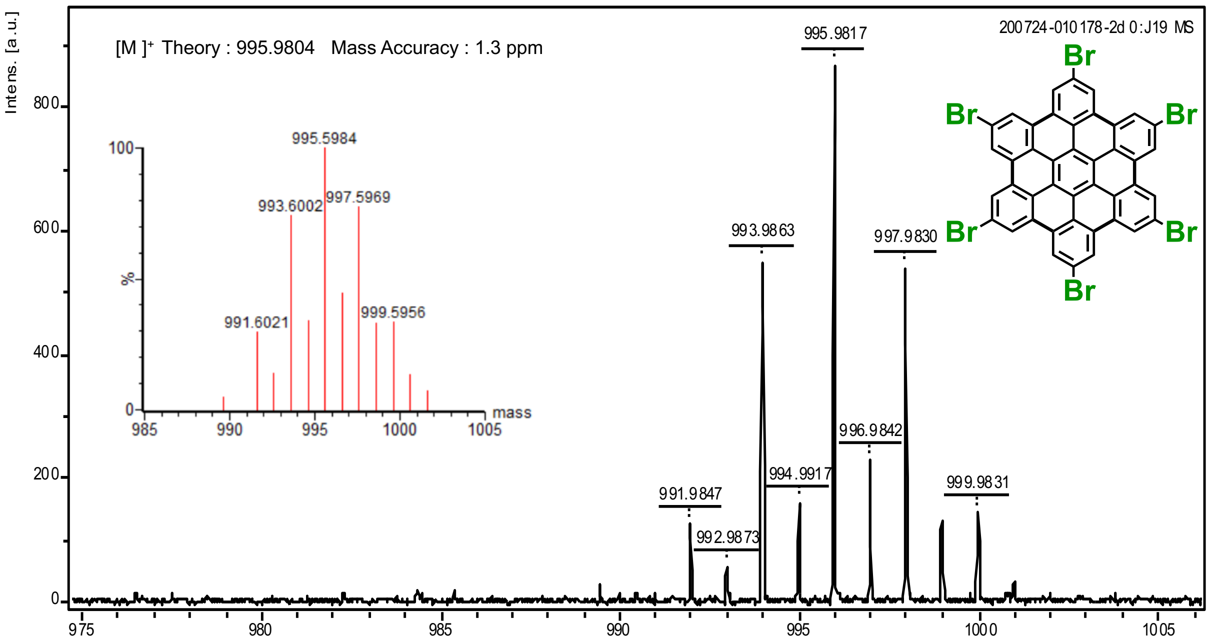

Figure S11. The MALDI-TOF MS spectrum of **6Br-HBC**, inset: isotopic distribution and comparison with theoretically predicted spectrum (red column) for $C_{42}H_{12}Br_6$.

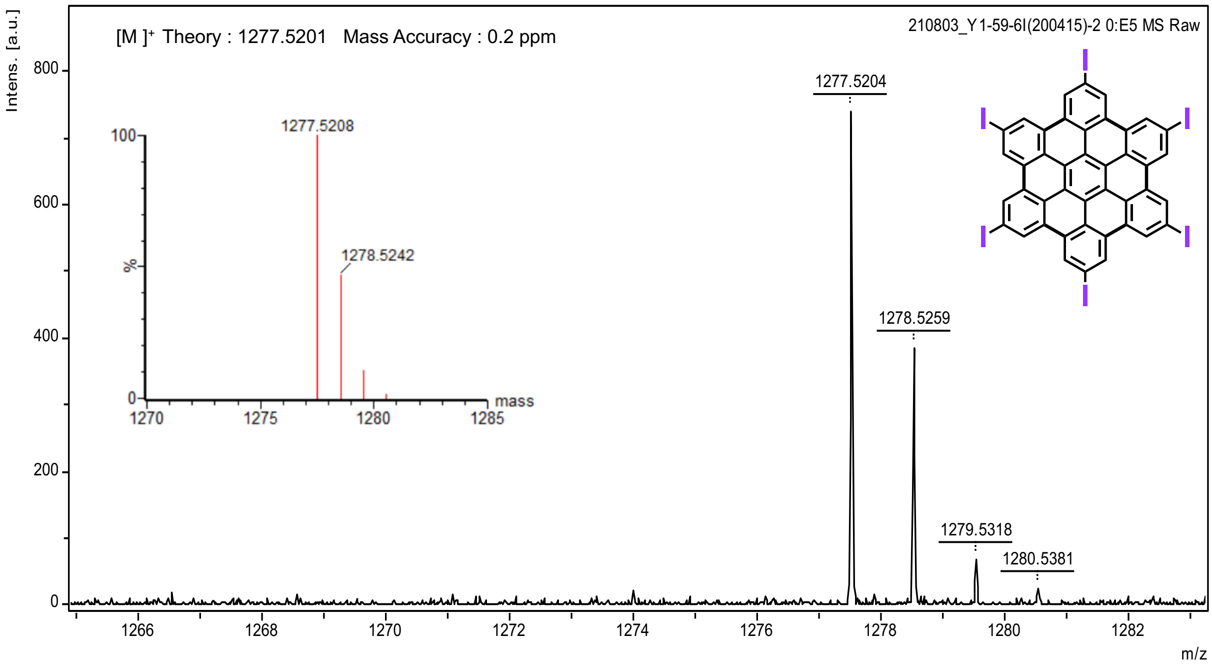

Figure S12. The MALDI-TOF MS spectrum of **6I-HBC**, inset: isotopic distribution and comparison with theoretically predicted spectrum (red column) for $C_{42}H_{12}I_6$.

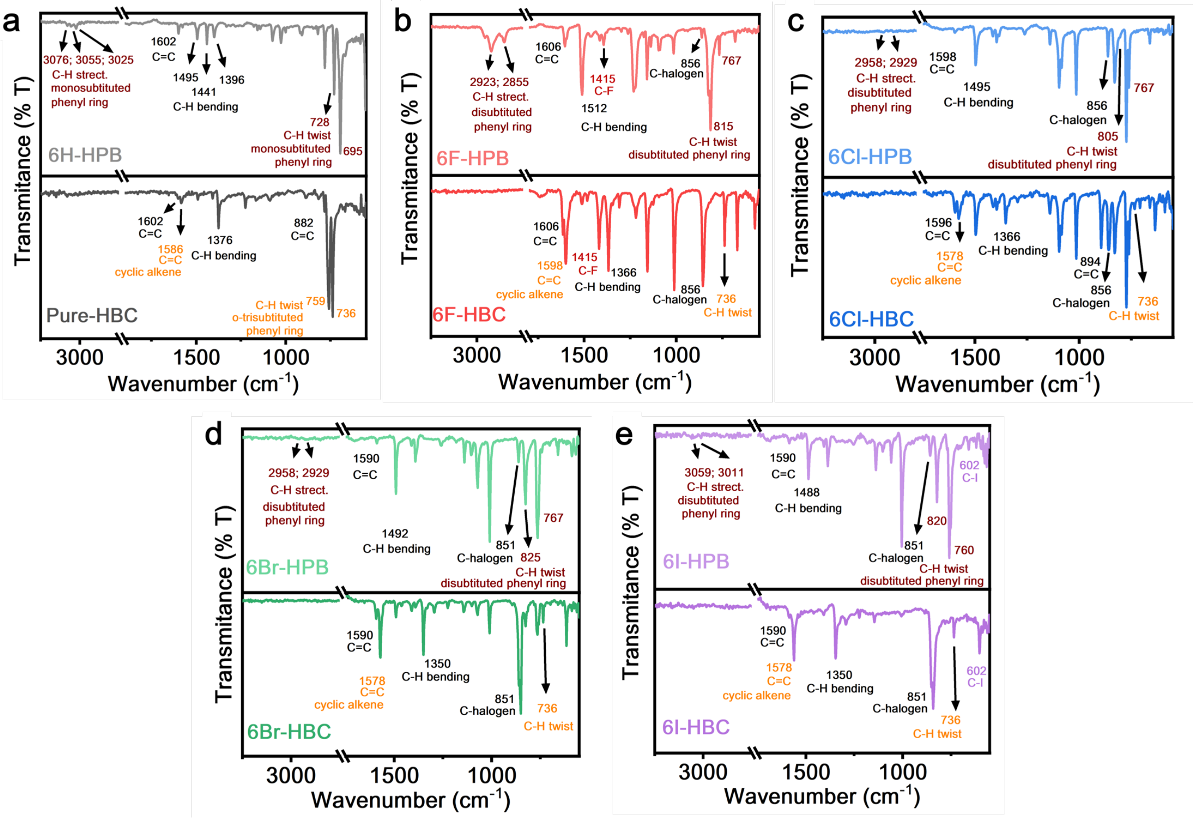

Figure S13. FT-IR spectra of all NGs. (a) **Pure-HBC**; (b) **6F-HBC**; (c) **6Cl-HBC**; (d) **6Br-HBC**; (e) **6I-HBC**.

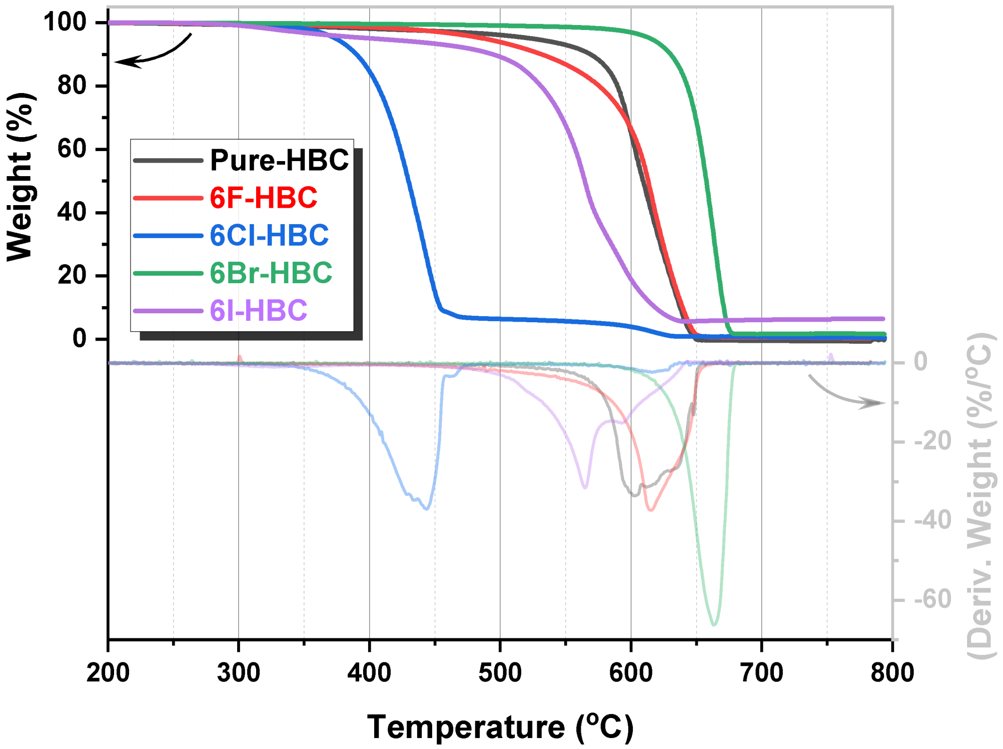

Figure S14. TGA thermograms of NG in nitrogen at a scan rate of 20 °C/min.

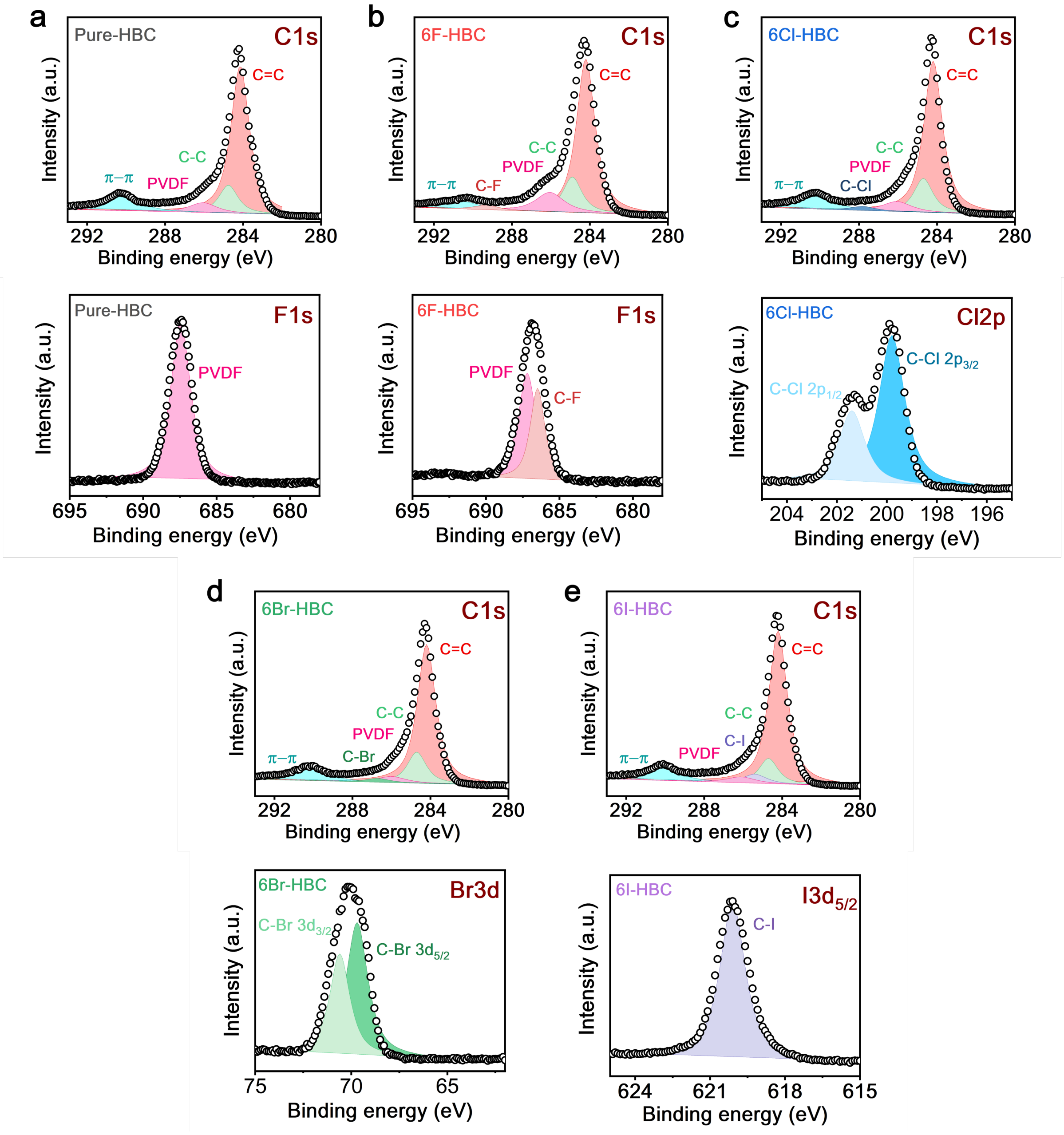

Figure S15. XPS spectra of all as prepared NG electrodes. (a) **Pure-HBC**; (b) **6F-HBC**; (c) **6Cl-HBC**; (d) **6Br-HBC**; (e) **6I-HBC**.

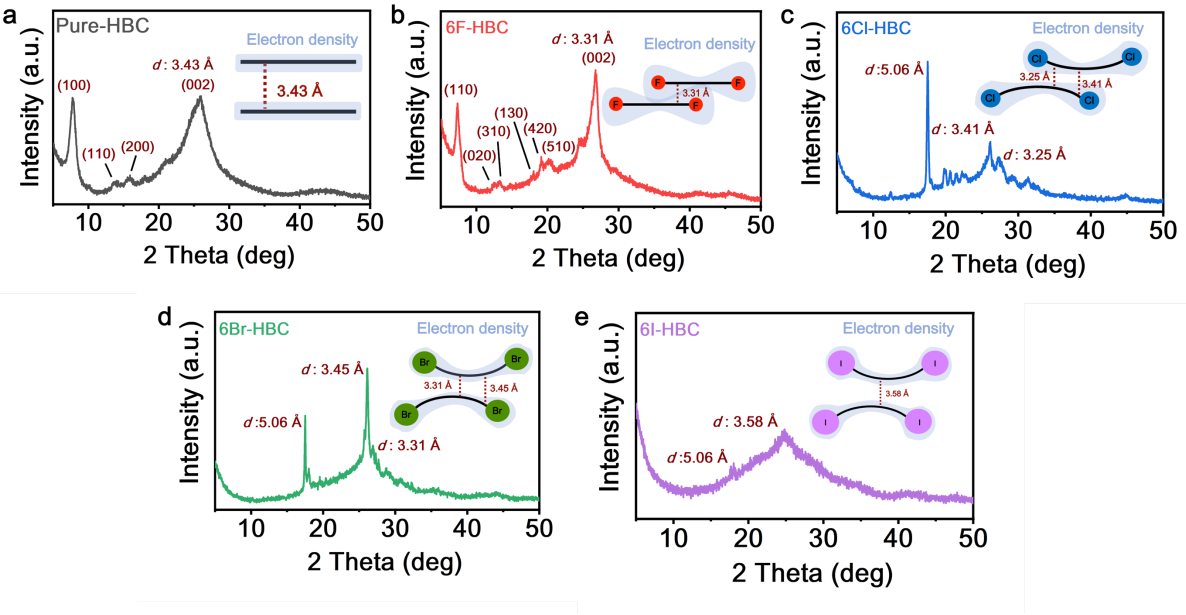

Figure S16. Powder XRD spectra of all NGs. (a) **Pure-HBC**; (b) **6F-HBC**; (c) **6Cl-HBC**; (d) **6Br-HBC**; (e) **6I-HBC**.

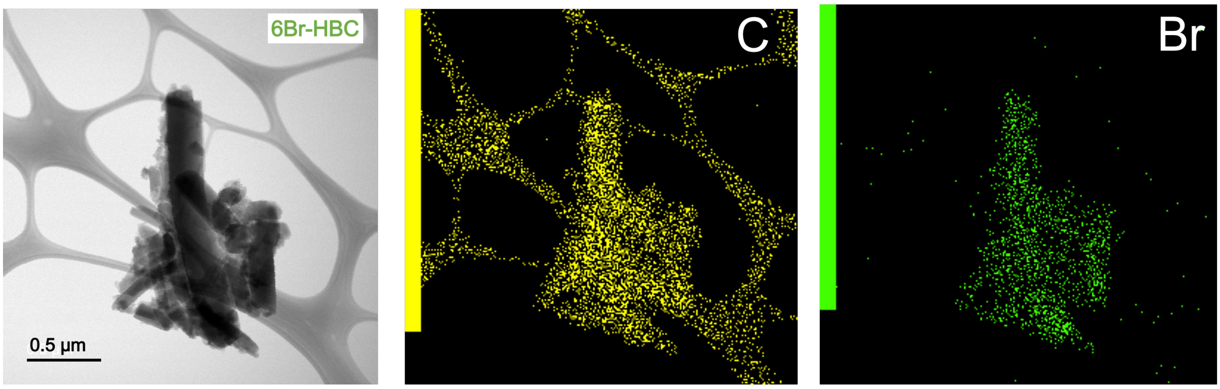

Figure S17. TEM/EDS images of 6Br-HBC.

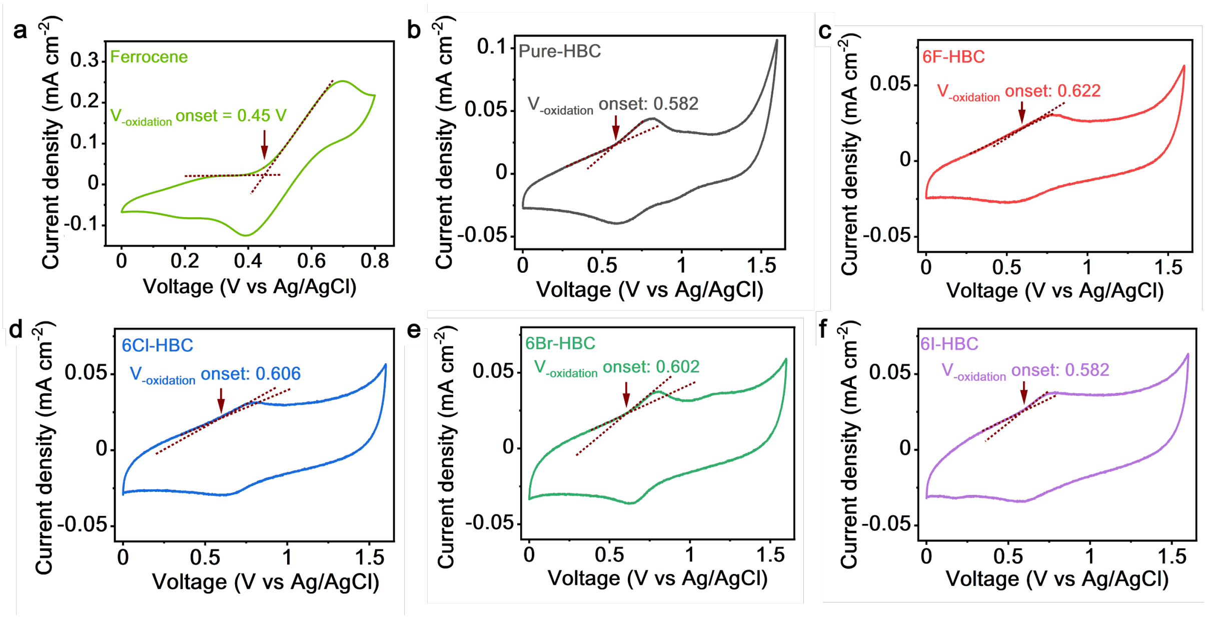

Figure S18. CV curves of Ferrocene (a) and NGs (b) **Pure-HBC** (c) **6F-HBC** (d) **6Cl-HBC** (e) **6Br-HBC** (f) **6I-HBC**.

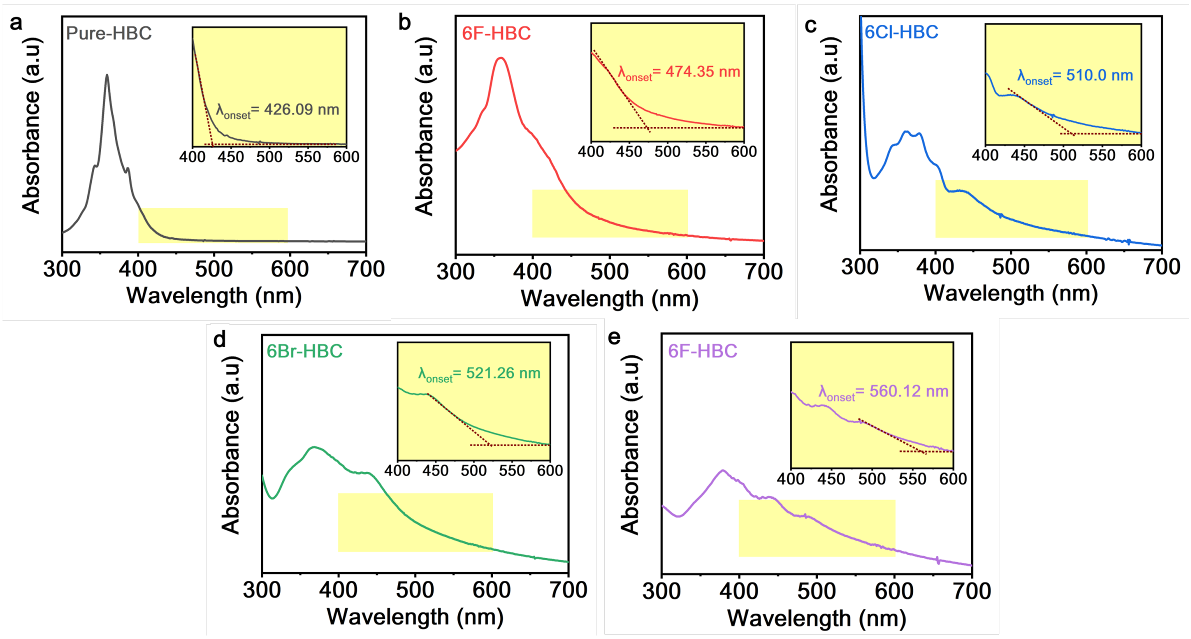

Figure S19. UV-visible spectra of NGs. (a) **Pure-HBC**; (b) **6F-HBC**; (c) **6Cl-HBC**; (d) **6Br-HBC**; (e) **6I-HBC**.

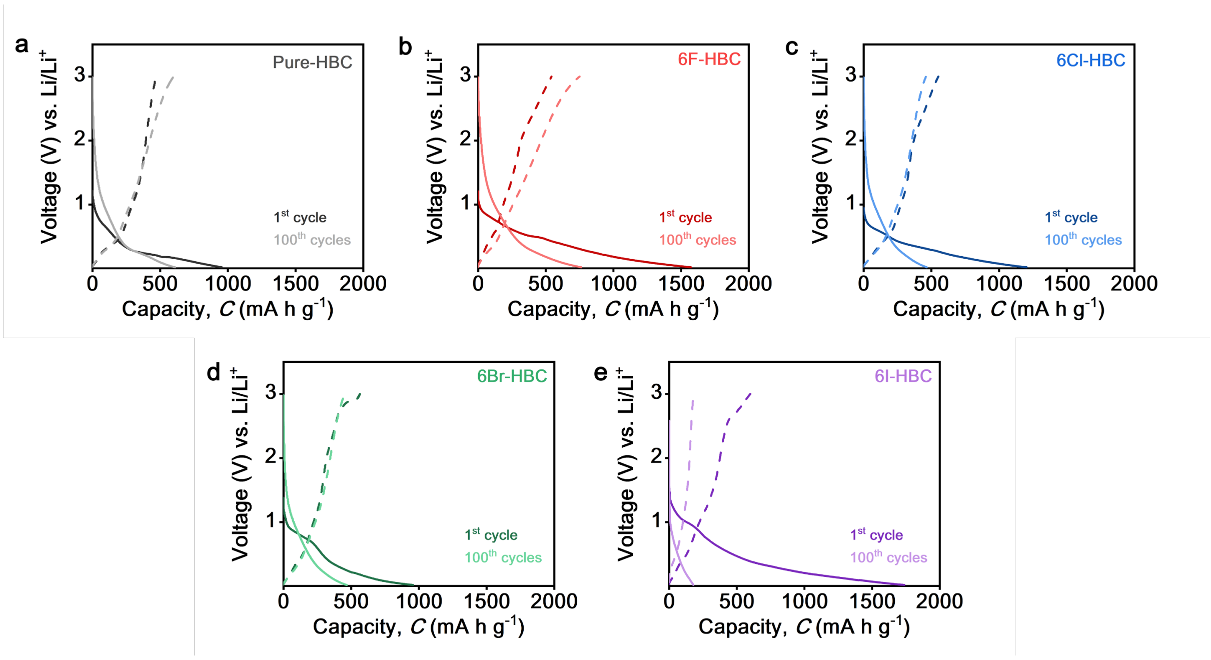

Figure S20. Galvanostatic charge-discharge profile of NGs at 0.1 A g^{-1} current density. (a) **Pure-HBC**; (b) **6F-HBC**; (c) **6Cl-HBC**; (d) **6Br-HBC**; (e) **6I-HBC**.

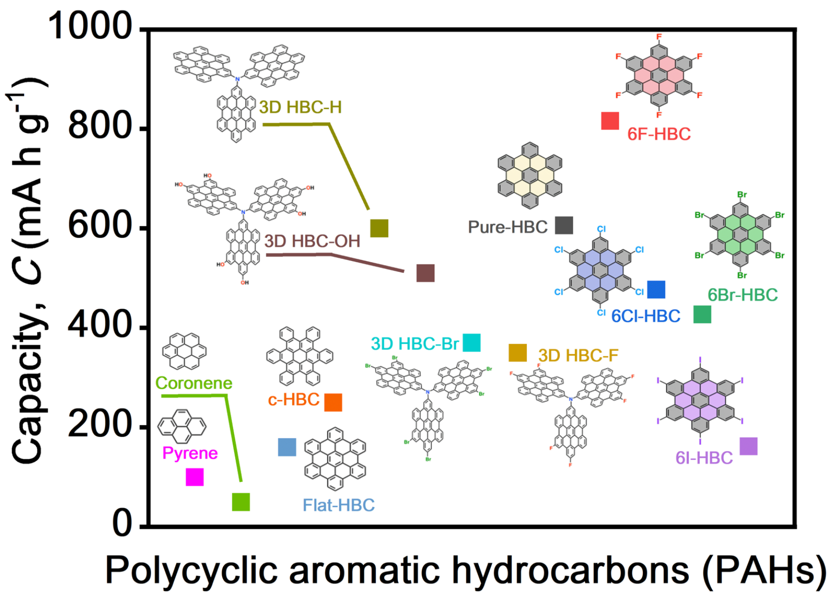

Figure S21. Plot of specific capacity with different polycyclic aromatic hydrocarbons (PAHs) as LIB anodes. Pyrene from ref. 20; Coronene from ref. 21; Flat-HBC from ref. 22; 3D HBC series from ref. 2.

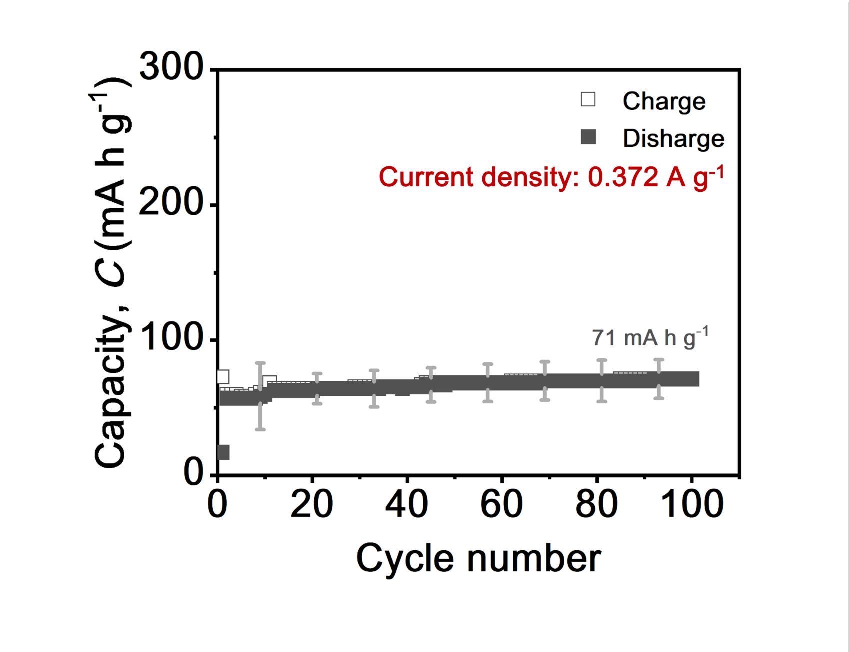

Figure S22. The electrochemical performance of **SP80** at current density of 0.372 A g^{-1}.

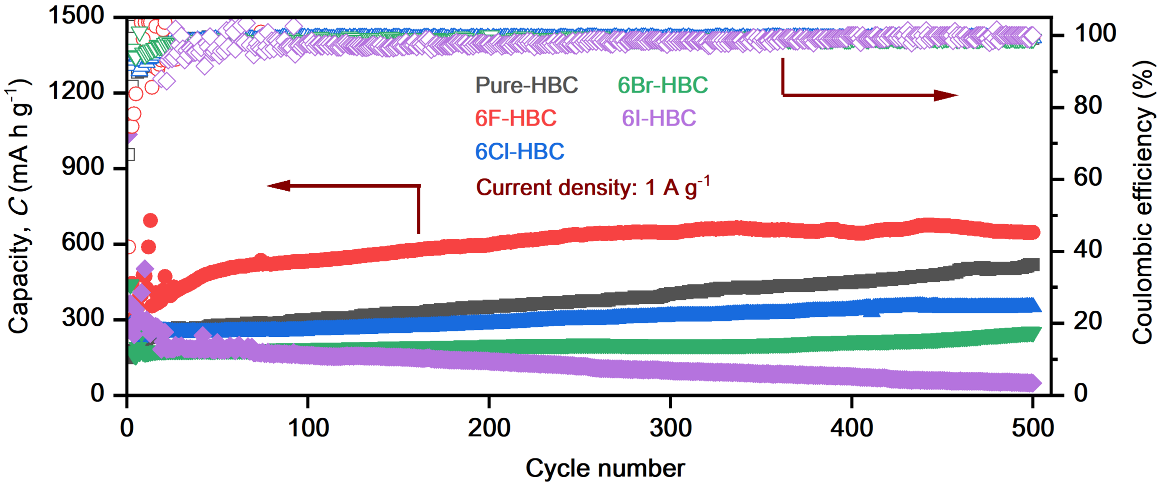

Figure S23. Long cycling performance of NG anodes at 1 A g^{-1}.

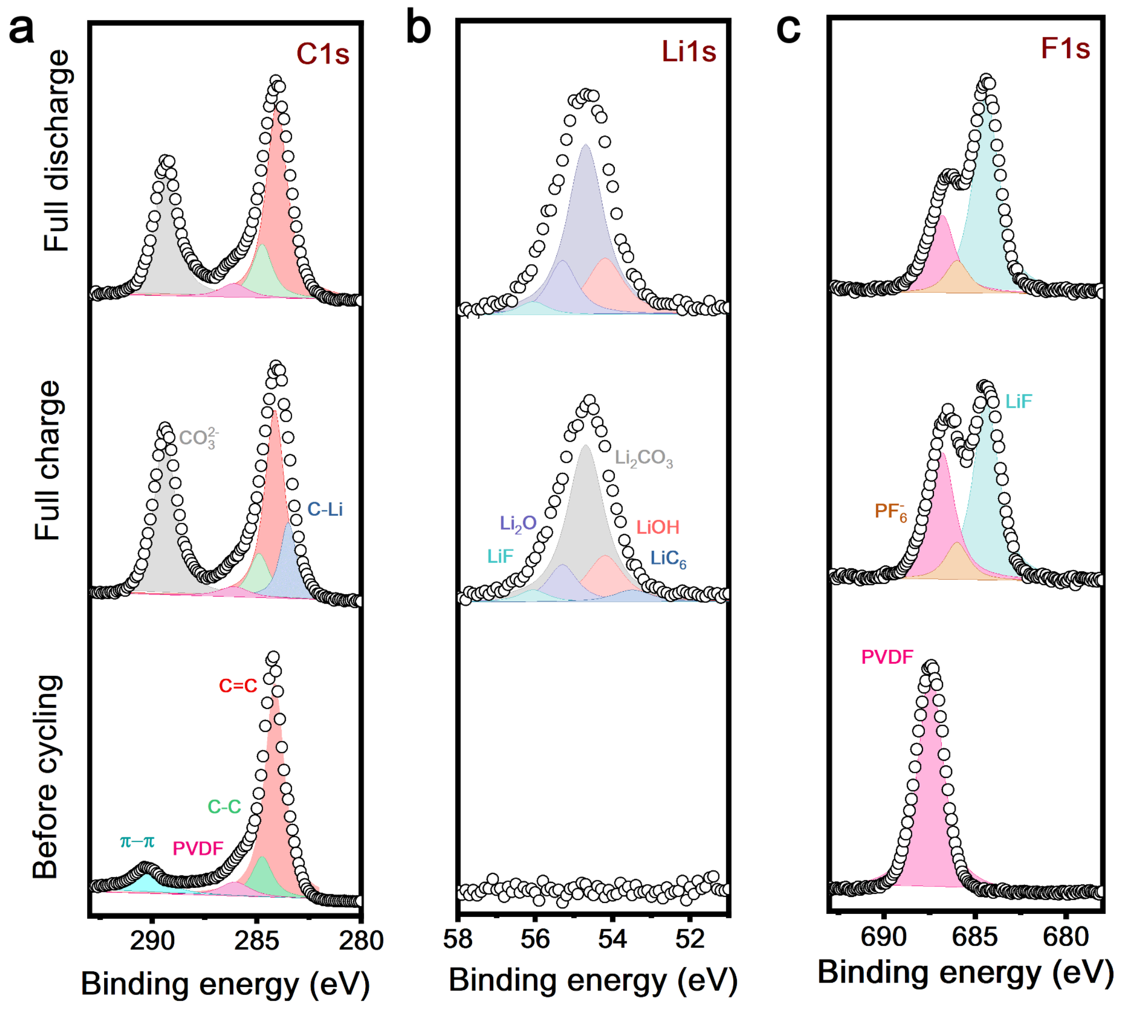

Figure S24. The ex situ XPS spectra of **Pure-HBC** at different charge/discharge stages: (a) C1s; (b) Li1s; (c) F1s. C1s spectra: C=C (284.15 eV); C-C (284.75 eV); CH_2-CF_2/PVDF (286.05 eV); π–π (290.2 eV); C-Li (283.5 eV); CO_3^{2-} (289.40 eV). Li1s spectra: LiC_6 (53.5 eV); LiOH (54.2 ev); Li_2CO_3 (54.7 eV); Li_2O (55.3eV); LiF (56.06 eV). F1s spectra: PVDF (687.45 eV); PF_6^- (686.0 eV); LiF (684.4 eV).

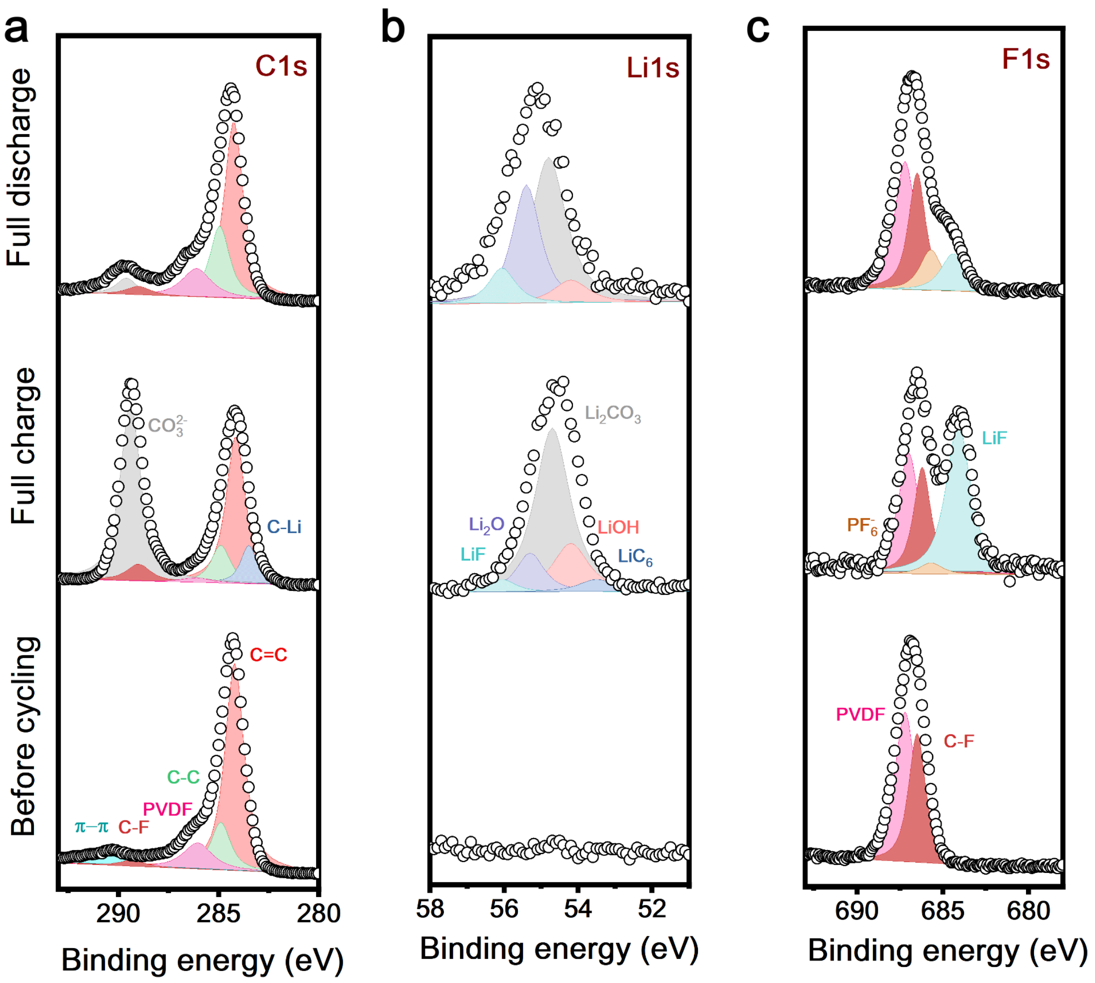

Figure S25. The ex situ XPS spectra of **6F-HBC** at different charge/discharge stages: (a) C1s; (b) Li1s; (c) F1s. C1s spectra: C=C (284.15 eV); C-C (284.75 eV); CH_2-CF_2/PVDF (286.05 eV); π–π (290.2 eV); C-F (289.3 eV); C-Li (283.5 eV); CO_3^{2-} (289.40 eV). Li1s spectra: LiC_6 (53.5 eV); LiOH (54.2 ev); Li_2CO_3 (54.7 eV); Li_2O (55.3eV); LiF (56.06 eV). F1s spectra: PVDF (687.45 eV); C-F (686.5 eV); PF_6^- (686.0 eV); LiF (684.4 eV).

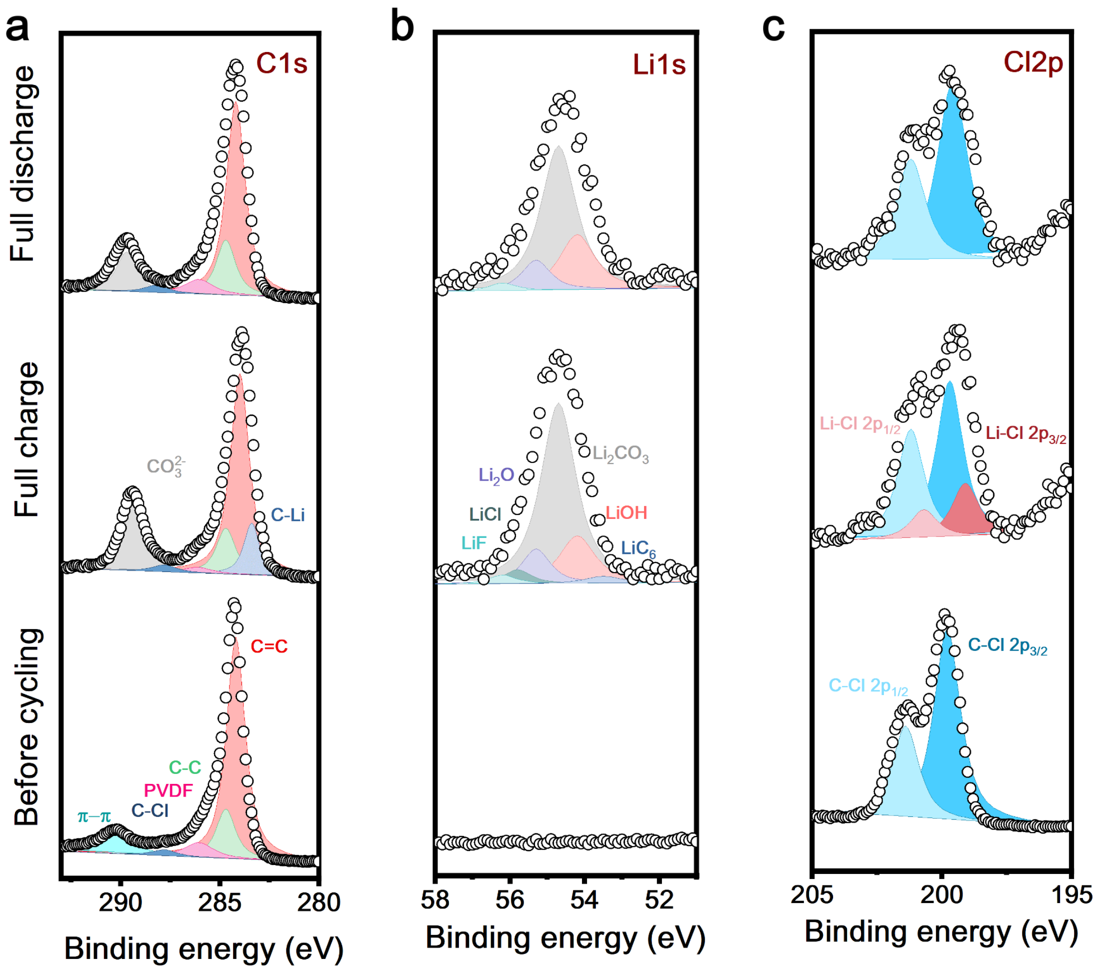

Figure S26. The ex situ XPS spectra of **6Cl-HBC** at different charge/discharge stages: (a) C1s; (b) Li1s; (c) Cl2p. C1s spectra: C=C (284.15 eV); C-C (284.75 eV); CH_2-CF_2/PVDF (286.05 eV); π–π (290.2 eV); C-Cl (287.8 eV); C-Li (283.5 eV); CO_3^{2-} (289.40 eV). Li1s spectra: LiC_6 (53.5 eV); LiOH (54.2 ev); Li_2CO_3 (54.7 eV); Li_2O (55.3eV); LiCl (55.8 eV); LiF (56.06 eV). Cl2p spectra: C-Cl $2p_{3/2}$ (199.8 eV); C-Cl $2p_{1/2}$ (201.4 eV); Li-Cl $2p_{3/2}$ (199.1 eV); Li-Cl $2p_{1/2}$ (200.7 eV).

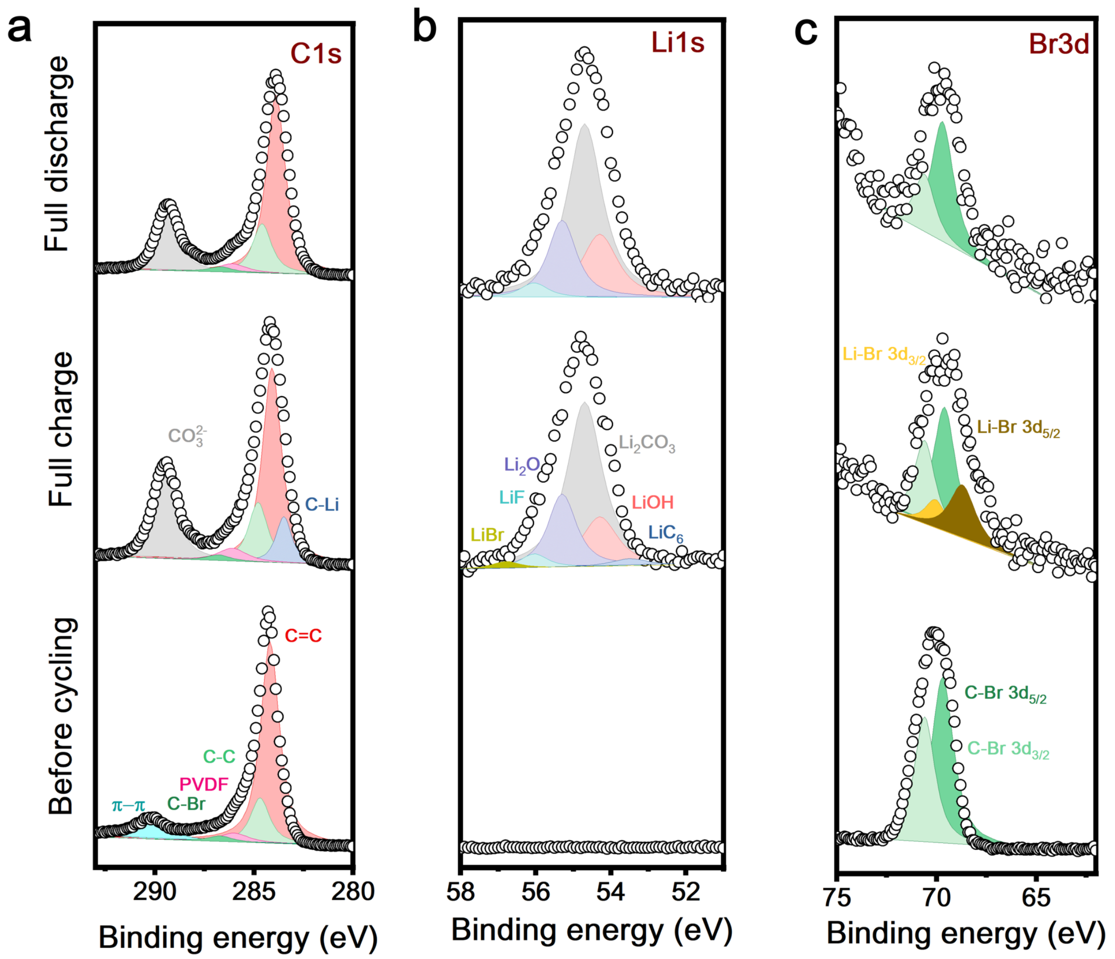

Figure S27. The ex situ XPS spectra of **6Br-HBC** at different charge/discharge stages: (a) C1s; (b) Li1s; (c) Br3d. C1s spectra: C=C (284.15 eV); C-C (284.75 eV); CH_2-CF_2/PVDF (286.05 eV); π–π (290.2 eV); C-Br (286.7 eV); C-Li (283.5 eV); CO_3^{2-} (289.40 eV). Li1s spectra: LiC_6 (53.5 eV); LiOH (54.2 ev); Li_2CO_3 (54.7 eV); Li_2O (55.3eV); LiBr (56.8 eV); LiF (56.06 eV). Br3d spectra: C-Br $3d_{5/2}$ (69.7 eV); C-Br $3d_{3/2}$ (70.6 eV); Li-Br $3d_{5/2}$ (69.1 eV); Li-Br $3d_{3/2}$ (70.2 eV).

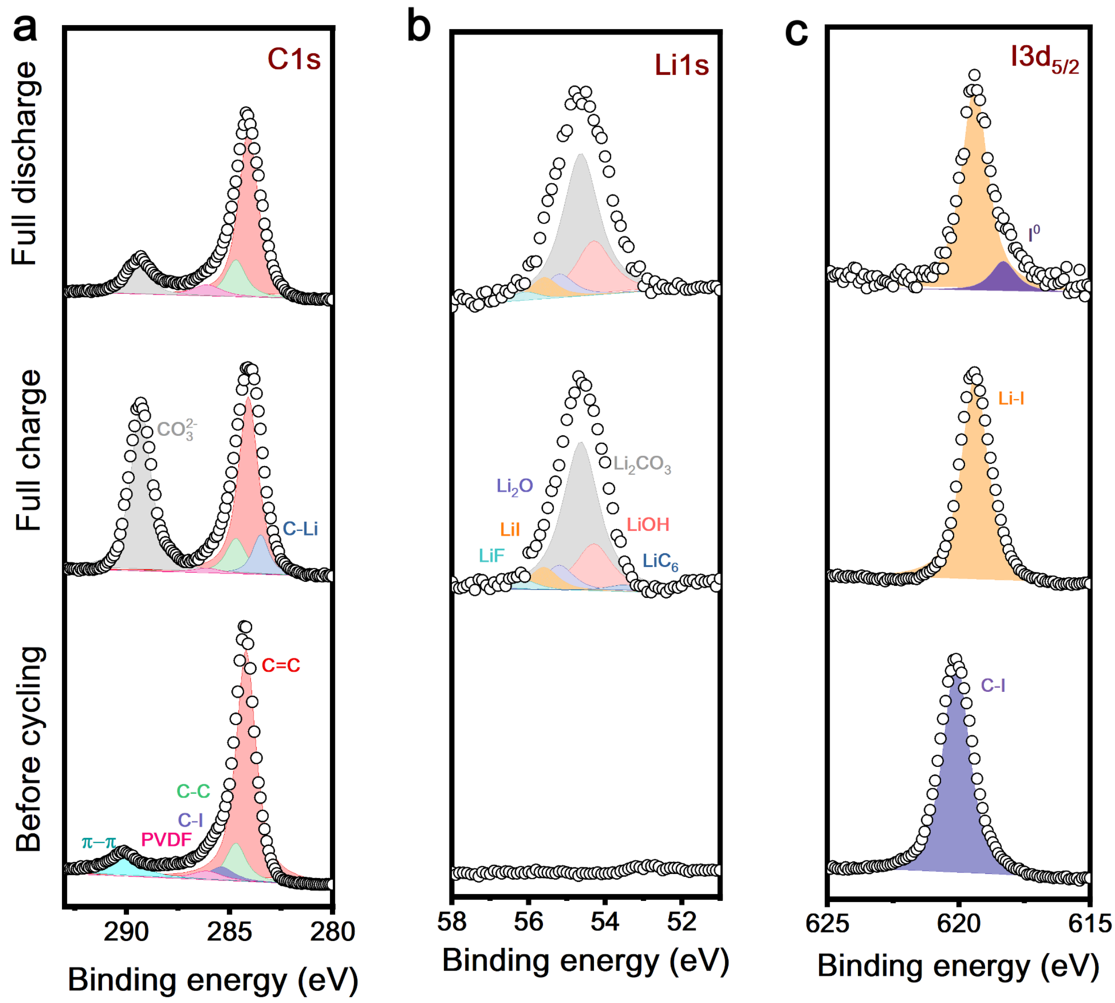

Figure S28. The ex situ XPS spectra of **6I-HBC** at different charge/discharge stages: (a) C1s; (b) Li1s; (c) I $3d_{5/2}$. C1s spectra: C=C (284.15 eV); C-C (284.75 eV); CH_2-CF_2/PVDF (286.05 eV); π–π (290.2 eV); C-I (285.4 eV); C-Li (283.5 eV); CO_3^{2-} (289.40 eV). Li1s spectra: LiC_6 (53.5 eV); LiOH (54.2 ev); Li_2CO_3 (54.7 eV); Li_2O (55.3eV); LiI (55.6 eV); LiF (56.06 eV). I $3d_{5/2}$ spectra: C-I $3d_{5/2}$ (620.1 eV); Li-I $3d_{5/2}$ (619.4 eV); I^0 $3d_{5/2}$ (618.3 eV).

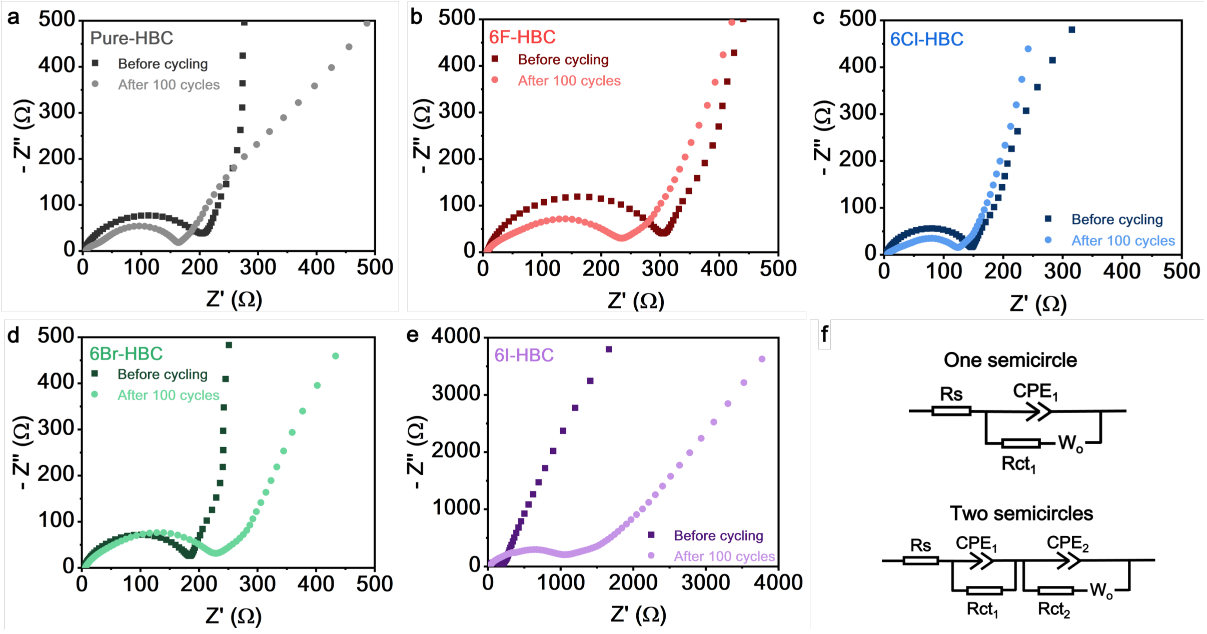

Figure S29. Nyquist plots of NGs at different cycling intervals: (a) **Pure-HBC**; (b) **6F-HBC**; (c) **6Cl-HBC**; (d) **6Br-HBC**; (e) **6I-HBC**. (f) Equivalent electrical circuit used to fit EIS data.

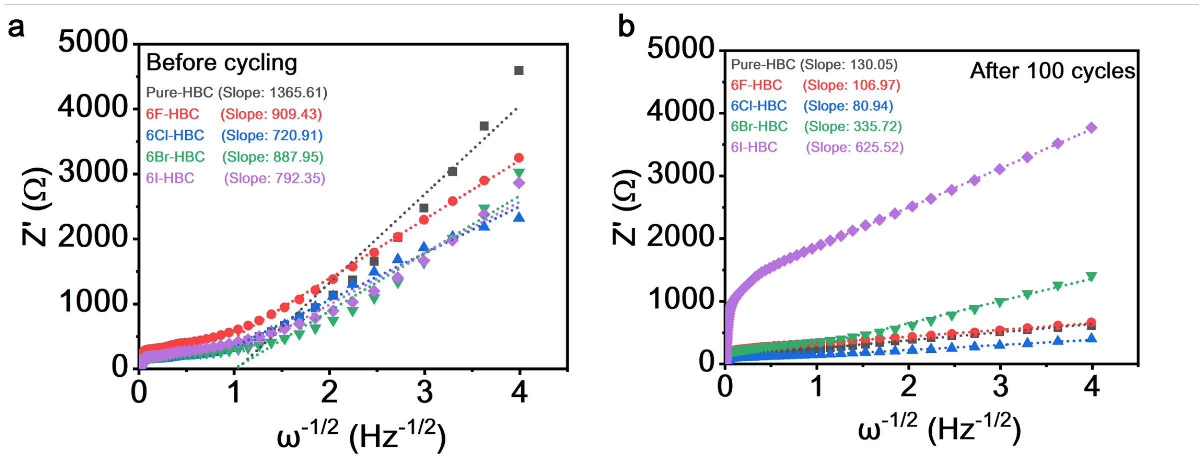

Figure S30. The Warburg impedance (σ) of NGs as a slope function of Z′ vs $\omega^{-1/2}$ at different cycling intervals: (a) before cycling; (b) after 100 cycles.

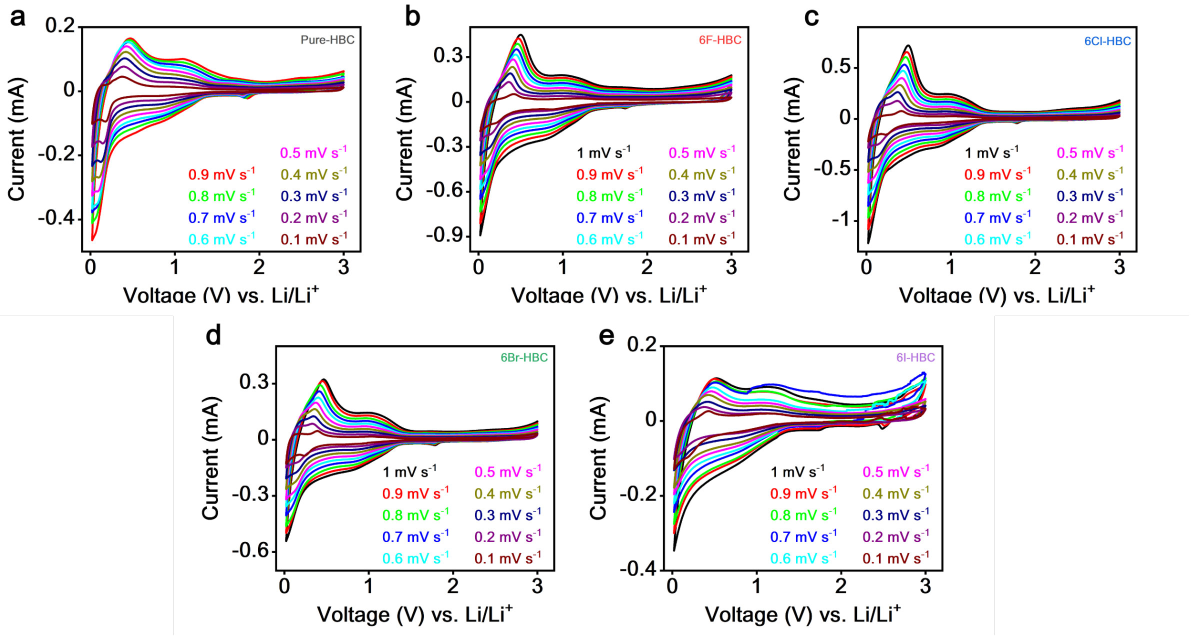

Figure S31. Sweep rate CV of NGs at different scan rate. (a) **Pure-HBC**; (b) **6F-HBC**; (c) **6Cl-HBC**; (d) **6Br-HBC**; (e) **6I-HBC**.

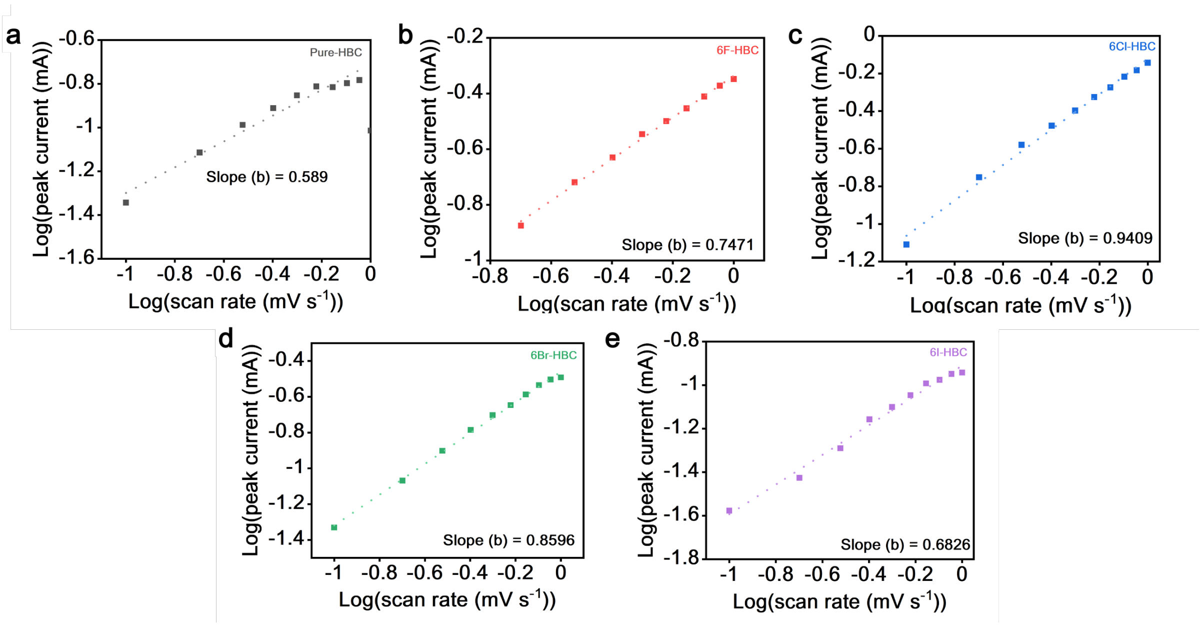

Figure S32. Log peak current (i) against log scan rate (v) plot of NGs at different scan rate. (a) **Pure-HBC**; (b) **6F-HBC**; (c) **6Cl-HBC**; (d) **6Br-HBC**; (e) **6I-HBC**.

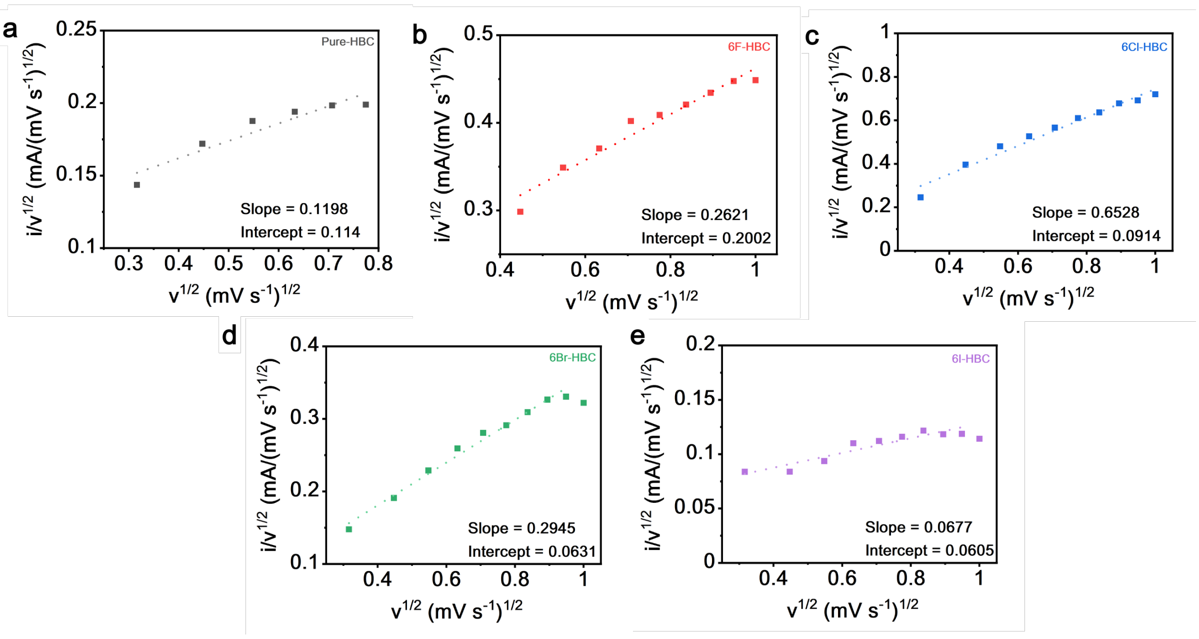

Figure S33. The $i/v^{1/2}$ vs $v^{1/2}$ plot of NGs at different scan rate. (a) **Pure-HBC**; (b) **6F-HBC**; (c) **6Cl-HBC**; (d) **6Br-HBC**; (e) **6I-HBC**.

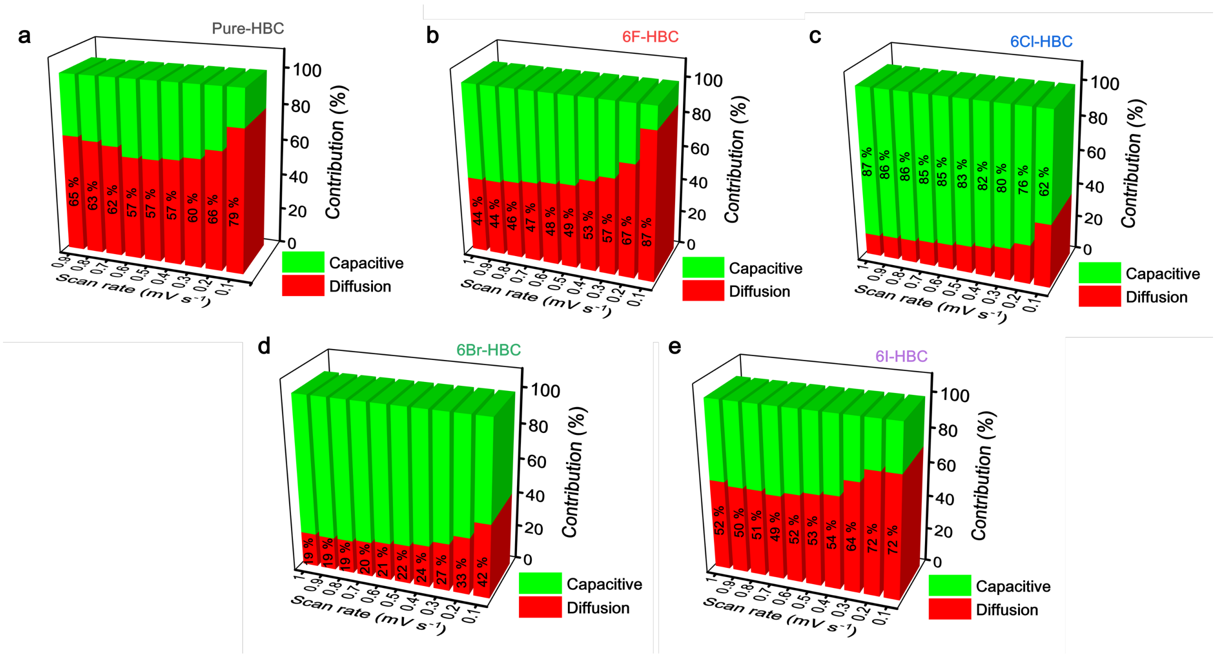

Figure S34. Estimated diffusive and capacitive contributions of NGs at different scan rate. (a) **Pure-HBC**; (b) **6F-HBC**; (c) **6Cl-HBC**; (d) **6Br-HBC**; (e) **6I-HBC**.

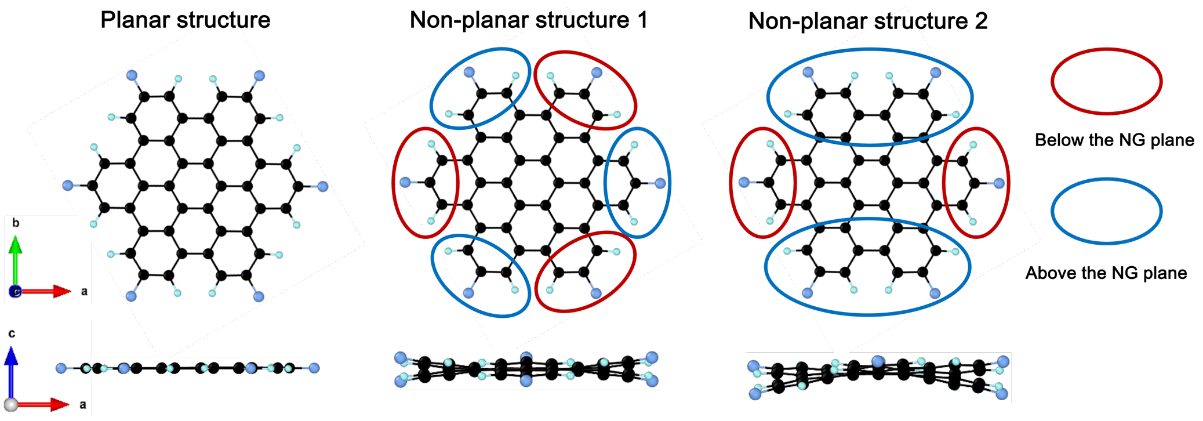

Figure S35. Top and side view of planar and non-planar shapes of halogenated-NGs with one part of the structure below the plane (red), or above the plane (blue).

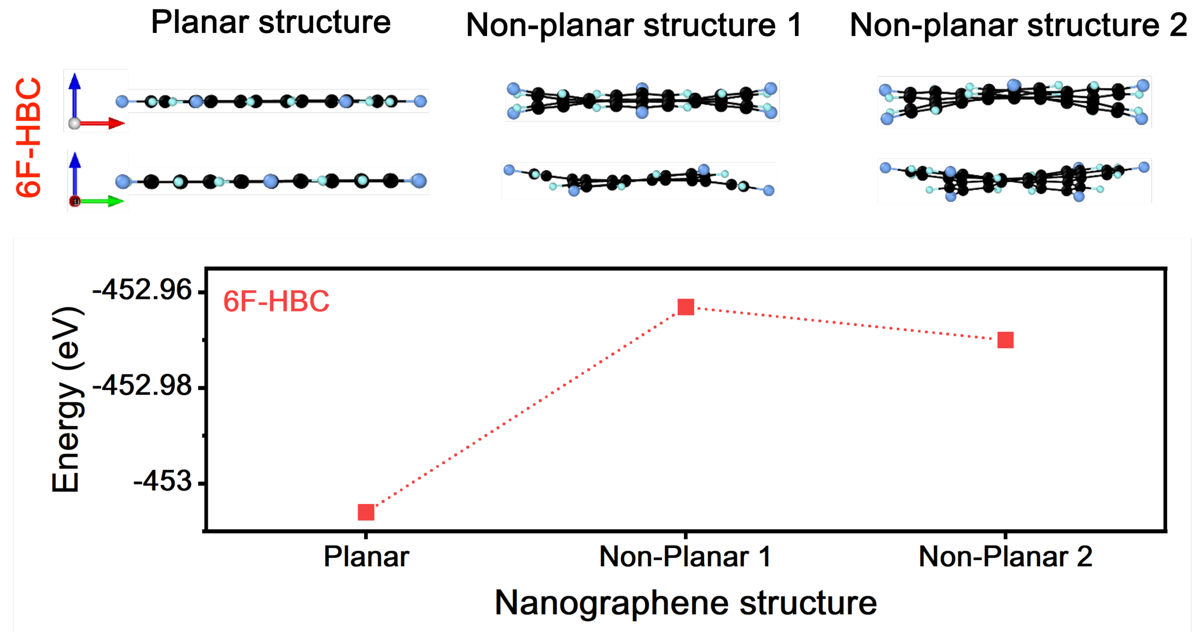

Figure S36. Comparison between planar and non-planar shapes of **6F-HBC**.

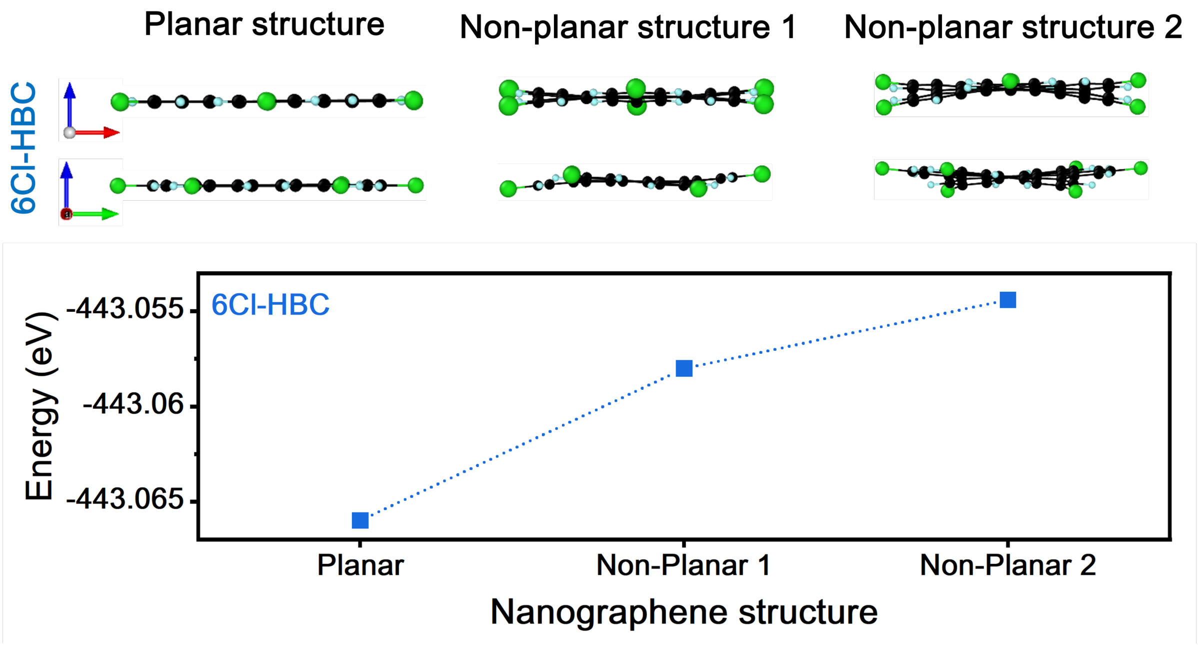

Figure S37. Comparison between planar and non-planar shapes of **6Cl-HBC**.

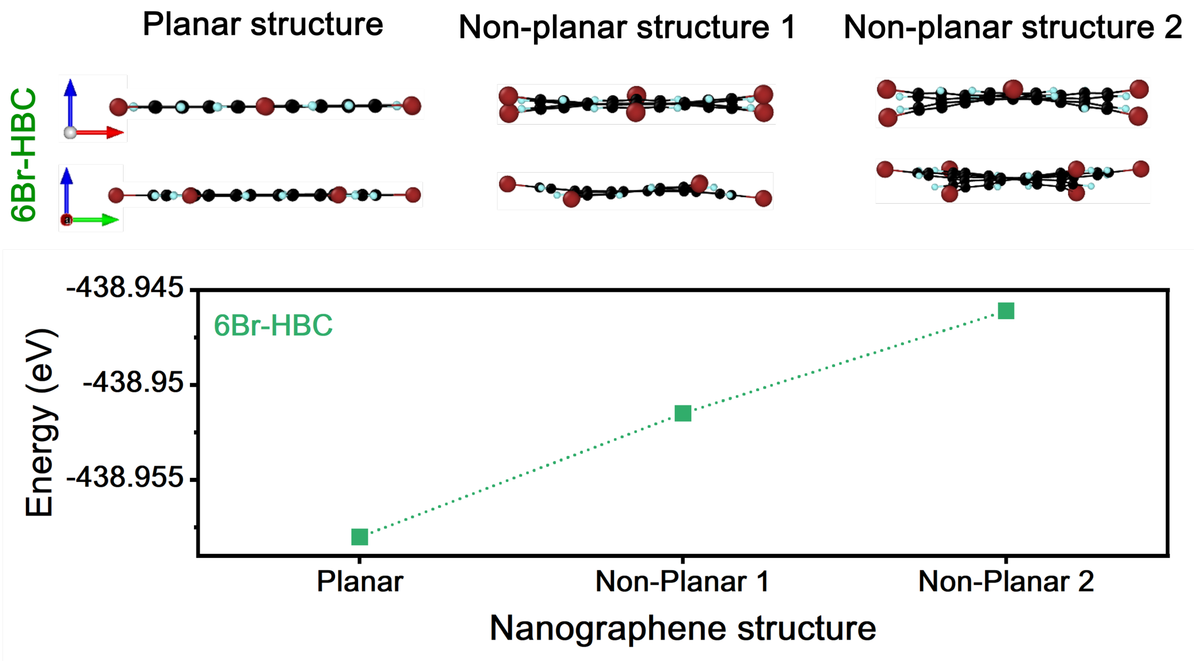

Figure S38. Comparison between planar and non-planar shapes of **6Br-HBC**.

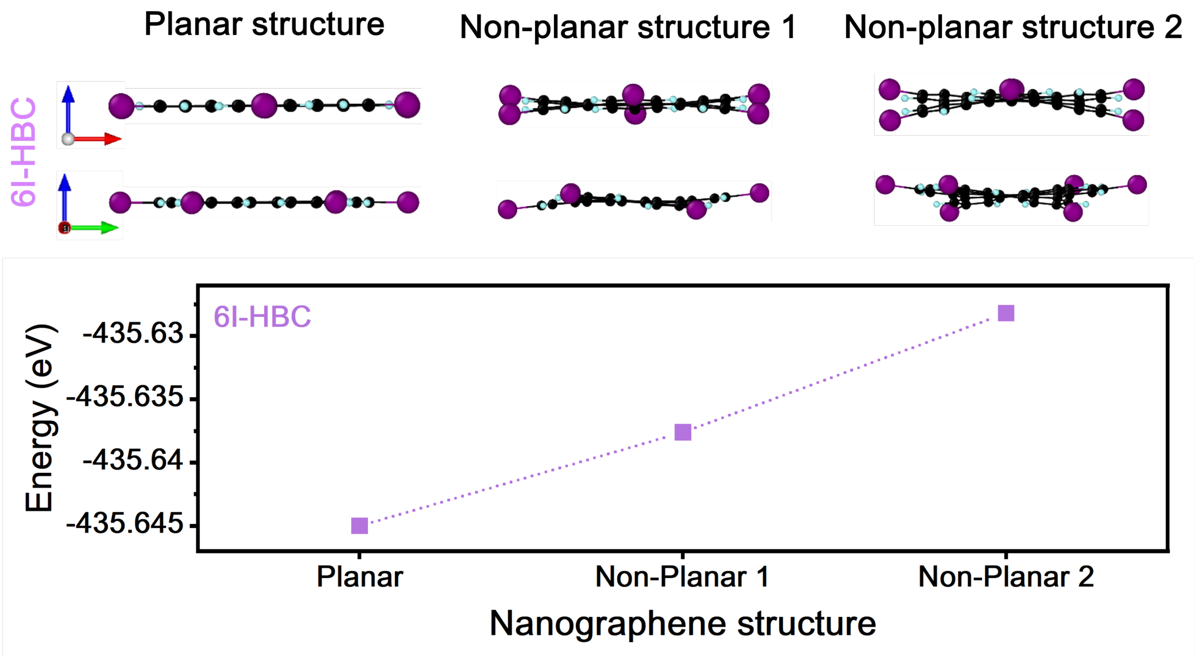

Figure S39. Comparison between planar and non-planar shapes of **6I-HBC**.

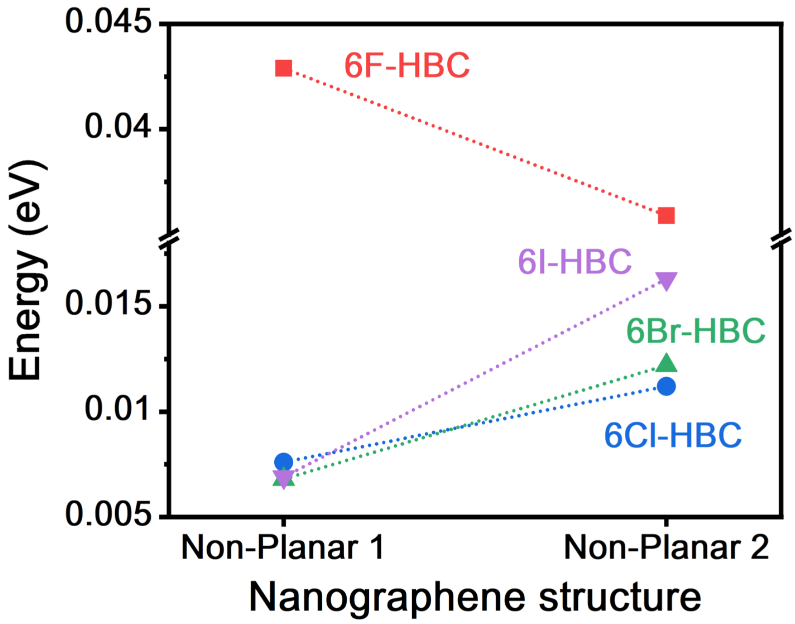

Figure S40. Energy difference between planar and respective non-planar shapes in the halogenated-NG flakes.

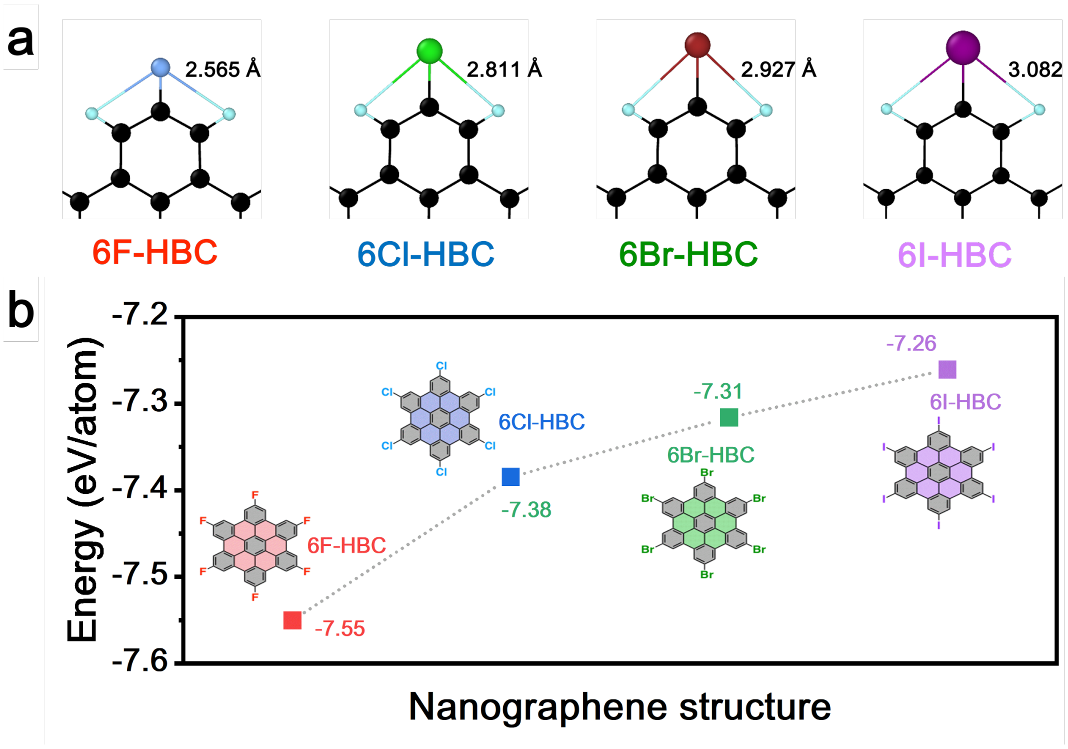

Figure S41. (a) Intramolecular hydrogen bond lengths within halogenated-NGs. (b) Ground state energies of planar-shaped halogenated-NGs in eV/atom units.

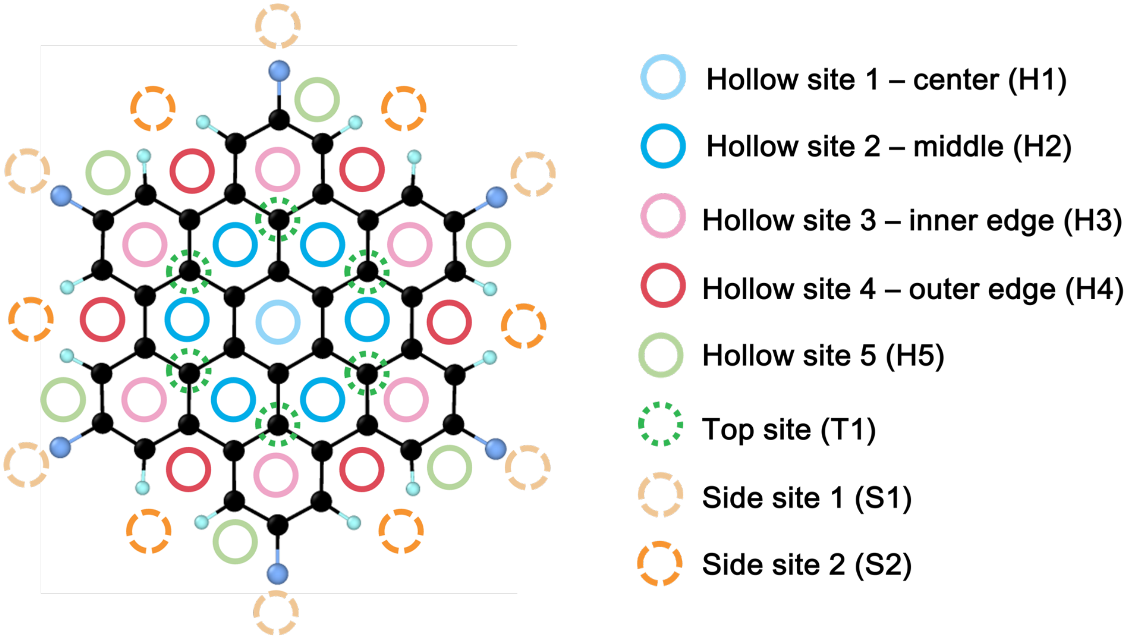

Figure S42. Identified potential adsorption sites within and around halogenated-NGs.

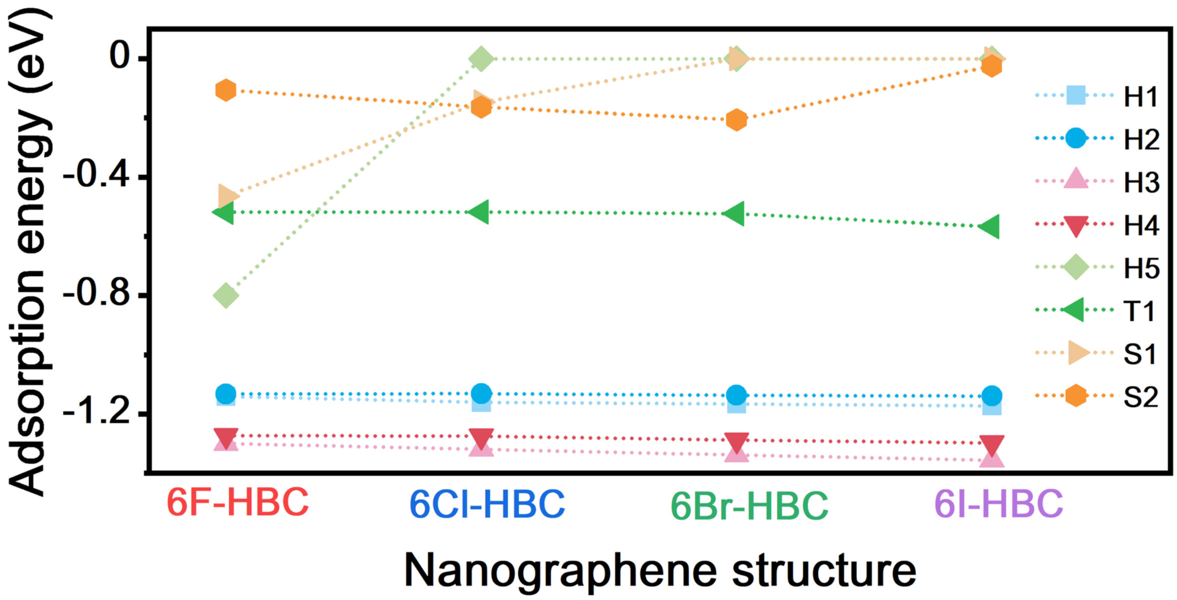

Figure S43. Adsorption energy of Li atom on the identified adsorption sites for halogenated-NGs.

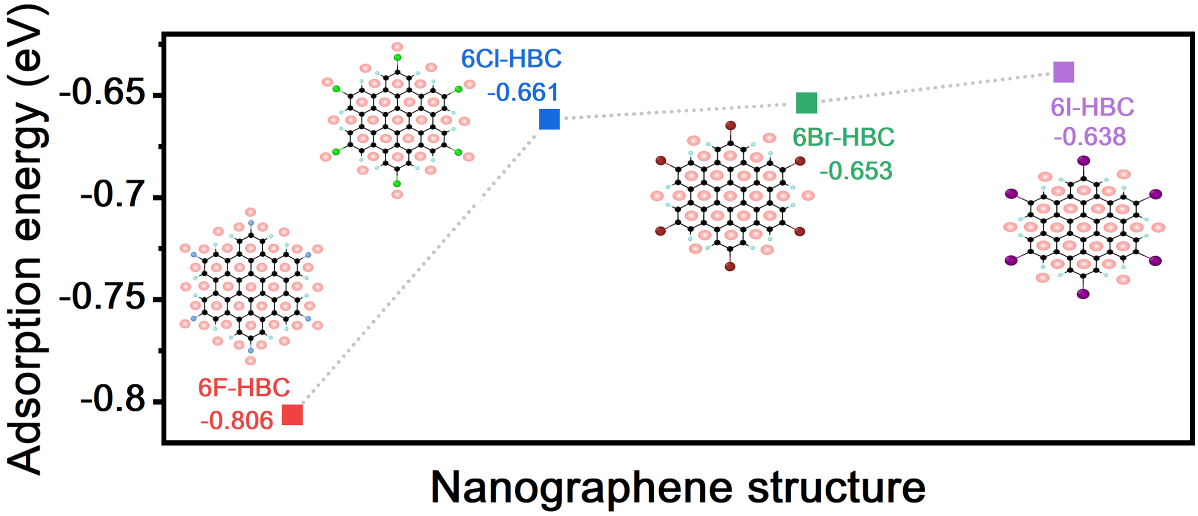

Figure S44. Average adsorption energy of Li atom on identified adsorption sites for halogenated-NGs. Inset figure is represent Li adsorption.

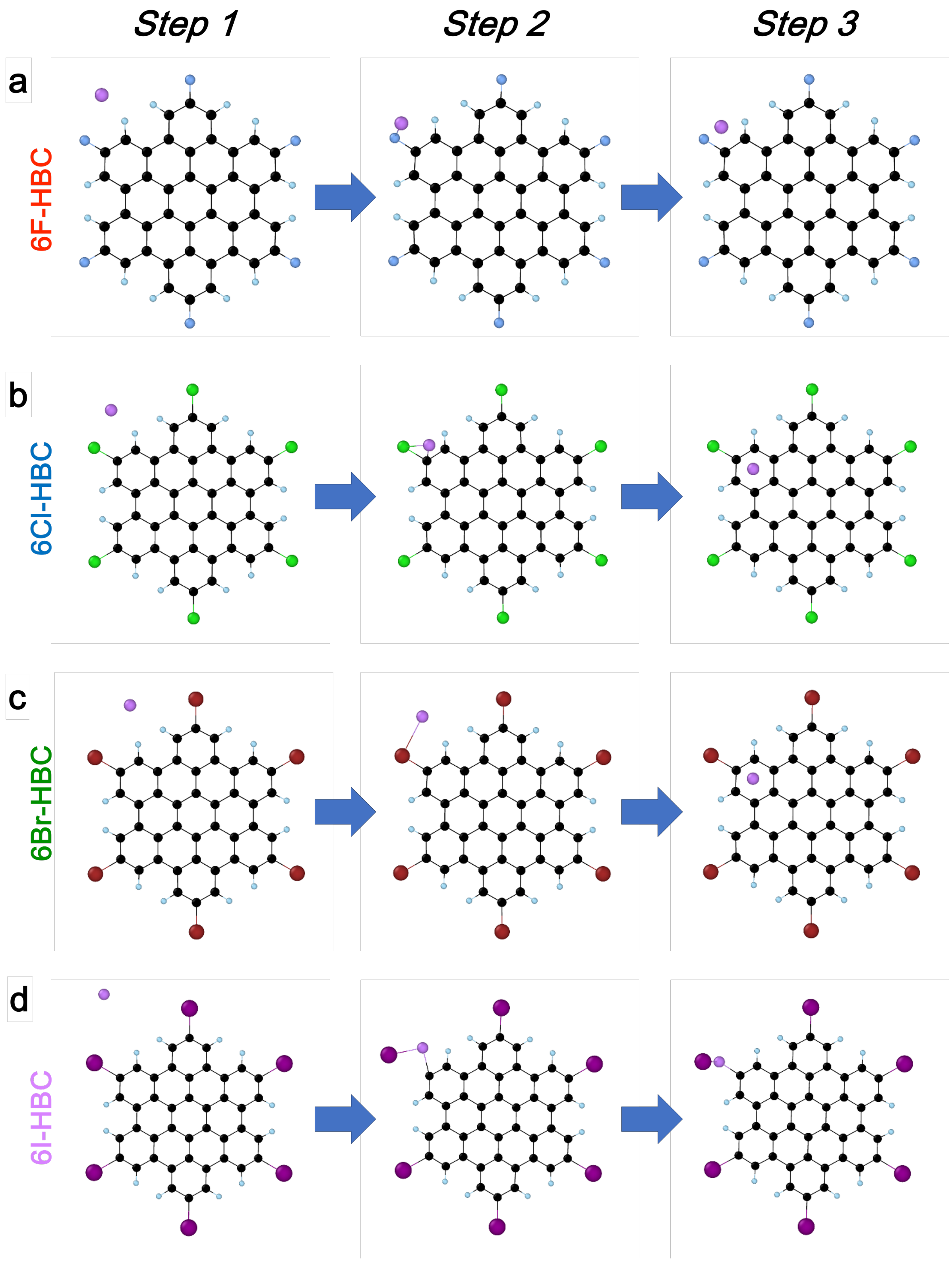

Figure S45. Li^+ adsorption pathway. a) **6F-HBC**; (b) **6Cl -HBC**; (c) **6Br-HBC**; (d) **6I-HBC.**

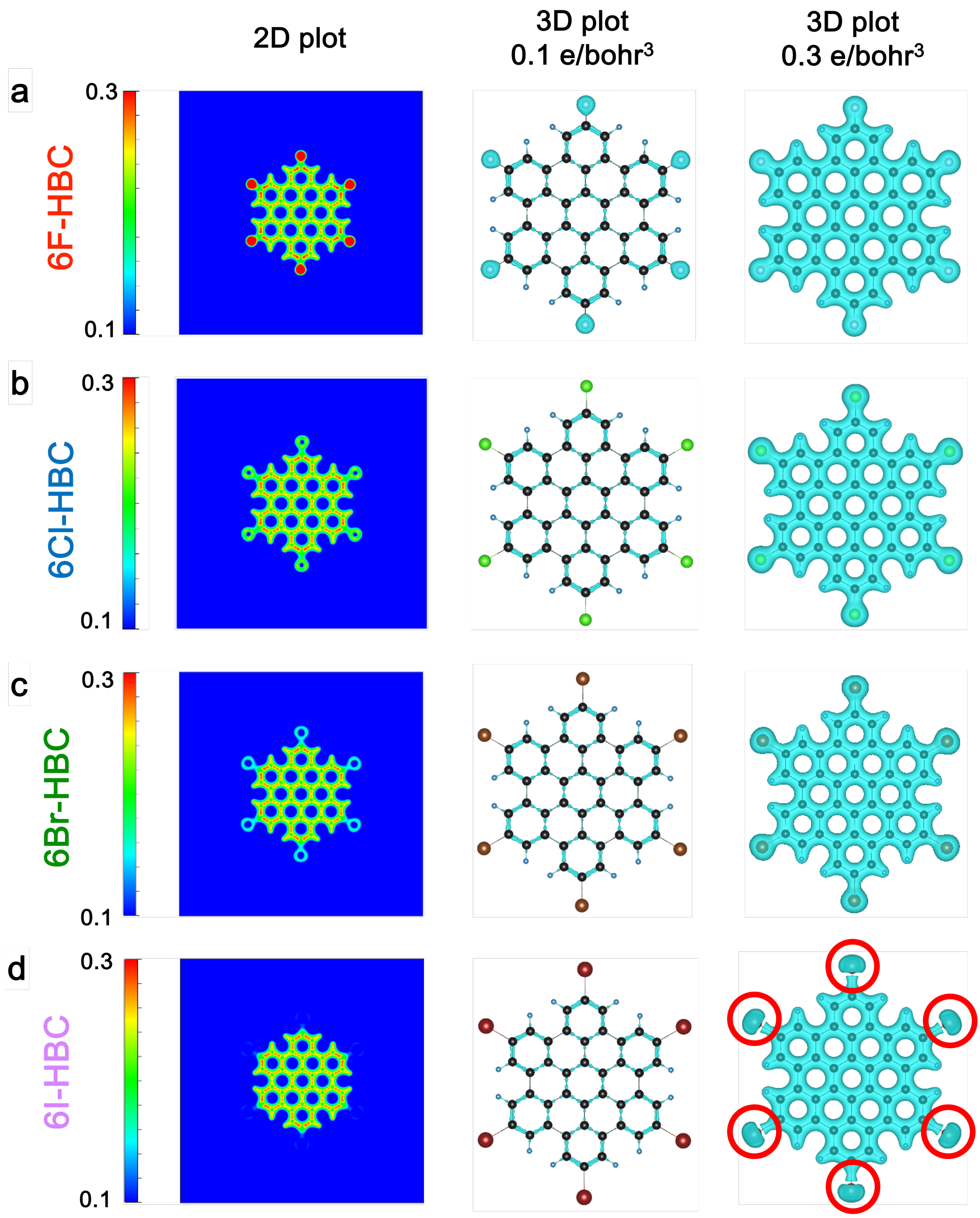

Figure S46. Charge density plot of halogenated-NGs (2D plot; 3D 0.1 e/bohr^3; and 3D 0.3 e/bohr^3). a) **6F-HBC**; (b) **6Cl -HBC**; (c) **6Br-HBC**; (d) **6I-HBC.**

Table S1. Fitted data from EIS spectra of NGs electrode at different cycling intervals.

2D NGs	Stages	*Rs* [Ω]	*Rct*$_a$ [Ω]	*Rct*$_b$ [Ω]	Diffusion coefficient [cm^2 s^{-1}]
**Pure-HBC**	Before cycling	2.96	-	161.23	1.11×10^{-14}
	After 100 cycles	7.38	50.05	136.64	1.36×10^{-12}
**6F-HBC**	Before cycling	6.78	-	252.26	6.91×10^{-14}
	After 100 cycles	7.21	49.09	153.67	2.68×10^{-12}
**6Cl-HBC**	Before cycling	6.78	-	135.13	2.96×10^{-13}
	After 100 cycles	6.35	13.92	92.98	4.22×10^{-12}
**6Br-HBC**	Before cycling	4.06	-	151.95	2.47×10^{-14}
	After 100 cycles	6.20	26.10	196.1	4.47×10^{-13}
**6I-HBC**	Before cycling	4.11	-	173.63	3.53×10^{-14}
	After 100 cycles	24.05	1140.96	-	4.33×10^{-14}

Table S2. Adsorption energy values (eV) for single Li atoms on individual sites.

Potential adsorption sites	Adsorption energy (eV)			
	**6F-HBC**	**6Cl-HBC**	**6Br-HBC**	**6I-HBC**
**H1**	-1.1403	-1.1599	-1.1657	-1.1717
**H2**	-1.1319	-1.1306	-1.1364	-1.1389
**H3**	-1.2988	-1.3184	-1.338	-1.3553
**H4**	-1.272	-1.2739	-1.2872	-1.2969
**H5**	-0.799	0	0	0
**T1**	-0.5178	-0.5172	-0.5231	-0.5669
**S1**	-0.4636	-0.1464	0	0
**S2**	-0.1055	-0.1627	-0.206	-0.0237
**Average adsorption energy**	-0.8063	-0.6617	-0.6537	-0.6386

Supplementary References

(1) Wang, D.-W.; Sun, C.; Zhou, G.; Li, F.; Wen, L.; Donose, B. C.; Lu, G. Q.; Cheng, H.-M.; Gentle, I. R. The examination of graphene oxide for rechargeable lithium storage as a novel cathode material. *J. Mater. Chem. A* **2013,** *1* (11), 3607-3612.

(2) Yen, H.-J.; Tsai, H.; Zhou, M.; Holby, E. F.; Choudhury, S.; Chen, A.; Adamska, L.; Tretiak, S.; Sanchez, T.; Iyer, S.; Zhang, H.; Zhu, L.; Lin, H.; Dai, L.; Wu, G.; Wang, H.-L. Structurally Defined 3D Nanographene Assemblies via Bottom-Up Chemical Synthesis for Highly Efficient Lithium Storage. *Adv. Mater.* **2016,** *28* (46), 10250-10256.

(3) Bar‐Tow, D.; Peled, E.; Burstein, L. A Study of Highly Oriented Pyrolytic Graphite as a Model for the Graphite Anode in Li‐Ion Batteries. *J. Electrochem. Soc.* **1999,** *146* (3), 824-832.

(4) Liu, Y.; Artyukhov, V. I.; Liu, M.; Harutyunyan, A. R.; Yakobson, B. I. Feasibility of Lithium Storage on Graphene and Its Derivatives. *J. Phys. Chem. Lett.* **2013,** *4* (10), 1737-1742.

(5) Huang, X.; Liu, Y.; Liu, C.; Zhang, J.; Noonan, O.; Yu, C. Rechargeable aluminum–selenium batteries with high capacity. *Chem. Sci.* **2018,** *9* (23), 5178-5182.

(6) Nguyen, T. Q.; Breitkopf, C. Determination of Diffusion Coefficients Using Impedance Spectroscopy Data. *J. Electrochem. Soc.* **2018,** *165* (14), E826-E831.

(7) Osaka, T.; Momma, T.; Mukoyama, D.; Nara, H. Proposal of novel equivalent circuit for electrochemical impedance analysis of commercially available lithium ion battery. *J. Power Sources* **2012,** *205*, 483-486.

(8) Cho, H.-M.; Choi, W.-S.; Go, J.-Y.; Bae, S.-E.; Shin, H.-C. A study on time-dependent low temperature power performance of a lithium-ion battery. *J. Power Sources* **2012,** *198*, 273-280.

(9) Rodrigues, S.; Munichandraiah, N.; Shukla, A. K. AC impedance and state-of-charge analysis of a sealed lithium-ion rechargeable battery. *J. Solid State Electrochem.* **1999,** *3* (7), 397-405.

(10) Barsoukov, E.; Macdonald, J. R. *Impedance Spectroscopy: Theory, Experiment, and Applications*, Wiley: 2018.

(11) Bard, A. J.; Faulkner, L. R. *Electrochemical Methods: Fundamentals and Applications, 2nd Edition*, Wiley Textbooks: 2000.

(12) Wang, J.; Polleux, J.; Lim, J.; Dunn, B. Pseudocapacitive Contributions to Electrochemical Energy Storage in TiO_2 (Anatase) Nanoparticles. *J. Phys. Chem. C* **2007,** *111* (40), 14925-14931.

(13) Li, F.-F.; Gao, J.-F.; He, Z.-H.; Kong, L.-B. Design and Synthesis of CoP/r-GO Hierarchical Architecture: Dominated Pseudocapacitance, Fasted Kinetics Features, and Li-Ion Capacitor Applications. *ACS Appl. Energy Mater.* **2020,** *3* (6), 5448-5461.

(14) Augustyn, V.; Come, J.; Lowe, M. A.; Kim, J. W.; Taberna, P.-L.; Tolbert, S. H.; Abruña, H. D.; Simon, P.; Dunn, B. High-rate electrochemical energy storage through Li+ intercalation pseudocapacitance. *Nat. Mater.* **2013,** *12* (6), 518-522.

(15) Yu, F.; Liu, Z.; Zhou, R.; Tan, D.; Wang, H.; Wang, F. Pseudocapacitance contribution in boron-doped graphite sheets for anion storage enables high-performance sodium-ion capacitors. *Mater. Horiz.* **2018,** *5* (3), 529-535.

(16) Lindström, H.; Södergren, S.; Solbrand, A.; Rensmo, H.; Hjelm, J.; Hagfeldt, A.; Lindquist, S.-E. Li^+ Ion Insertion in TiO_2 (Anatase). 2. Voltammetry on Nanoporous Films. *J. Phys. Chem. B* **1997,** *101* (39), 7717-7722.

(17) Liu, T. C. Behavior of Molybdenum Nitrides as Materials for Electrochemical Capacitors. *J. Electrochem. Soc.* **1998,** *145* (6), 1882.

(18) Garay-Tapia, A. M.; Romero, A. H.; Barone, V. Lithium Adsorption on Graphene: From Isolated Adatoms to Metallic Sheets. *J. Chem. Theory Comput.* **2012,** *8* (3), 1064-1071.

(19) Abbas, S. A.; Chen, H.-A.; Mohapatra, A.; Singh, A.; Li, S.; Pao, C.-W.; Chu, C. W. Sweetening Lithium Metal Interface by High Surface and Adhesive Energy Coating of Crystalline α-d-Glucose Film to Inhibit Dendrite Growth. *Small* **2022,** *18* (27), 2201349.

(20) Bachman, J. C.; Kavian, R.; Graham, D. J.; Kim, D. Y.; Noda, S.; Nocera, D. G.; Shao-Horn, Y.; Lee, S. W. Electrochemical polymerization of pyrene derivatives on functionalized carbon nanotubes for pseudocapacitive electrodes. *Nat. Commun.* **2015,** *6* (1), 7040.

(21) Rodríguez-Pérez, I. A.; Jian, Z.; Waldenmaier, P. K.; Palmisano, J. W.; Chandrabose, R. S.; Wang, X.; Lerner, M. M.; Carter, R. G.; Ji, X. A Hydrocarbon Cathode for Dual-Ion Batteries. *ACS Energy Letters* **2016,** *1* (4), 719-723.

(22) Mukai, K.; Harada, M.; Kikuzawa, Y.; Mori, T.; Sugiyama, J. Electrochemical Properties of Hexa-peri-hexabenzocoronene in Nonaqueous Lithium Cell. *Electrochem. Solid-State Lett.* **2011,** *14* (4), A52.
